# Connexin26 Modulates the Radiosensitivity of Cutaneous Squamous Cell Carcinoma by Regulating the Activation of the MAPK/NF-κB Signaling Pathway

**DOI:** 10.3389/fcell.2021.672571

**Published:** 2021-07-05

**Authors:** Minqiong Sun, Yuan Li, Jing Qian, Siwei Ding, Mingyu Sun, Bowen Tan, Ye Zhao

**Affiliations:** ^1^Teaching and Research Section of Nuclear Medicine, School of Basic Medical Sciences, Anhui Medical University, Hefei, China; ^2^Center of Medical Physics and Technology, Hefei Institutes of Physical Science, Chinese Academy of Sciences, Hefei, China

**Keywords:** Connexin 26, cutaneous squamous cell carcinoma, radiosensitivity, MAPK/NF-κB signaling pathway, radiotherapy

## Abstract

Previous studies have confirmed that the gap junction protein Connexin26 (Cx26) is specifically expressed in human skin tissue. Cx26 can transmit radiation-induced damage signals. However, no study has yet reported whether Cx26 expression affects the radiosensitivity of human skin squamous cancer cells or the mechanism by which this occurs. In this study, we found that human skin squamous cell carcinoma cells (A431 cells) expressed significantly more Cx26 and were more sensitive to radiation compared to normal human keratinocytes (HaCaT cells). Knockdown of Cx26 in A431 cells (A431^Cx26–/–^) decreased radiosensitivity relative to control cells and altered the expression of key proteins in the MAPK and NF-κB signaling pathways. These results demonstrate that Cx26 expression might play an important role in mediating radiation damage in A431 cells and could serve as a potential target for clinical radiotherapy for cutaneous squamous cell carcinoma.

## Introduction

Squamous cell carcinoma of the skin, also known as cutaneous squamous cell carcinoma (cSCC), is a common malignant tumor of the skin. It originates from malignant lesions of the epidermis or accessory keratinocytes of the skin. cSCC accounts for four-fifths of basal cell carcinomas. Epidemiological data indicate that the ratio of chronic squamous cell carcinoma to basal cell carcinoma has been increasing in recent years. Because hematologic and lymphoid metastases occur early on in disease progression, approximately 5% of high-risk cSCC patients exhibit distant metastases at the time of diagnosis (Di et al., 2001). Although surgery is the first treatment choice for cSCC, radiotherapy is still useful in certain patients with unresectable tumors (Waldman and Schmults, 2019). Furthermore, adjuvant radiotherapy is widely used after surgery to improve therapeutic outcomes. Therefore, it is crucial to understand the mechanisms underlying the radiosensitivity of cSCC in order to improve the effects of radiotherapy for this disease.

Gap junction-mediated intercellular communication (GJIC) plays an important role in the coordination of normal keratinocyte differentiation. Connexins are expressed in the epidermis, with Connexin26 (Cx26) specifically expressed in the basal layer of the epidermis (Sáez et al., 2005). Previous experimental results reported that overexpression of Cx26 in HeLa human cervical cancer cells (HeLa^Cx26+/+^) significantly enhanced the ability of these cells to induce and transmit radiation damage relative to normal HeLa cells. Further, HeLa^Cx26+/+^ cells displayed more rapid and intense radiation-induced bystander effects (RIBEs). Cx26 can interact with MLTK upstream of the MAPK signaling pathway, thereby affecting expression of the downstream molecules p38 and COX-2 (Zhao et al., 2014). In human normal skin keratinocytes (HaCaT cells), Cx26 expression levels affect both radiosensitivity and the activity of the MAPK and NF-κB signaling pathways.

Radiotherapy is a common treatment for cutaneous squamous cell carcinoma (cSCC); it has been used clinically for many years, and its efficacy is widely recognized. However, few studies have investigated the radiosensitivity of skin squamous cell carcinoma. In the present study, we used skin keratinocytes (HaCaT) and A431 cells to investigate the relationship between Cx26 and the radiosensitivity of skin squamous cell carcinoma cells as well as the mechanisms underlying the radiosensitivity of cSCC.

## Results

### Cx26 Expression in Cutaneous Squamous Cell Carcinoma

**FIGURE 1:**
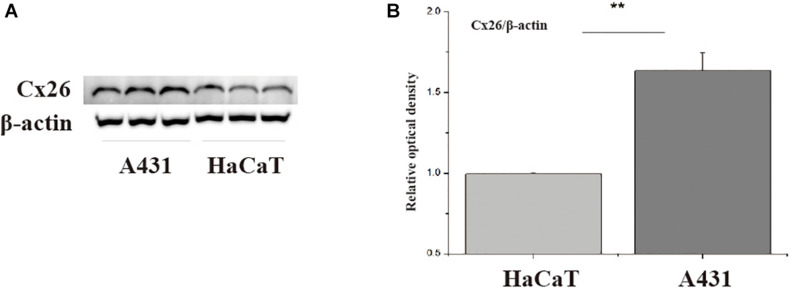
Cx26 expression levels in A431 and HaCaT cells. **(A)** Cx26 expression levels in A431 and HaCaT cells. **(B)** Quantification of Cx26 expression in A431 and HaCaT cells. **p < 0.01 between the two cell lines.

First, western blotting was used to verify Cx26 expression levels in A431 and HaCaT cells. The results showed that A431 cells expressed more Cx26 than HaCaT cells under the same conditions (Figure 1, *p* < 0.01). This result provided a theoretical basis for our subsequent experiments. Previous studies have shown that Cx26 can mediate radiation damage, therefore we chose to further explore the differences in A431 and HaCaT radiation sensitivity.

### Effects of Cx26 Expression on the Radiosensitivity of A431 Cells and HaCaT Cells

**FIGURE 2:**
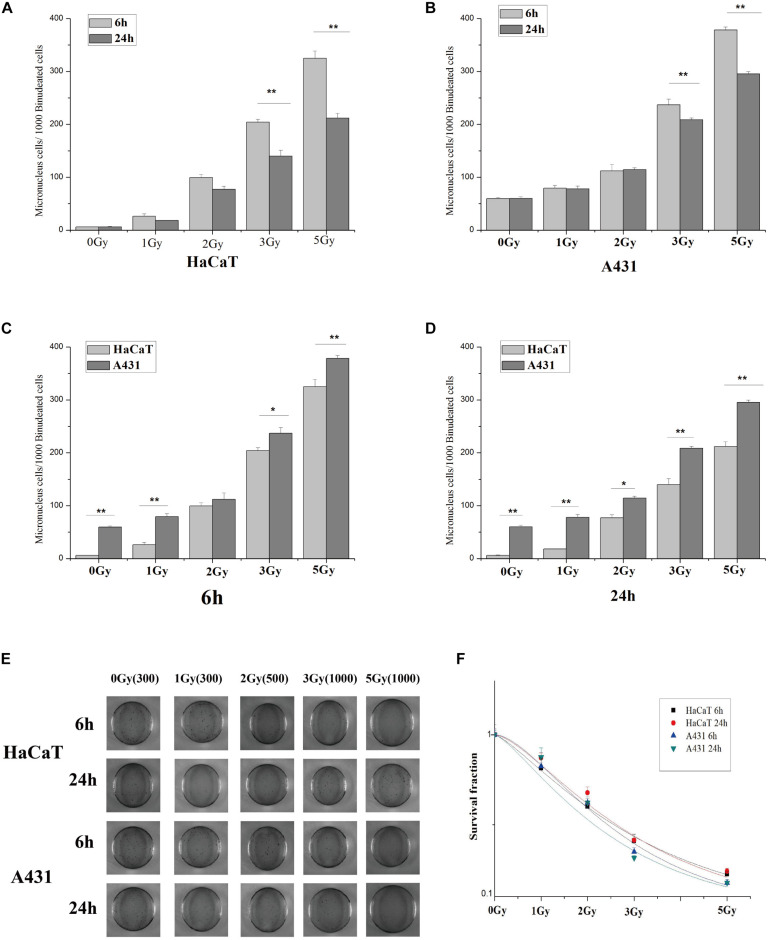
Statistical analysis and schematic diagrams of micronuclei and clone formation assays in irradiated A431 and HaCaT cells. **(A)** Comparison of micronuclei formation in HaCaT cells 6 and 24 h after irradiation. **(B)** Comparison of micronuclei formation in A431 cells 6 and 24 h after irradiation. **(C)** Comparison of micronuclei formation in HaCaT and A431 cells 6 h after irradiation. **(D)** Comparison of micronuclei formation in HaCaT and A431 cells 24 h after irradiation. **(E)** Schematic diagram showing micronuclei formation in HaCaT and A431 cells 6 and 24 h after irradiation. **(F)** Dose-survival curves for HaCaT and A431 cells 6 and 24 h after irradiation. D_0_ (HaCaT 6 h) = 2.84403, *N* = 1.32932; D_0_ (HaCaT 24 h) = 2.49758, *N* = 1.6035; D_0_ (A431 6 h) = 2.10442, *N* = 1.82945; D_0_ (A431 24 h) = 2.29352, *N* = 1.38693. *p < 0.05, **p < 0.01 between the two cell lines.

HaCaT and A431 cells were irradiated with X-rays at doses of 0, 1, 2, 3, and 5 Gy. 6 h and 24 h after irradiation, micronuclei formation and clone formation assays were performed. Our results revealed that the number of micronucleated HaCaT cells and A431 cells increased with increasing irradiation doses (Figures 2A,B). However, the number of micronuclei in A431 cells at 2 Gy, 3 Gy, and 5 Gy was significantly higher than that in HaCaT cells at the same doses (Figures 2C,D, ^∗^*p* < 0.05, ^∗∗^*p* < 0.01). The results also showed that the number of micronucleated cells was significantly lower at 24 h than at 6 h in both HaCaT cells and A431 cells. This was particularly true at the 3 Gy and 5 Gy doses (^∗∗^*p* < 0.01). In the clone formation experiments, the number of cloned cells was counted and used to calculate the fraction of surviving cells. The dose-survival curve was fitted using the multitarget model. In HaCaT cells and A431 cells, the clonal proliferation rate gradually decreased with increasing irradiation doses at both 6 and 24 h (Figure 2E). However, the dose-survival curve for A431 cells was lower than the curve for HaCaT cells at both 6 and 24 h (Figure 2F). These results showed that A431 cells were much more radiosensitive than HaCaT cells.

### Validation of Cx26 Expression in A431 Cells Following Knockdown

**FIGURE 3:**
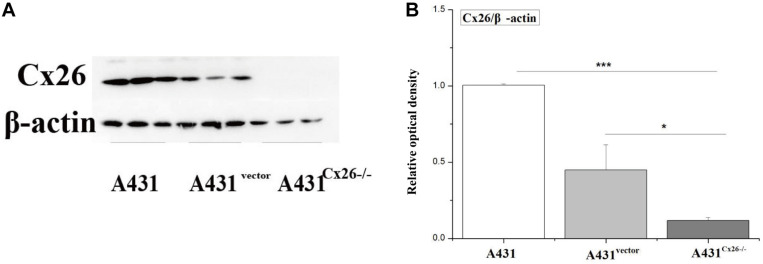
Expression of Cx26 in A431, A431^vector^, and A431^Cx26–/–^ cells. **(A)** Cx26 expression levels in A431, A431^vector^, and A431^Cx26–/–^ cells. **(B)** Quantification of Cx26 expression in A431, A431^vector^, and A431^Cx26–/–^ cells. ***p < 0.001, *p < 0.05 between each cell line.

To investigate the role of Cx26 in the radiosensitivity of A431 cells, the A431^Cx26–/–^ cell line was constructed by transfecting parental A431 cells with the Cx26 CRISPR/Cas9 plasmid. An A431^*vector*^ cell line was also constructed using an empty plasmid as a control. After transfection, protein expression was examined by western blotting. The results showed that the expression of Cx26 was significantly lower in A431^Cx26–/–^ cells as compared with the control and wild-type cells (Figure 3, ^∗∗^*p* < 0.01).

### The effect of Cx26 Expression on the Radiosensitivity of A431 Cells

**FIGURE 4:**
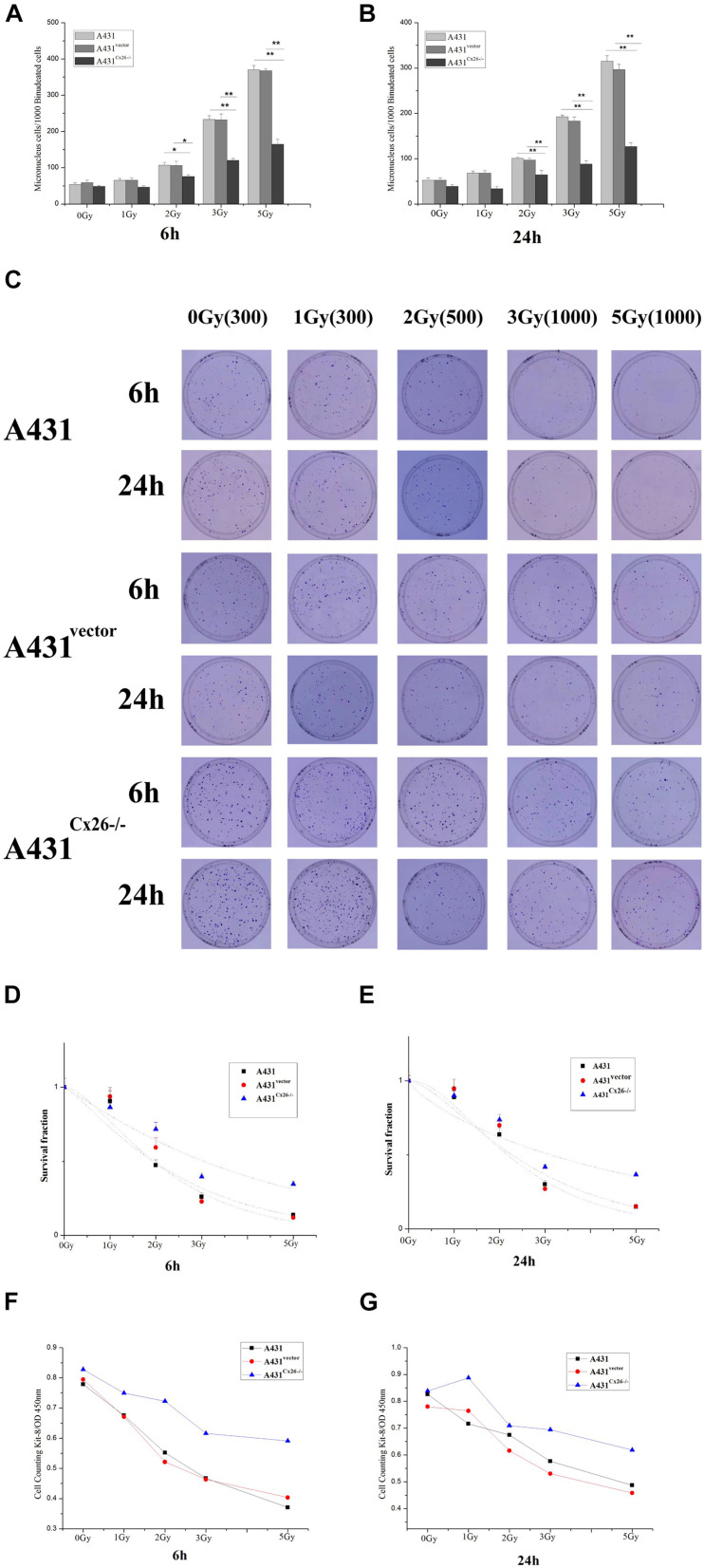
Micronuclei and colony formation assays in irradiated A431, A431^vector^, and A431^Cx26–/–^ cells. **(A)** Statistical analysis of micronuclei formation in A431, A431^vector^, and A431^Cx26–/–^ cells 6 h after irradiation. **(B)** Statistical analysis of micronuclei formation in A431, A431^vector^, and A431^Cx26–/–^ cells 24 h after irradiation. **(C)** Quantification of clone formation in A431, A431^vector^, and A431^Cx26–/–^ cells 6 and 24 h after irradiation. **(D)** Dose-survival curves for A431, A431^vector^, and A431^Cx26–/–^ cells 6 h after irradiation; D_0_ (A431 6 h) = 2.2214, *N* = 1.2845; D_0_ (A431^vector^ 6 h) = 1.6945, *N* = 1.8206; D_0_ (A431^Cx26–/–^ 6 h) = 4.0250, *N* = 1.0966. **(E)** Dose-survival curves for A431, A431^vector^, and A431^Cx26–/–^ cells 24 h after irradiation, D_0_ (A431 24 h) = 2.0667, *N* = 1.7154; D_0_ (A431^vector^ 24 h) = 1.5955, *N* = 2.3482; D_0_ (A431^Cx26–/–^ 24 h) = 5.9810, *N* = 0.7704. **(F)** OD values for A431, A431^vector^, and A431^Cx26–/–^ cells 6 h after irradiation, as detected by CCK-8 assay. **(G)** OD values for A431, A431^vector^, and A431^*Cx26–/–*^ cells 24 h after irradiation, as detected by CCK-8 assay. *p < 0.05, **p < 0.01 between each cell line.

To measure the effects of Cx26 on the radiosensitivity of A431 cells, we performed micronuclei formation, CCK-8 proliferation and colony formation assays in A431, A431^vector^ and A431^Cx26–/–^ cells 6 and 24 h after irradiation. The results showed that micronuclei formation was lower in A431^Cx26–/–^ cells at 2 Gy, 3 Gy, and 5 Gy compared with similarly treated A431 and A431^vector^ cells (Figures 4A,B). In the clone formation experiment, the D_0_ values of the A431^vector^ and A431 cells were relatively close, while the D_0_ value of the A431^Cx26–/–^ cells was significantly higher than either of these (Figures 4C–E). The CCK-8 cell proliferation experiment results showed that the viability of A431^Cx26–/–^ cells was significantly higher than that of the other two groups after 6 and 24 h irradiation. Further, radiation-induced damage was lower in A431^Cx26–/–^ cells compared with A431^vector^ and A431 cells even at increasing radiation doses (Figures 4F,G). These results suggested that A431^Cx26–/–^ cells were less radiosensitive than the other two types of cells.

### Cx26 Expression Affects the Radiation-Induced Activation of the MAPK and NF-κB Signaling Pathways in A431 Cells

**FIGURE 5:**
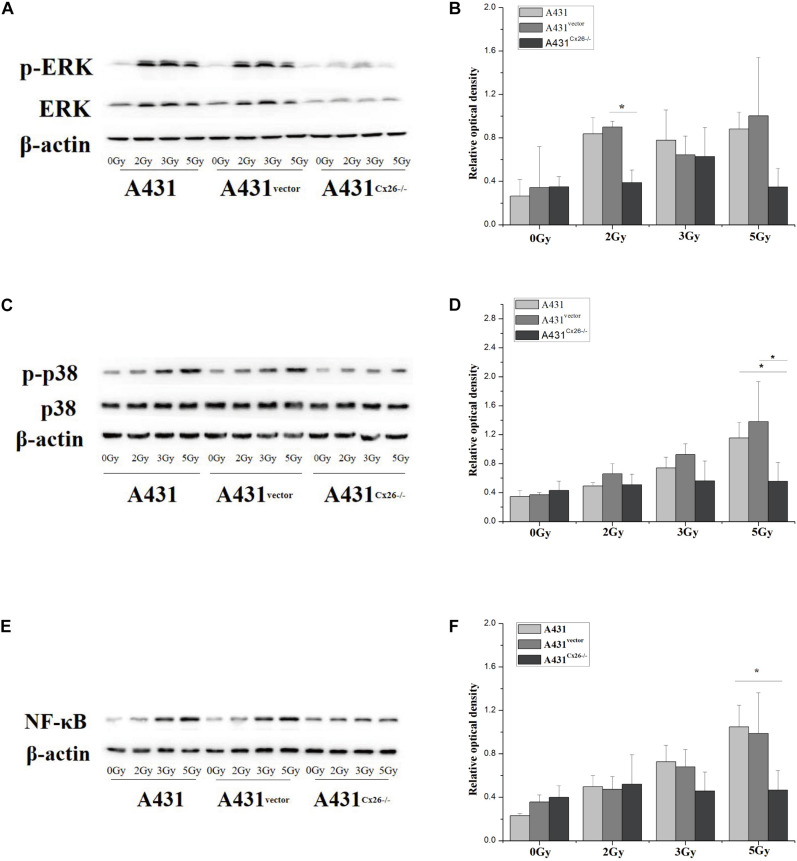
Activation of the MAPK and NF-κB signaling pathways in A431, A431^vector^, and A431^Cx26–/–^ cells after irradiation. **(A)** p-ERK/ERK expression in irradiated A431, A431^vector^, and A431^Cx26–/–^ cells. **(B)** Quantification of p-ERK/ERK expression in irradiated A431, A431^vector^, and A431^Cx26–/–^ cells. **(C)** Expression of p-p38/p38 in irradiated A431, A431^vector^, and A431^Cx26–/–^ cells. **(D)** Quantification of p-p38/p38 expression in irradiated A431, A431^vector^, and A431^Cx26–/–^ cells. **(E)** NF-κB expression in irradiated A431, A431^vector^, and A431^Cx26–/–^ cells. **(F)** Quantification of NF-κB expression in irradiated A431, A431^vector^, and A431^Cx26–/–^ cells. *p < 0.05 between each cell line.

A431, A431^vector^, and A431^Cx26–/–^ cells were X-ray irradiated with 0, 2, 3, and 5 Gy, as previously described. Treatment of A431 cells with increasing doses of irradiation altered expression of ERK but not p38. However, phospho-ERK and phospho-p38 protein expression levels were significantly increased in A431 cells after irradiation. Expression of phospho-ERK was significantly increased in A431 cells irradiated with 2 Gy, and 5 Gy compared to sham-irradiated cells (Figures 5A,B). Similarly, phospho-p38 expression was significantly higher in A431 cells after 5 Gy irradiation compared to sham-irradiated cells (Figures 5C,D). Expression of NF-κB, which is downstream of the MAPK signaling pathway, was increased in A431 cells irradiated with increasing doses, particularly 3 Gy and 5 Gy (Figures 5E,F). ERK and p38 protein expression was not significantly correlated with increased irradiation in A431^Cx26–/–^ cells, however ERK expression levels were lower in A431^Cx26–/–^ cells than in A431 cells and A431^vector^ cells. The p-ERK/ERK ratio was lower in 2 Gy-irradiated A431^Cx26–/–^ cells compared to 2 Gy-irradiated A431^vector^ cells, however there was no significant difference at 3 Gy and 5 Gy (Figures 5A,B). In addition, the expression of ERK was lower in A431^Cx26–/–^ cells compared with A431 and A431^vector^ cells. However, the expression of phospho-ERK was significantly lower in A431^Cx26–/–^ cells than in A431 and A431^vector^ cells at 2 Gy and 5 Gy; this difference was statistically significant (Supplementary Figure 2). These results indicated that the expression of Cx26 influenced the expression and activation of ERK within the MAPK signaling pathway in A431 cells. The p-p38/p38 ratio was significantly lower in 5 Gy-irradiated A431^Cx26–/–^ cells compared with similarly treated A431 cells and A431^vector^ cells (Figures 5C,D). Moreover, NF-κB protein expression in A431^Cx26–/–^ cells did not vary according to radiation dose but was significantly lower than in 5 Gy-irradiated A431 cells (Figures 5E,F). In addition, we also verified the nuclear accumulation of NF-κB (p65) in A431 and A431^Cx26–/–^ cells after irradiation. Using an immunofluorescence assay, we found that the nuclear accumulation of p65 was significantly higher in 5 Gy-irradiated A431 cells than in either sham-irradiated A431 cells or 5 Gy-irradiated A431^Cx26–/–^ cells (Supplementary Figure 1). These results suggested that after irradiation, the expression of proteins involved in the MAPK and downstream NF-κB signaling pathways was affected by Cx26 expression levels.

## Discussion

The gap junction is the contact area between adjacent cells, which can allow for the exchange and interaction of some ions and other small molecules between adjacent cells. Therefore, gap junctions play an important role in intercellular communication and are widely found in various vertebrate tissues. Interstitial junctions are expressed throughout the body and are involved in many processes necessary for normal physiological functions. An increasing number of studies have shown that interstitial junction proteins, which are the main protein unit comprising interstitial junctions, are closely related to several human diseases. The half-channel of the gap junction protein can also function on the non-gap junction membrane, allowing for the exchange of solute with the extracellular space (Richard, 2000; Sáez et al., 2005).

Gap junction-mediated intercellular communication (GJIC) plays an important role in the differentiation and coordination of epidermal keratinocytes. In rodent skin, intercellular communication is highly regulated and is mediated by at least nine different junction proteins, including Cx30, Cx30.3, Cx31, Cx26, and Cx31.1 (Richard, 2000). Among these gap junction proteins, studies have shown that mutations in Cx26 occur more frequently in human populations (Lee and White, 2009). The results of a previous study of skin diseases also showed that Cx26-mediated intercellular communication plays an important role in regulating the radiation-induced bystander effect (RIBE) between neighboring cells after irradiation. In addition, some studies have concluded that the main roles of Cx26 in GJIC are to transmit radiation damage signals, stimulate radiation-induced bystander effects, and cause irradiated cells to produce more obvious radiation damage effects, thus aggravating the DNA radiation damage response. Each of these functions could potentially be co-opted to achieve our therapeutic goals in a clinical setting (Matesic et al., 1994; Richard, 2000; Apps et al., 2009; Lee and White, 2009).

In this study, we mainly investigated the effects of Cx26 expression levels on the radiosensitivity of cutaneous squamous cell carcinoma cells. We found that Cx26 expression was significantly higher in human skin squamous cell carcinoma cells (A431 cells) than in normal skin keratinocytes (HaCaT cells). Further, the radiosensitivity of A431 cells was significantly higher than that in HaCaT cells. These results suggested that skin squamous cell carcinoma may be more radiosensitive than the epidermis of normal human skin. We further explored whether the difference in radiosensitivity between these two types of cells was related to Cx26 expression levels. A431^Cx26–/–^ cells were constructed using a CRISPR/Cas9 plasmid, and control A431^vector^ cells were constructed using an empty vector. Our results showed that A431^Cx26–/–^ cells were significantly less radiosensitive than A431 cells and A431^vector^ cells.

We also detected activation of the MAPK and NF-κB signaling pathways in A431^Cx26–/–^, A431^vector^ and A431 cells after irradiation. The downstream modulators of the MAPK signaling pathway, including ERK1/2, p38^MAPK^ and c-Jun amino-terminal kinase (JNK), have been thoroughly described (Kim, 2009). ERK1/2 is mainly activated by growth factors or mitogens, leading to cell differentiation, growth and survival, while JNK and p38 are mainly activated by oxidative stress and cytokines, leading to inflammation and apoptosis (Fan and Chambers, 2001). However, certain stimuli can cause the simultaneous activation of all three proteins. Previous studies have shown that the MAPK signaling pathway plays a role in GJIC (Rummel et al., 1999; Wada and Penninger, 2004; Kim, 2009). Differential expression of various pathway proteins can affect gene expression and cell survival and death (Upham et al., 1997; Richard, 2000; Ruch et al., 2001; Rabionet et al., 2002; Kim et al., 2009). Within the MAPK signaling pathway, the key proteins we observed, including ERK and p38, play an important role in the processes of cell proliferation, differentiation and apoptosis (Matesic et al., 1994; Hwang et al., 2005; Kim, 2009). Previous studies have shown that ERK1/2, p38^MAPK^ and JNK are significantly enhanced in the common skin disease psoriasis. Indeed, H_2_O_2_ can enter keratinocytes through aquaporin 3, activating the NF-κB signaling pathway and thus participating in the development of psoriasis. This signaling pathway further drives the formation of Th1, Th17, and keratinocytes and promotes the accumulation of inflammatory cytokines and vascular endothelial growth factor, ultimately leading to skin damage (Xu et al., 2019). Currently, there has been no specific research in this field devoted to cutaneous squamous cell carcinoma. In this study, we used GJIC-mediated radiation injury to investigate the effects of Cx26 expression on radiation sensitivity and apoptosis-related signaling pathways in A431 cells. We found that the expression of ERK was reduced in A431^Cx26–/–^ cells, but the expression of p38 and NF-κB (p65) was not significantly altered. Therefore, we speculated that ERK expression would be affected by Cx26 knockout in A431 cells. Our results showed that phospho-ERK, phospho-p38 and p65 expression levels were significantly lower in A431^Cx26–/–^ cells than in A431 or A431^vector^ cells after irradiation. The nuclear accumulation of p65 protein was also significantly higher in irradiated A431 cells than in irradiated A431^Cx26–/–^ cells. Therefore, expression of the Cx26 protein may play an important role in the regulation of the MAPK signaling pathway and the downstream NF-κB signaling pathway in A431 cells, thus affecting cell proliferation and apoptosis.

Based on the results of this study, we believe that Cx26 can transmit radiation damage signals in A431 cells and that A431 cells are highly sensitive to radiation. However, additional studies should be performed to address the relevance of this finding for the treatment of cutaneous squamous cell carcinoma. Specifically, future research should investigate how to apply radiotherapy to cancer patients to improve the anti-tumor effects and to reduce the recurrence and mortality rates of patients.

## Materials and Methods

### Cell Culture

The human immortalized keratinocyte cell line HaCaT and human skin squamous cell line A431 were gifted by the Cell Bank of Shanghai, Chinese Academy of Sciences. The A431^Cx26–/–^ cell line was generated *via* transfection of A431 cells with a Cx26 knockout CRISPR/Cas9 plasmid. The A431^vector^ cell line was constructed by transfecting A431 cells with an empty plasmid. HaCaT and A431 cells were cultured in high glucose DMEM (Gibco, Grand Island, NY, United States) containing 10% FBS. A431^Cx26–/–^ and A431^vector^ cells were cultured in high glucose DMEM containing 10% FBS and 1 μg/ml puromycin. All cells were incubated at 37°C in a humidified atmosphere containing 95% air and 5% carbon dioxide.

### Western Blot

Cells in each treatment group were scraped off the culture dishes and lysed in lysis buffer containing 10% glycerol, 10 mM Tris–HCl (pH 6.8), 1% sodium dodecyl sulfate (SDS), 5 mM dithiothreitol (DTT), and 1 × complete protease inhibitor cocktail (Sigma-Aldrich). The concentration of proteins in the supernatant were quantified and then 20 μg of each protein sample was separated on 12% SDS-polyacrylamide gels. The separated proteins were transferred to a polyvinylidene difluoride membrane and blocked for 1 h at room temperature in 5% skim milk in Tris-buffered saline (TBS) containing 0.1% Tween-20. Then, the membrane was incubated overnight at 4°C with primary antibody. The primary monoclonal antibodies used were: anti-Connexin26, anti-ERK1/2, anti-phospho-ERK1/2, anti-p38, anti-phospho-p38, and anti-NF-κB (Cell Signaling Technology, Beverly, MA, United States). After exposure, the relative optical density of the bands was analyzed in ImageJ. The data were normalized and statistically analyzed using SPSS and plotted using Origin 8.0.

### Plasmid Transfection

Cells were transfected with a mixture of Connexin26 Double Nickase Plasmid (h) (sc-402132-NIC, Santa Cruz Biotechnology, Inc., United States), transfection reagent and transfection medium (Santa Cruz Biotechnology, Inc., United States) in a certain proportion. The plasmid targets the following gene sequences for knockout: (A) tcgcattatgatcctcgttg and (B) gagccagatctttccaatgc. Then, the most effective concentration of puromycin was determined through a preliminary experiment. After 1–2 weeks of screening, monoclones were cultured for 10–14 days. The monoclones were removed and placed into 24-well plates and successfully transfected cells were selected with puromycin. Finally, the cells were amplified to form stable cell lines.

### Micronuclei Formation Assay

Micronuclei, a form of chromosomal damage that arises mainly from DNA double-strand breaks, were evaluated using the cytokinesis block technique. After irradiation, the cells were hydrolyzed with trypsin at intervals of 6 and 24 h. A total of 50,000 cells were seeded in a 35 mm dish and cultured in high glucose DMEM containing 10% FBS. After cell attachment, the medium was changed, and cytochalasin B (Sigma) was added at a final concentration of 2.5 μg/ml. After 48 h, the cells were fixed with anhydrous ethanol and stained with acridine orange for cell counting. At least 1,000 binucleated cells were examined in each dish.

### Proliferation Assay

The prepared cells were irradiated with 0, 1, 2, 3, or 5 Gy and then incubated for 6 or 24 h. Cells were seeded in 60 mm dishes according to the radiation dose received. The dishes were kept in an incubator for 10–12 days to allow for colony formation. Then, cells were fixed with anhydrous ethanol for 30 min and stained with 0.1% crystal violet solution for 2–3 h. The data were collected and analyzed in Excel to obtain the cell survival score. Then, the dose-survival curve was fitted and the D_0_ value obtained using Origin8.0.

### CCK-8 Cell Proliferation Assay

Approximately 2,000 cells were plated into each well of a 96-well plate. After stabilization, X-ray irradiation was performed at doses of 0, 1, 2, 3, and 5 Gy. After irradiation, cells were incubated for 6 or 24 h. Then, cells were incubated with 20 μl CCK-8 solution for 1.5 h. The absorbance value was measured at 450 nm using a microplate reader.

### Immunofluorescence Assay

The cells were X-ray irradiated and incubated at 37°C for 6 h. Then, the cells were washed with PBS and fixed with 4% paraformaldehyde solution. Cells were then incubated with 0.3% Triton in PBS for 30 min and blocked with 1% goat serum. Then, the cells were incubated overnight in the primary antibody. The next day, the primary antibody solution was washed off. The cells were washed 3 × 5 min with PBST and then incubated with the secondary antibody for 2 h. Then, the cells were stained with the nuclear dye Hoechst 33342 (Sigma). Finally, fluorescence microscopy was performed, and images were taken at random in each dish.

### Statistical Analyses

Data were statistically analyzed using SPSS software. The independent sample *t*-test was used for comparisons between two groups. Comparisons between multiple groups were performed using one-way analysis of variance *p* < 0.05. A value of *p* < 0.05 was considered to be statistically significant. All experiments in this study were performed in both technical and biological triplicate.

## Data Availability Statement

The original contributions presented in the study are included in the article/Supplementary Material, further inquiries can be directed to the corresponding author/s.

## Author Contributions

YZ conceived this study and designed. MQS and YL conducted the main experiments. JQ, SD, MYS, and BT also conducted some experiments. MQS and YL analyzed the experimental data. MQS and YZ prepared the manuscript. All authors contributed to the article and approved the submitted version.

## Conflict of Interest

The authors declare that the research was conducted in the absence of any commercial or financial relationships that could be construed as a potential conflict of interest.
