# Supplementary figures and images for: Connexin26 Modulates the Radiosensitivity of Cutaneous Squamous Cell Carcinoma by Regulating the Activation of the MAPK/NF-κB Signaling Pathway
Source: Front Cell Dev Biol. 2021 Jul 5;9:672571. doi: 10.3389/fcell.2021.672571 (PMC8287175; doi:10.3389/fcell.2021.672571)

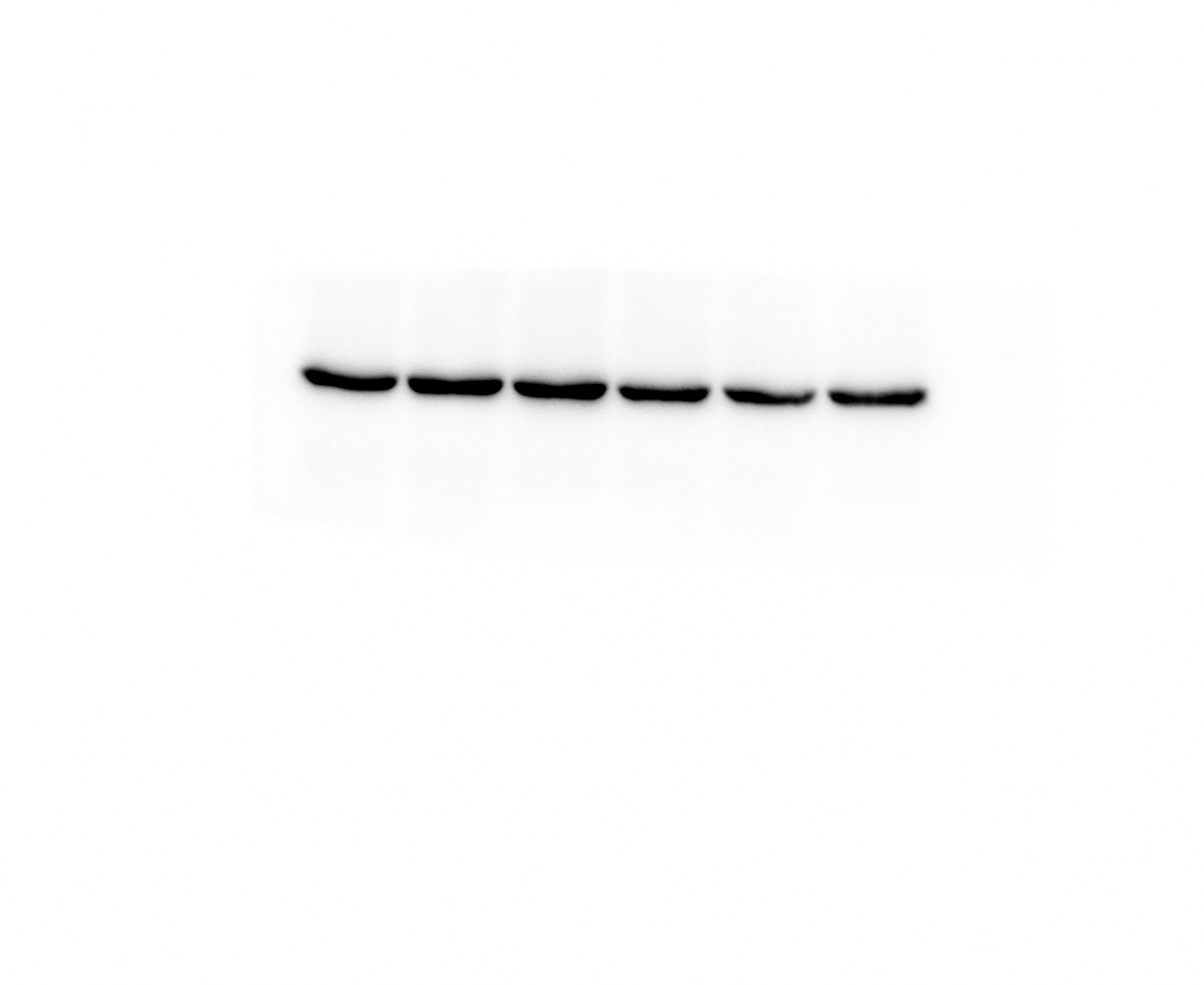

Supplement: Supplementary file 1 [file Data_Sheet_1.ZIP › data/Cx26 in A431 cells and HaCaT cells/A actin.jpg]

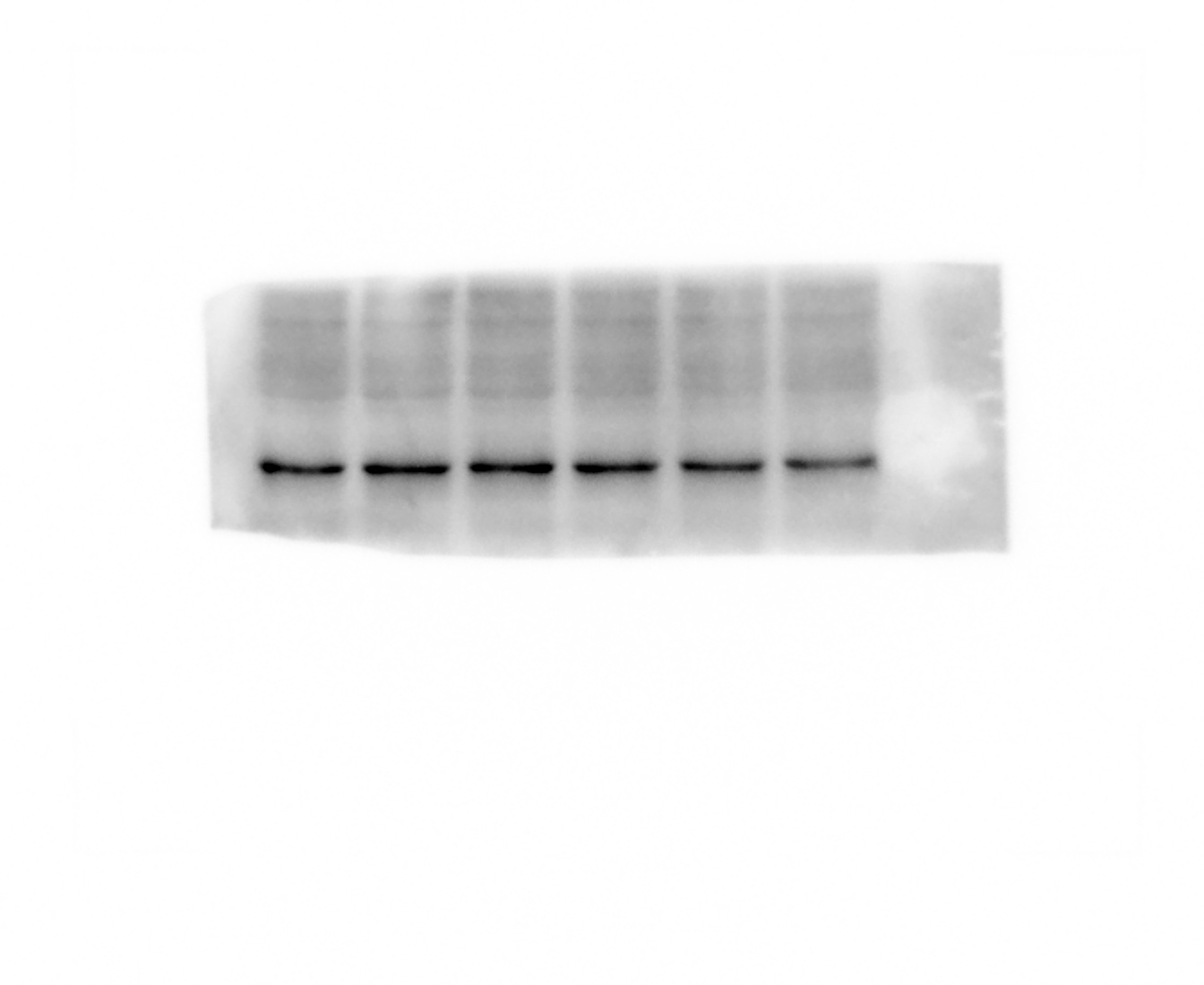

Supplement: Supplementary file 1 [file Data_Sheet_1.ZIP › data/Cx26 in A431 cells and HaCaT cells/A cx26.jpg]

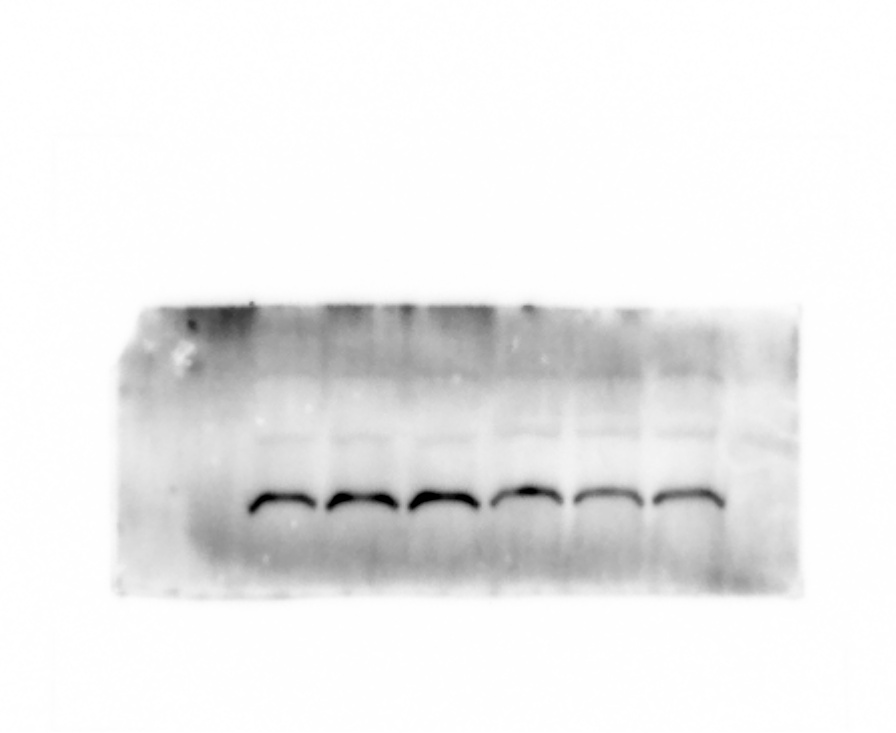

Supplement: Supplementary file 1 [file Data_Sheet_1.ZIP › data/Cx26 in A431 cells and HaCaT cells/B Cx26.jpg]

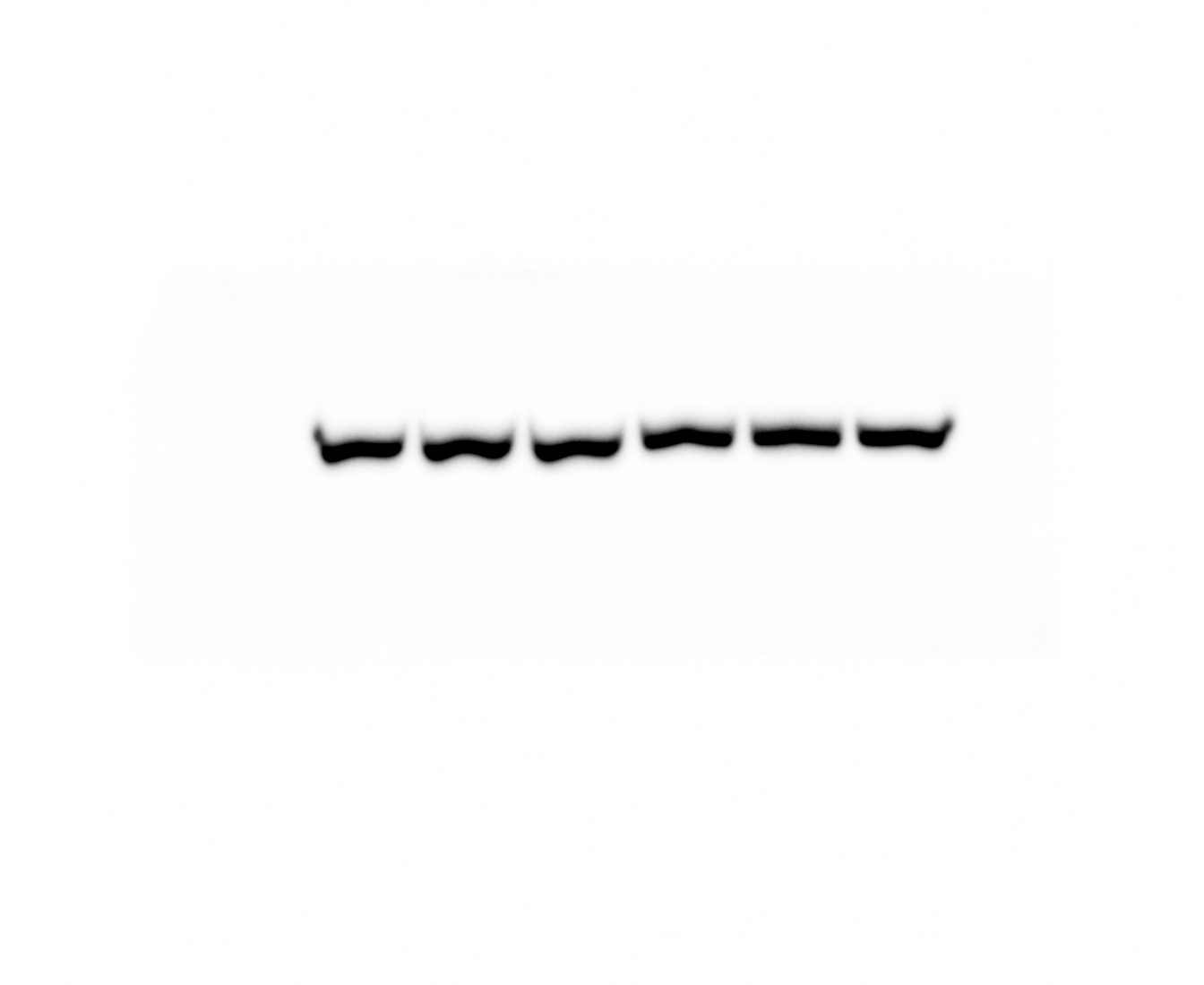

Supplement: Supplementary file 1 [file Data_Sheet_1.ZIP › data/Cx26 in A431 cells and HaCaT cells/B actin.jpg]

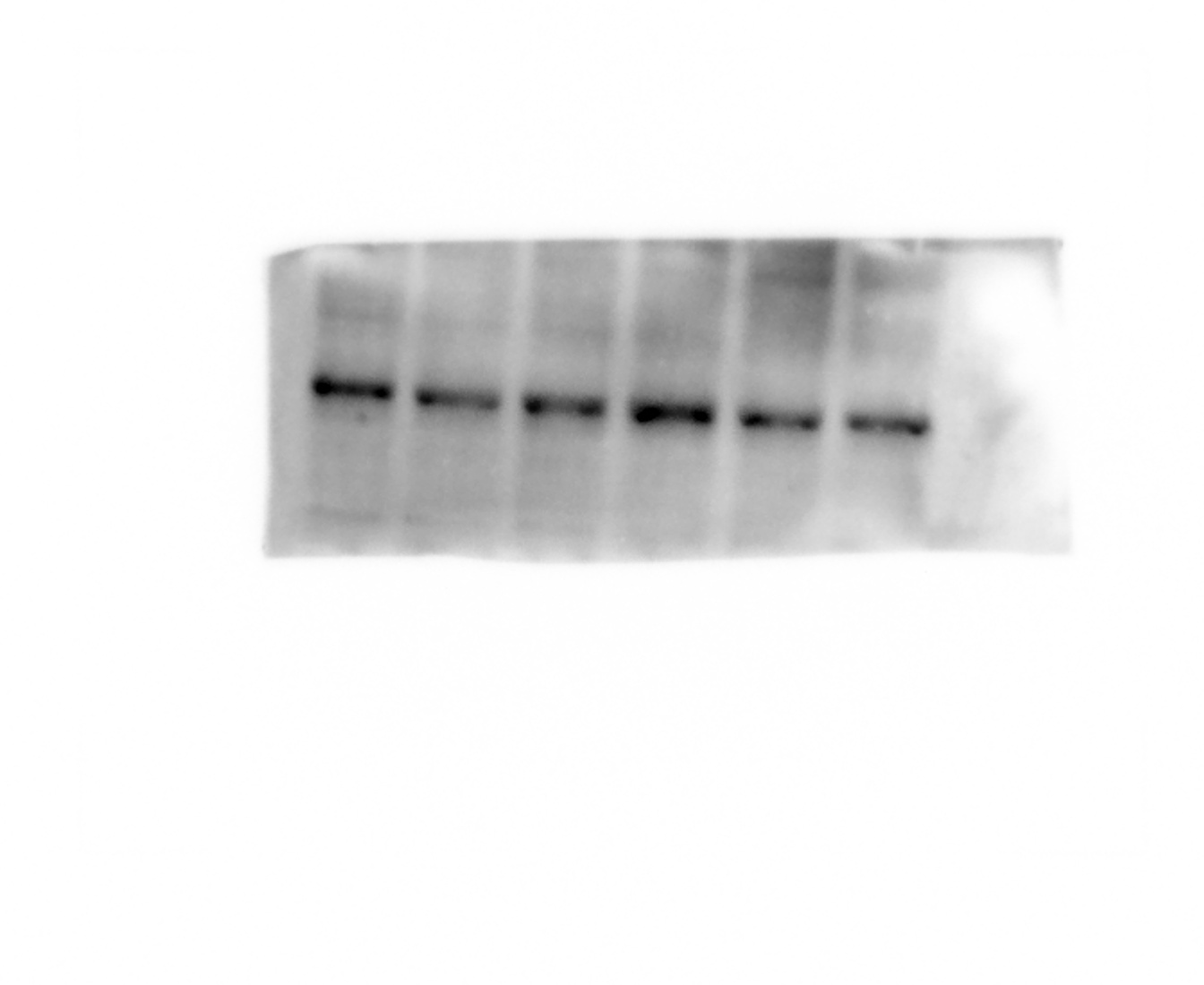

Supplement: Supplementary file 1 [file Data_Sheet_1.ZIP › data/Cx26 in A431 cells and HaCaT cells/C Cx26.jpg]

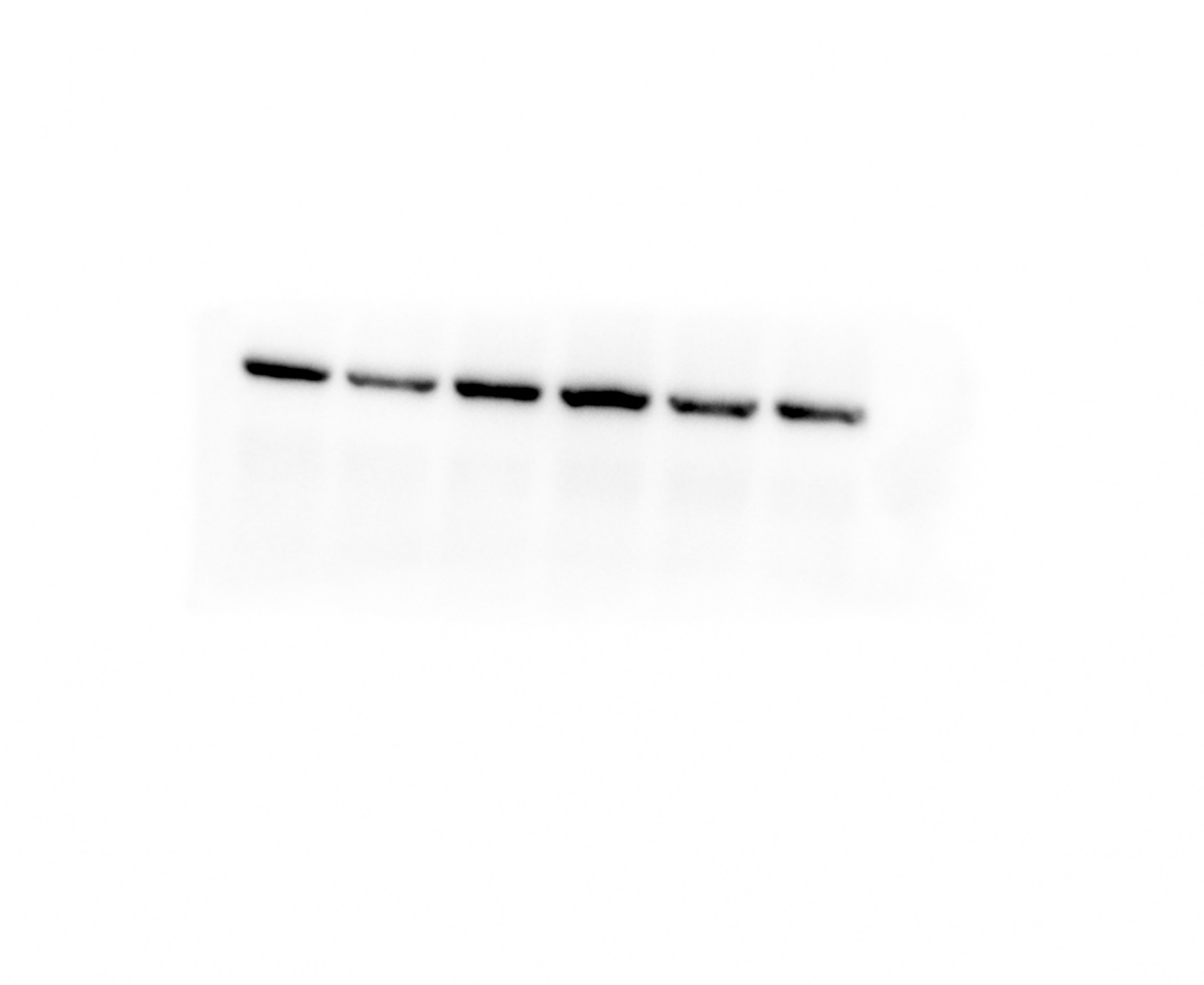

Supplement: Supplementary file 1 [file Data_Sheet_1.ZIP › data/Cx26 in A431 cells and HaCaT cells/C actin.jpg]

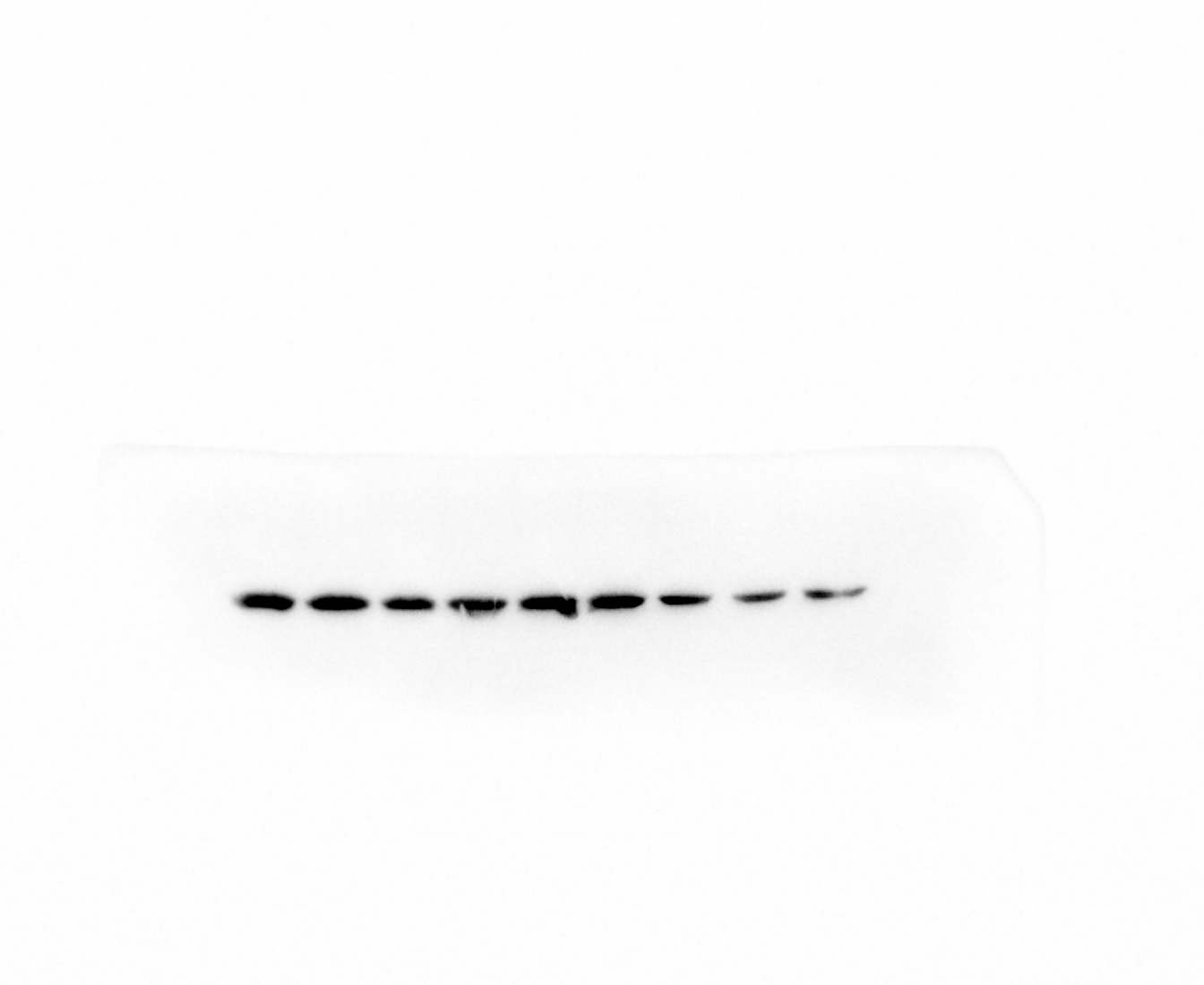

Supplement: Supplementary file 1 [file Data_Sheet_1.ZIP › data/Knockout identification of Cx26/actin.jpg]

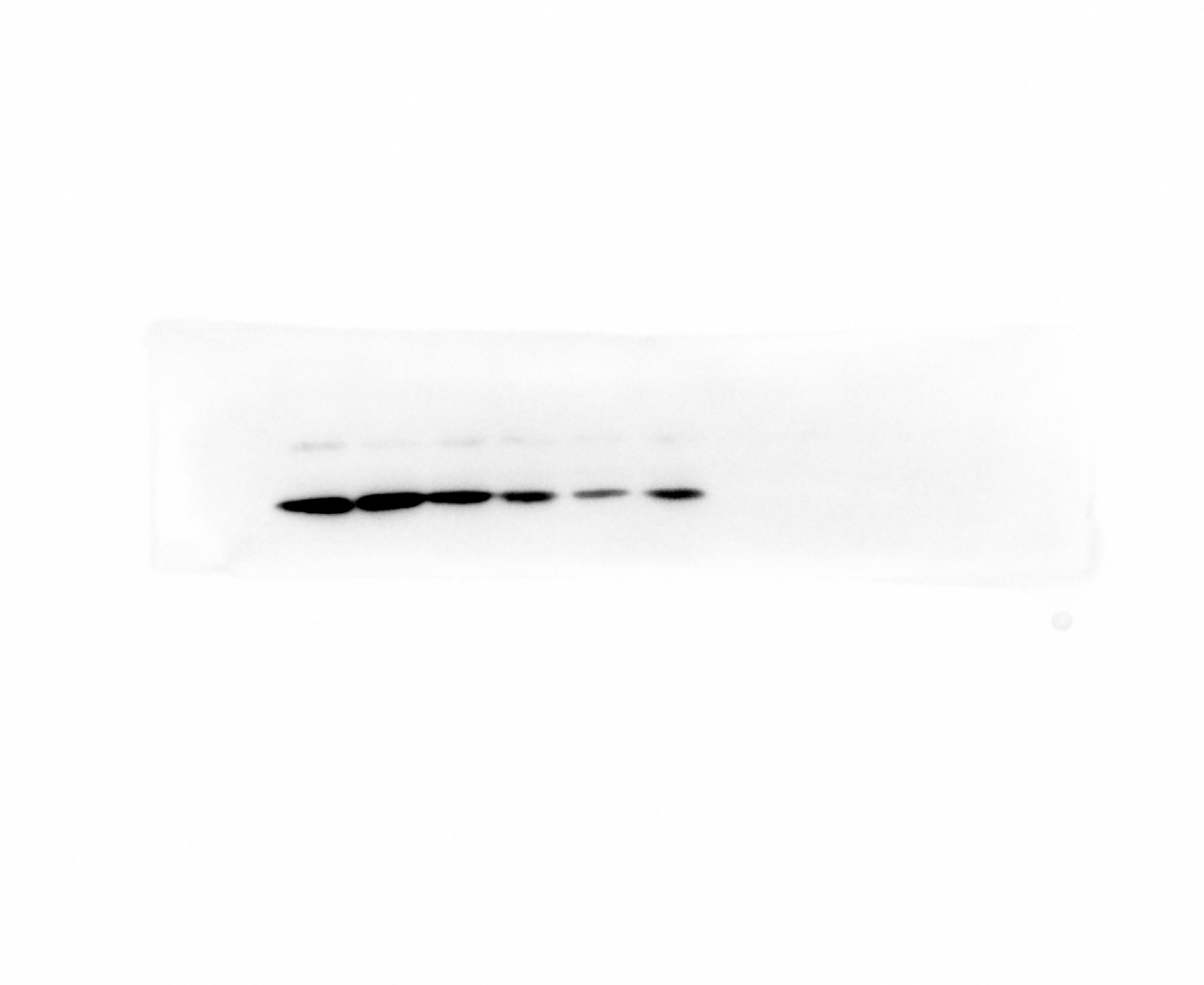

Supplement: Supplementary file 1 [file Data_Sheet_1.ZIP › data/Knockout identification of Cx26/cx26.jpg]

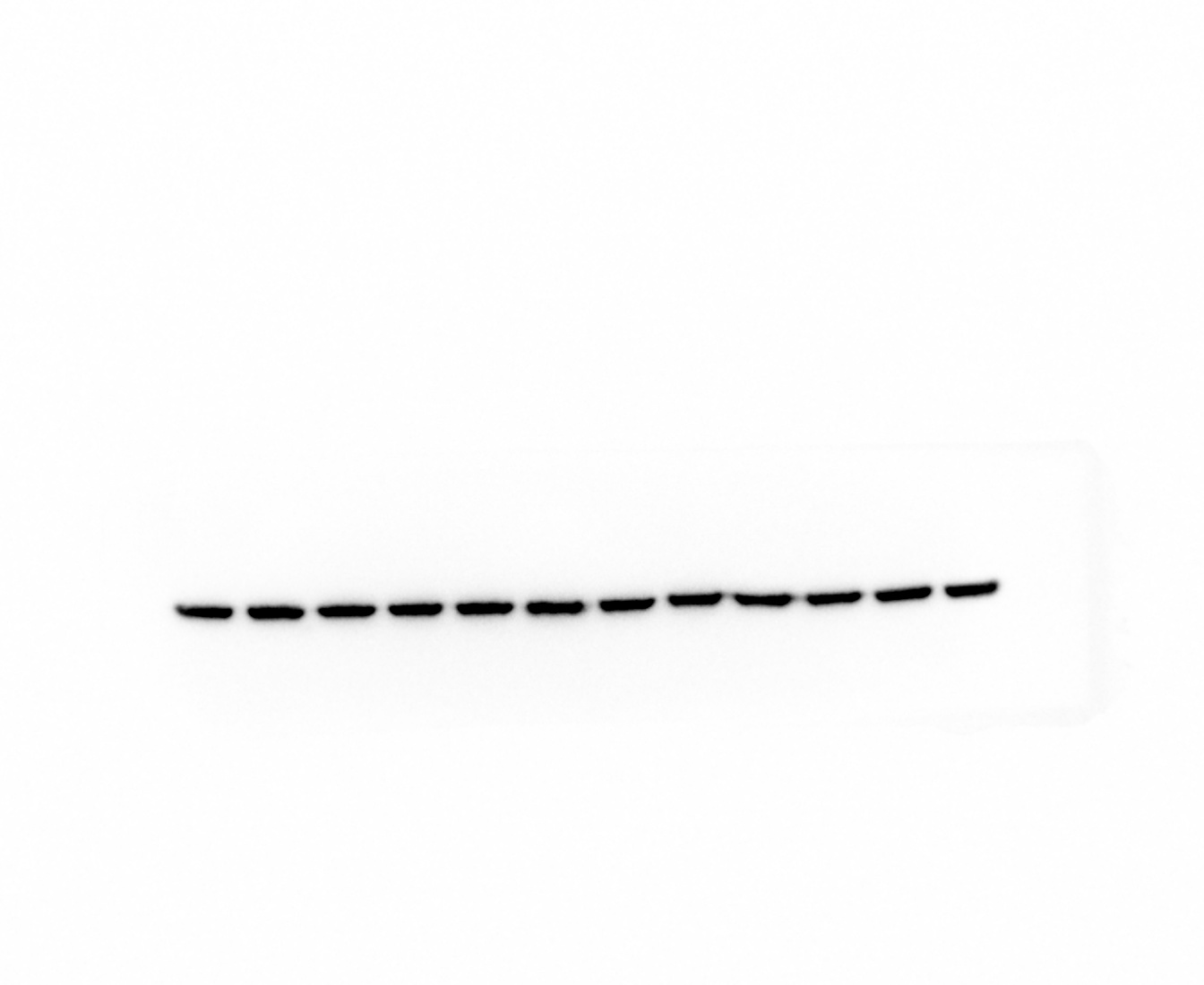

Supplement: Supplementary file 1 [file Data_Sheet_1.ZIP › data/WB p-ERK/A actin.jpg]

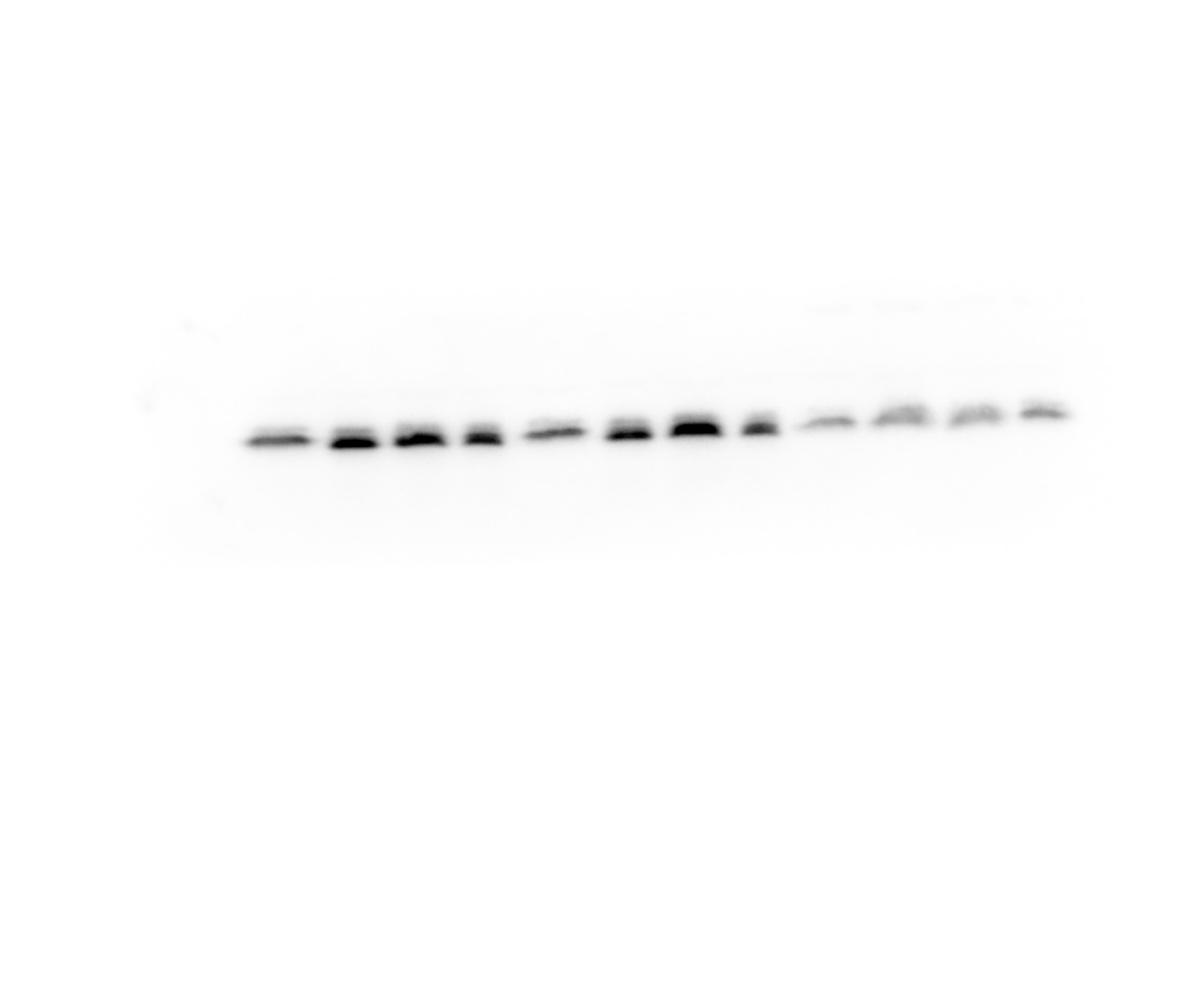

Supplement: Supplementary file 1 [file Data_Sheet_1.ZIP › data/WB p-ERK/A ERK.jpg]

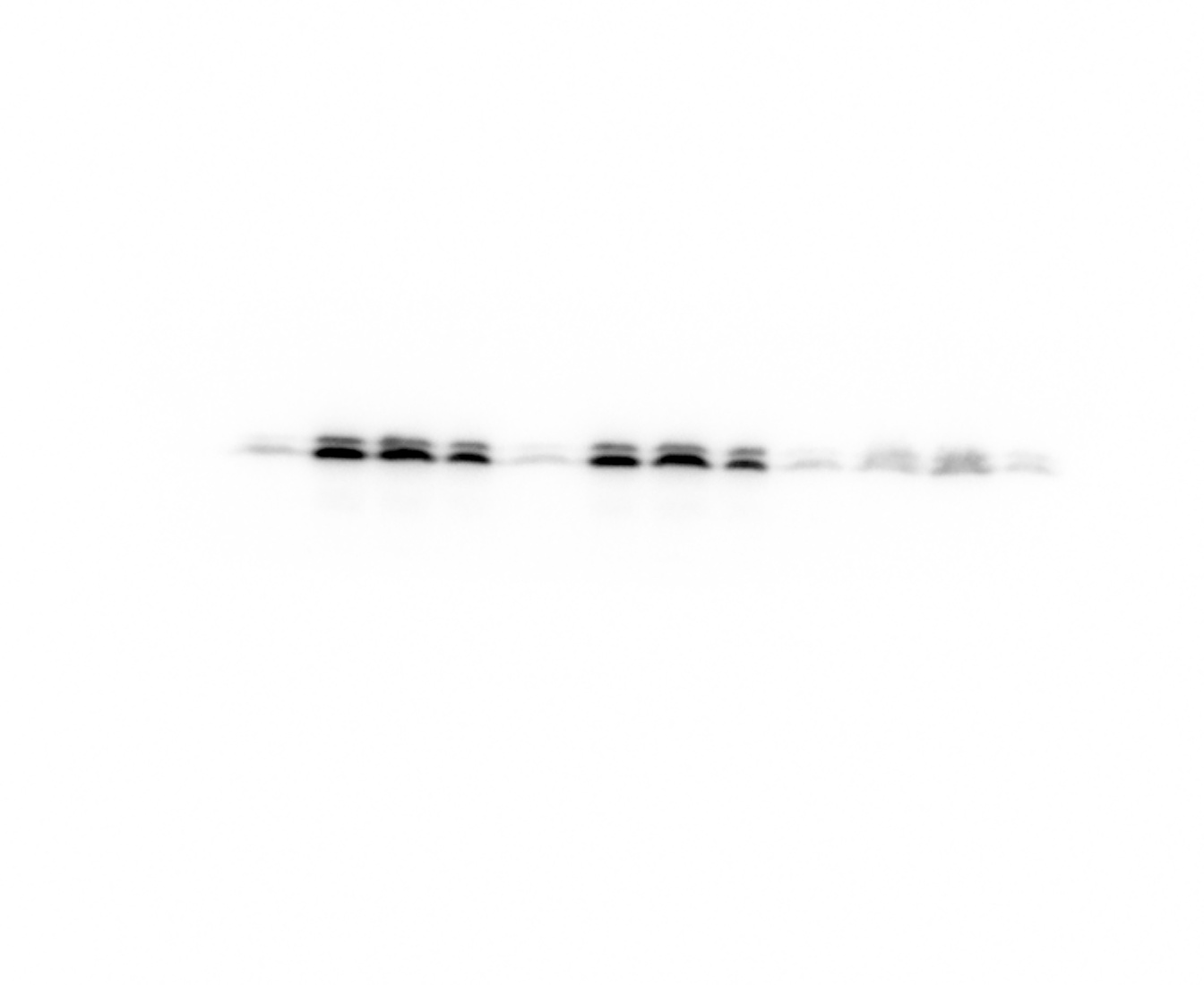

Supplement: Supplementary file 1 [file Data_Sheet_1.ZIP › data/WB p-ERK/A p-ERK.jpg]

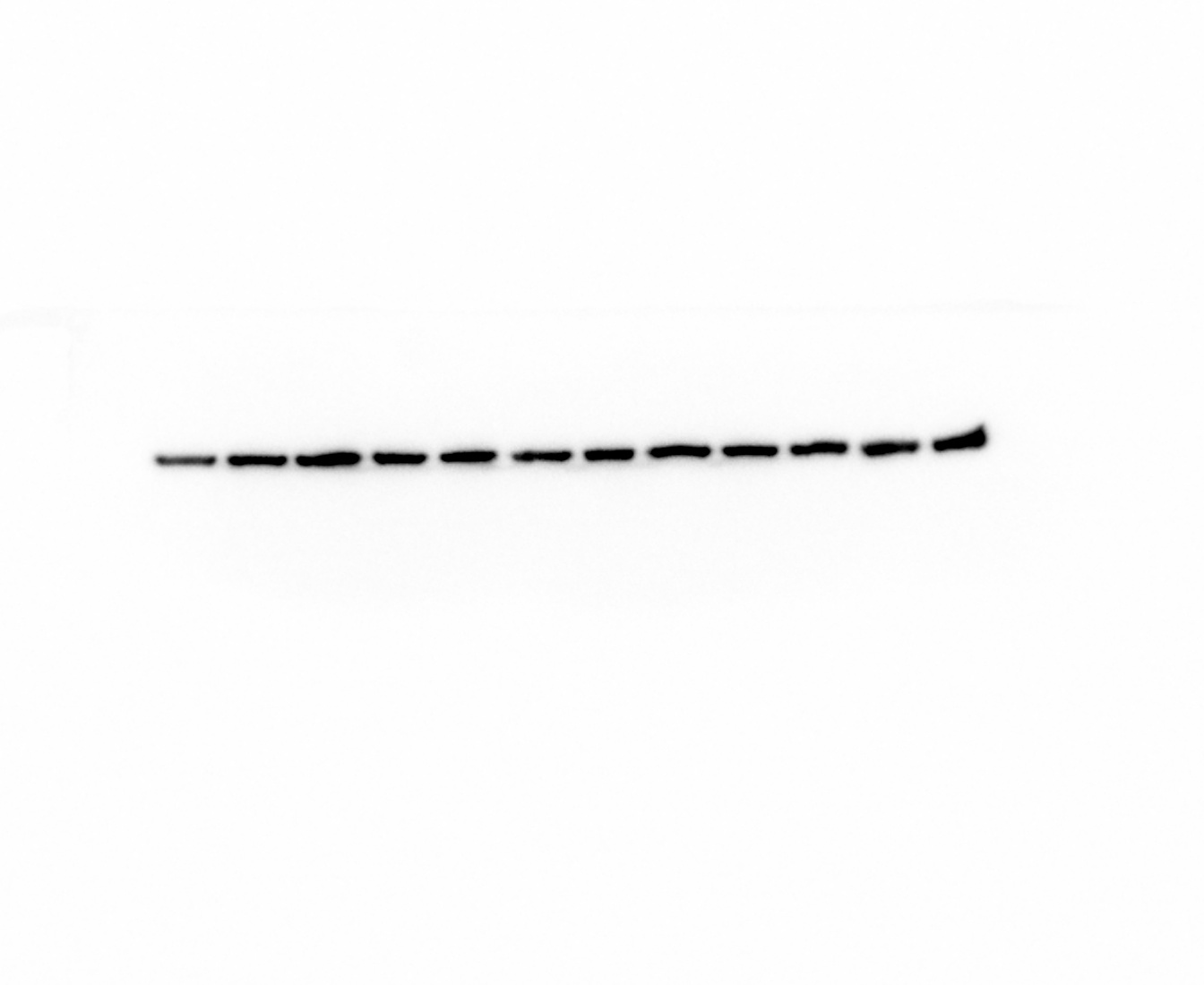

Supplement: Supplementary file 1 [file Data_Sheet_1.ZIP › data/WB p-ERK/B Actin.jpg]

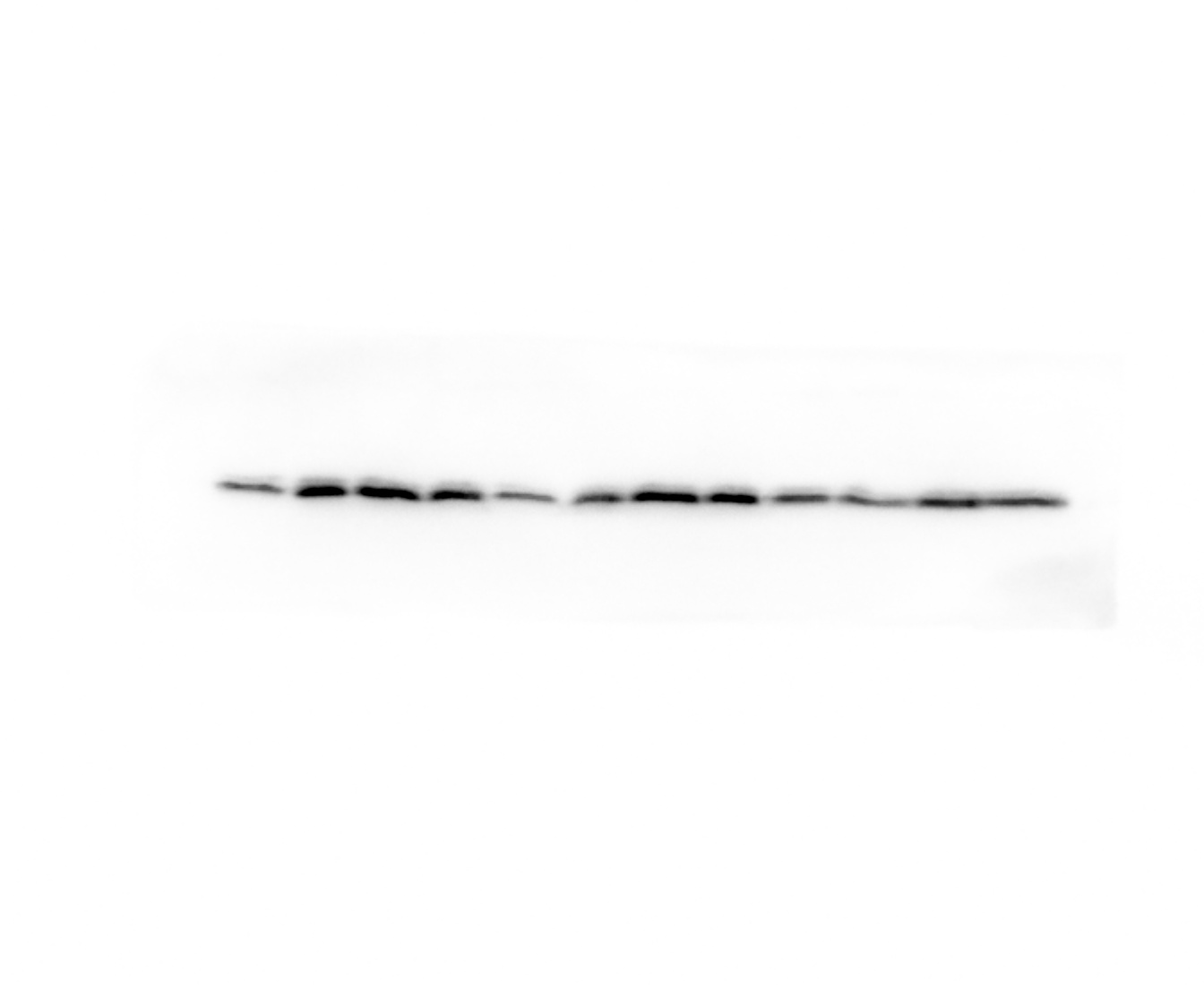

Supplement: Supplementary file 1 [file Data_Sheet_1.ZIP › data/WB p-ERK/B ERK.jpg]

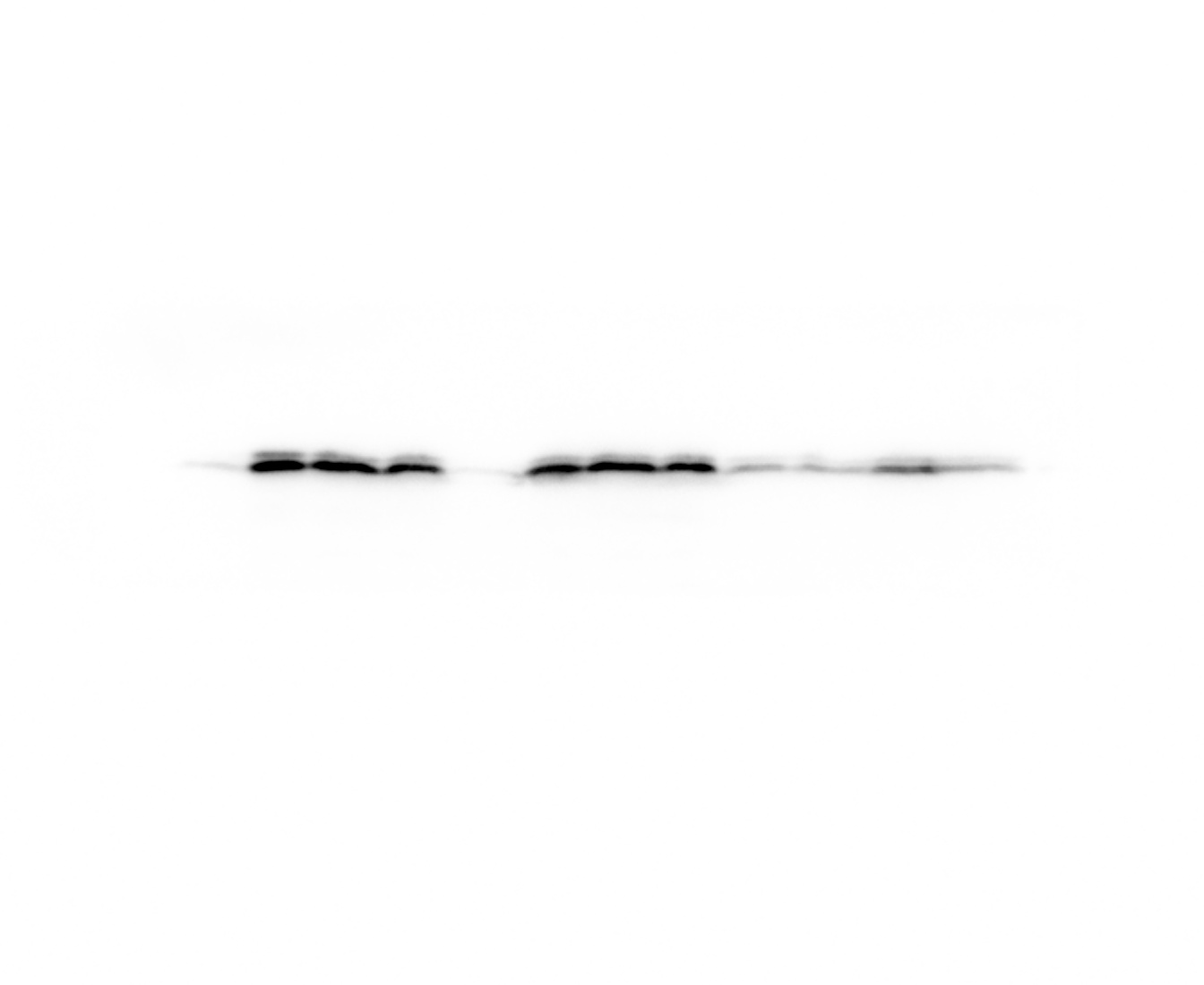

Supplement: Supplementary file 1 [file Data_Sheet_1.ZIP › data/WB p-ERK/B p-ERK.jpg]

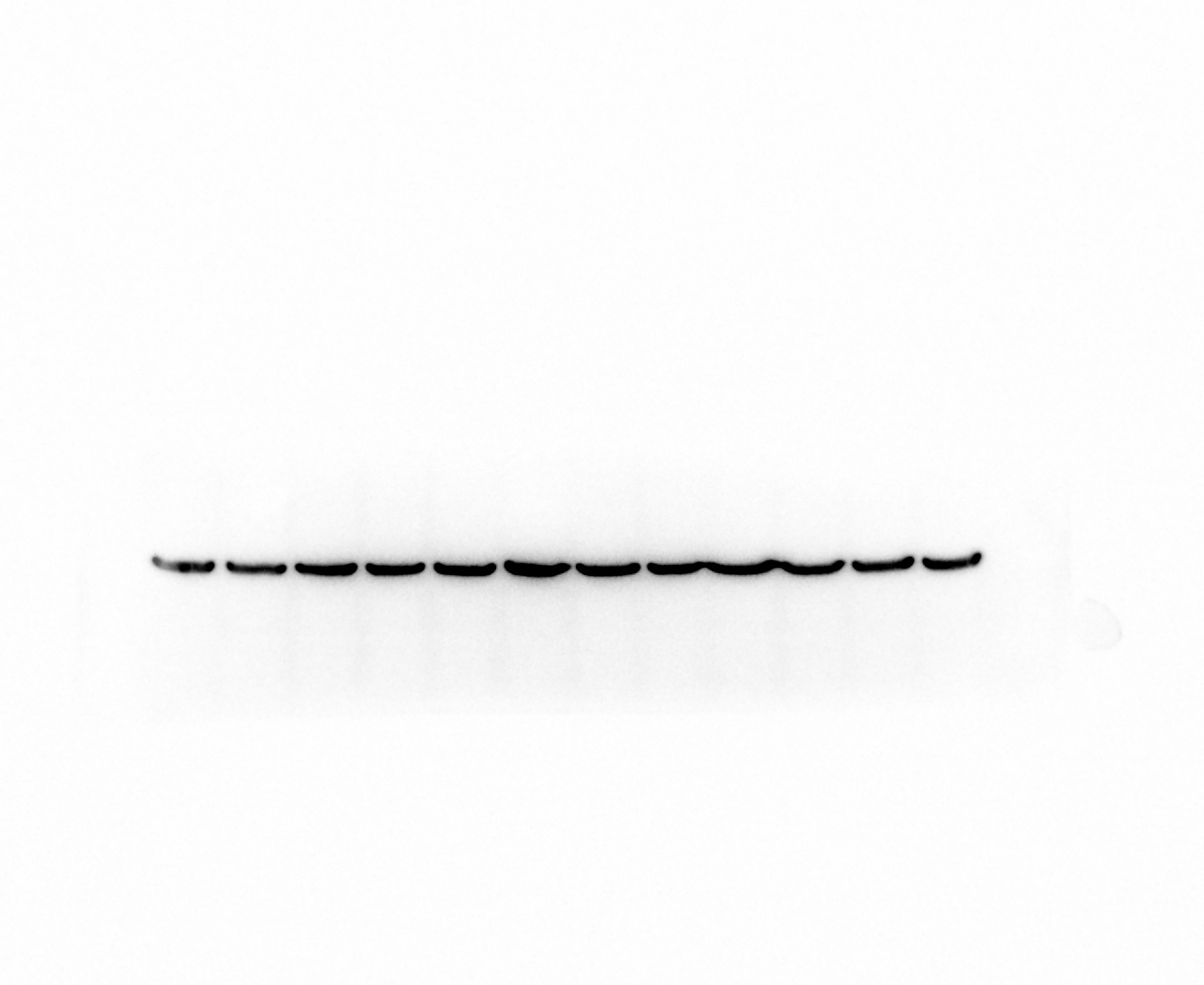

Supplement: Supplementary file 1 [file Data_Sheet_1.ZIP › data/WB p-ERK/C Actin.jpg]

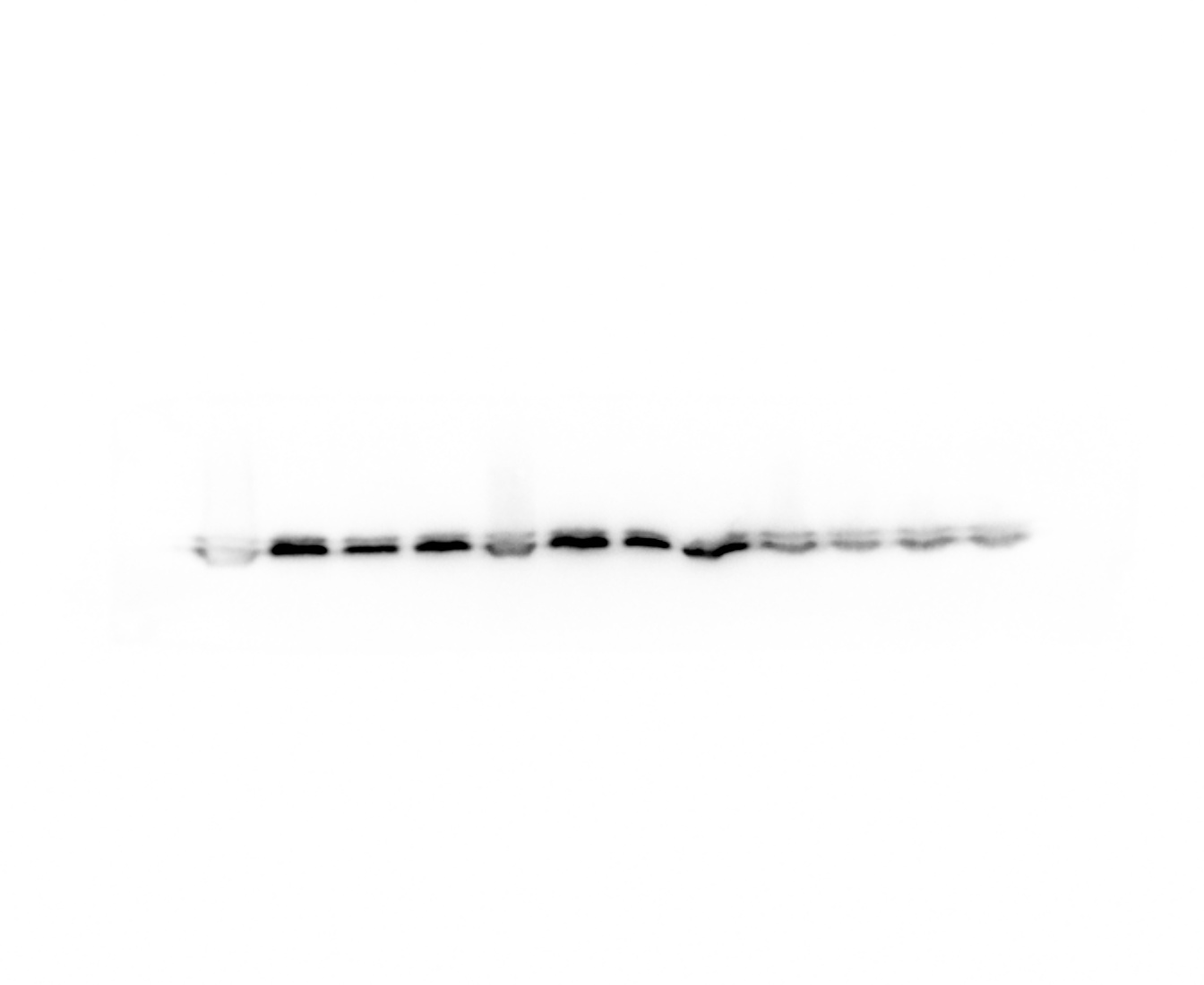

Supplement: Supplementary file 1 [file Data_Sheet_1.ZIP › data/WB p-ERK/C ERK.jpg]

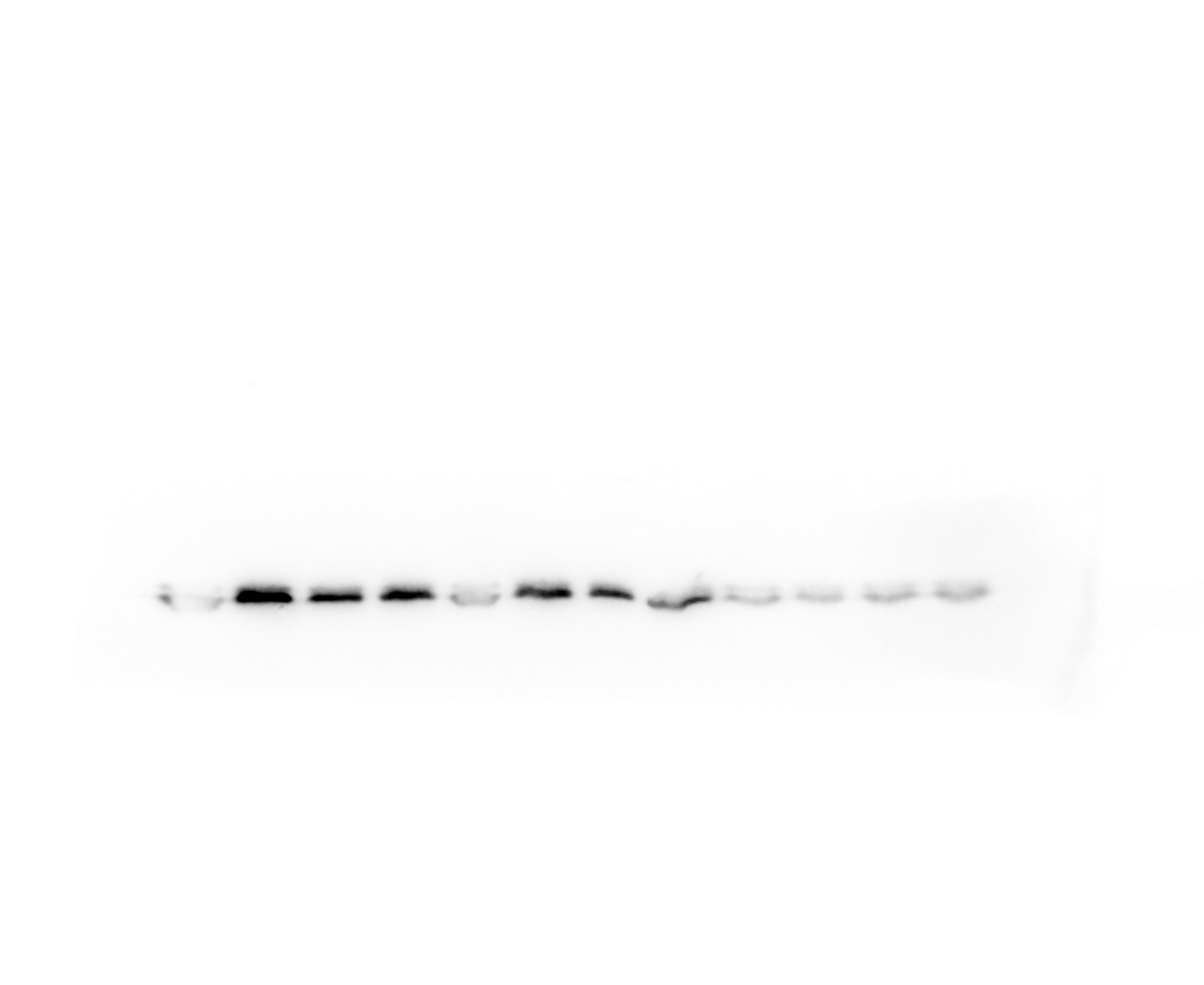

Supplement: Supplementary file 1 [file Data_Sheet_1.ZIP › data/WB p-ERK/C p-ERK.jpg]

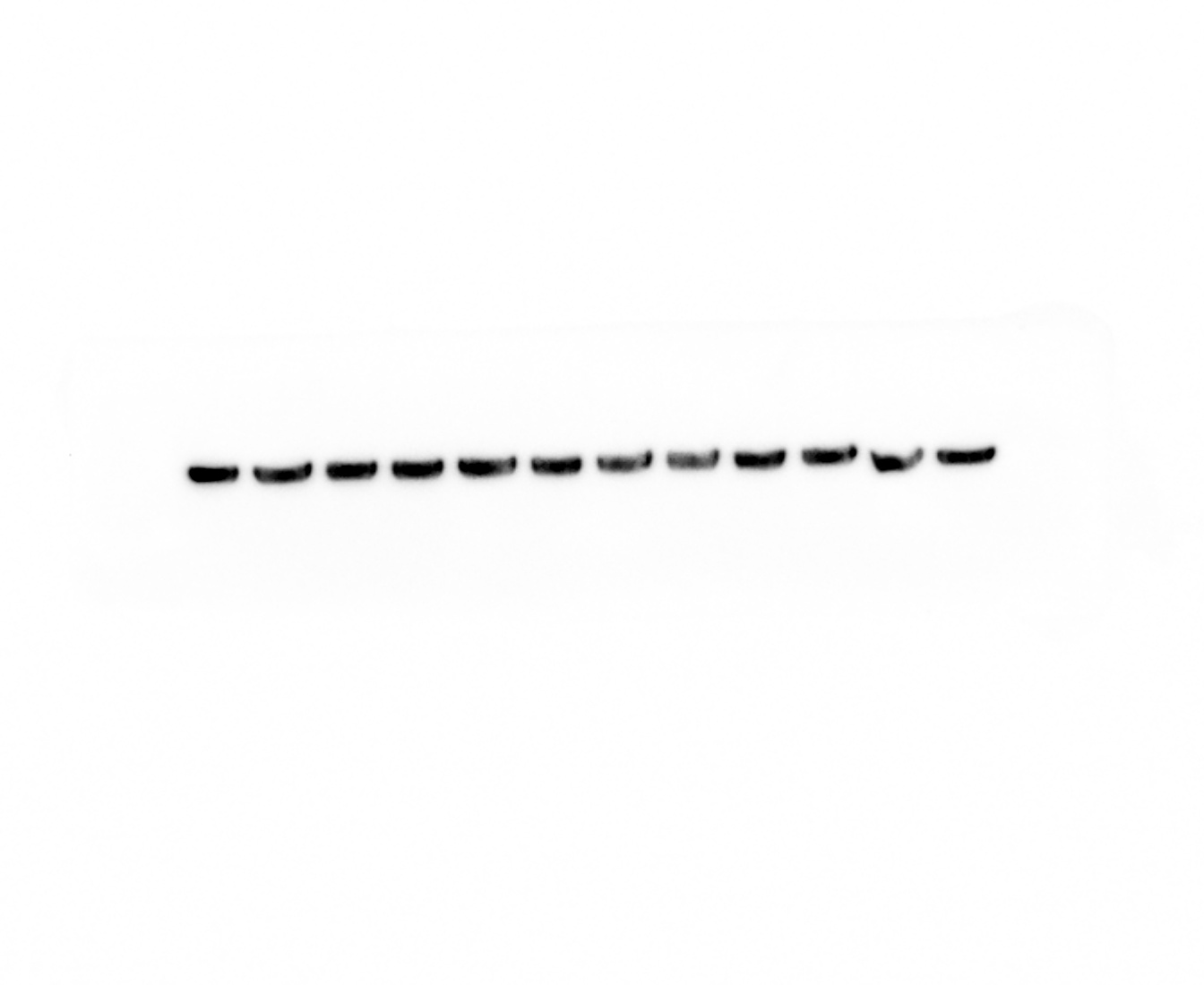

Supplement: Supplementary file 1 [file Data_Sheet_1.ZIP › data/WB p-p38/A Actin.jpg]

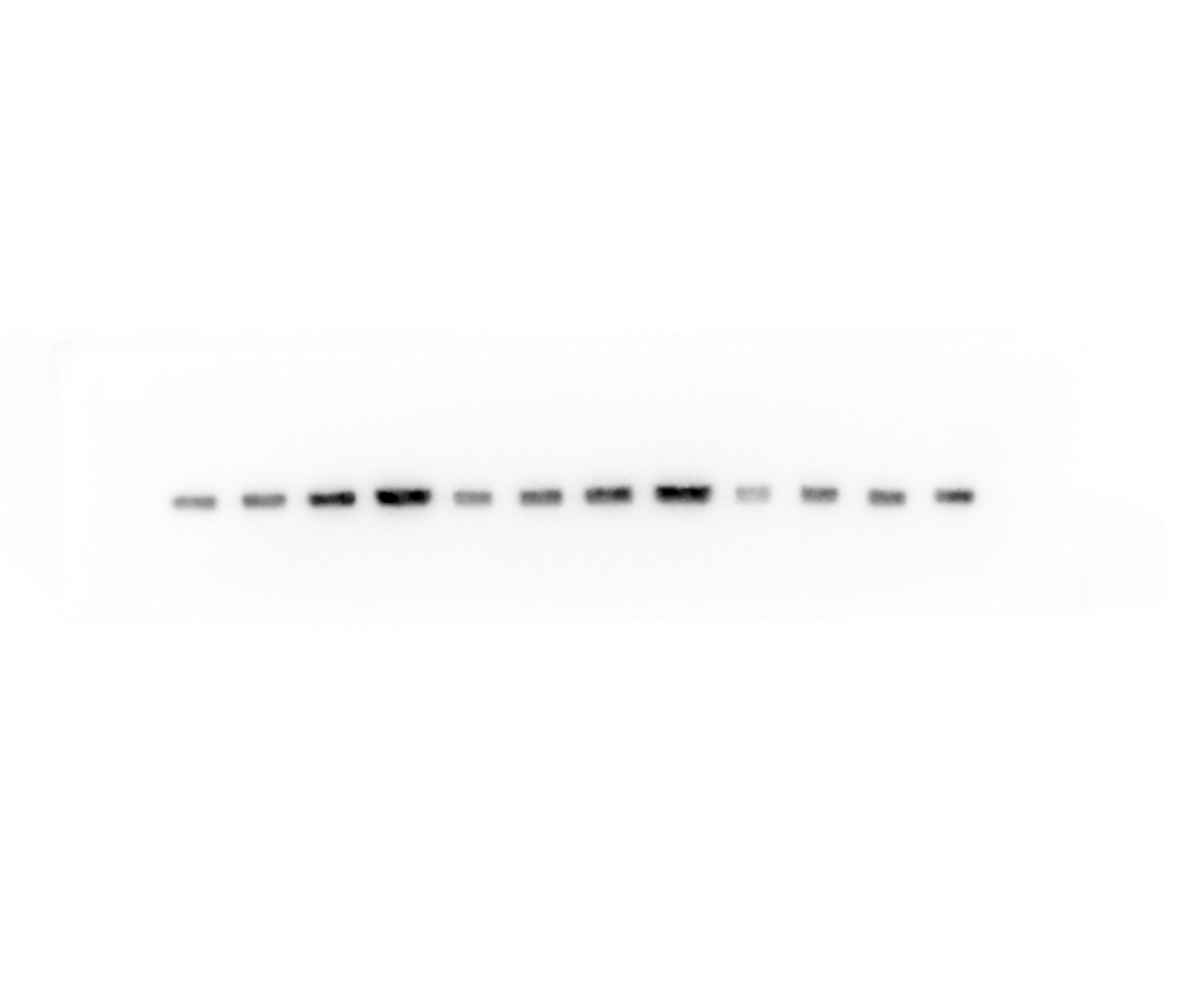

Supplement: Supplementary file 1 [file Data_Sheet_1.ZIP › data/WB p-p38/A p-p38.jpg]

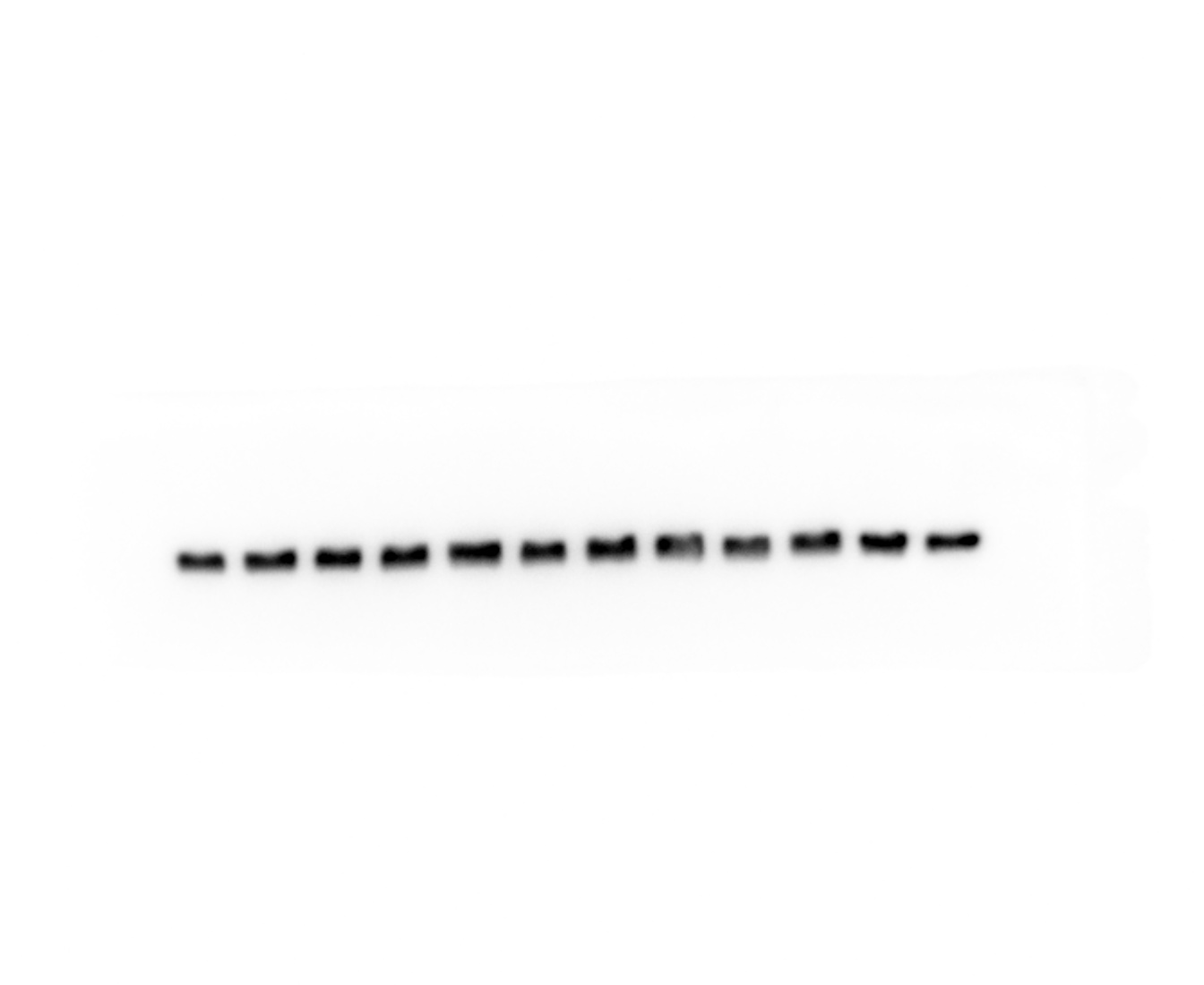

Supplement: Supplementary file 1 [file Data_Sheet_1.ZIP › data/WB p-p38/A p38.jpg]

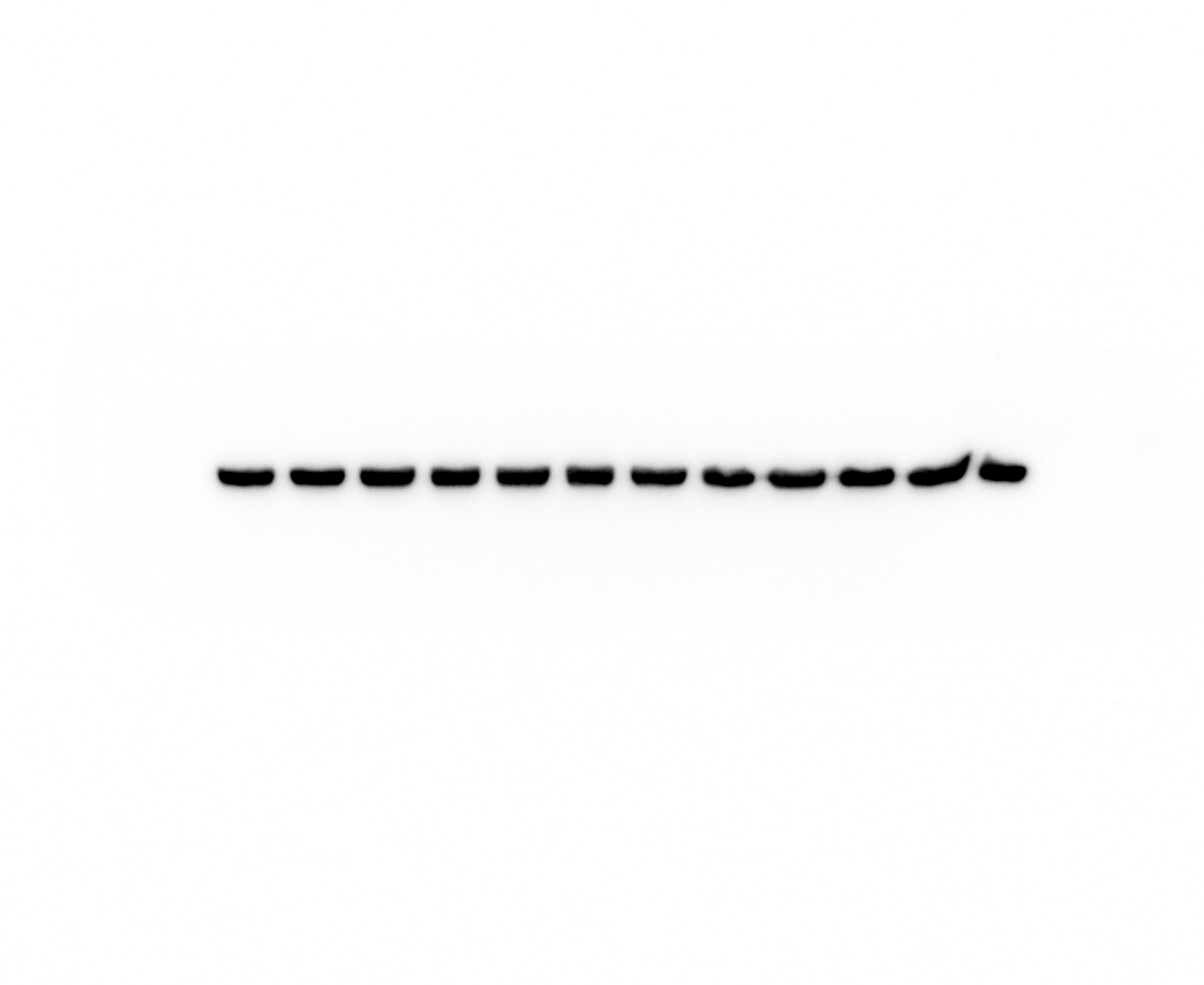

Supplement: Supplementary file 1 [file Data_Sheet_1.ZIP › data/WB p-p38/B Actin.jpg]

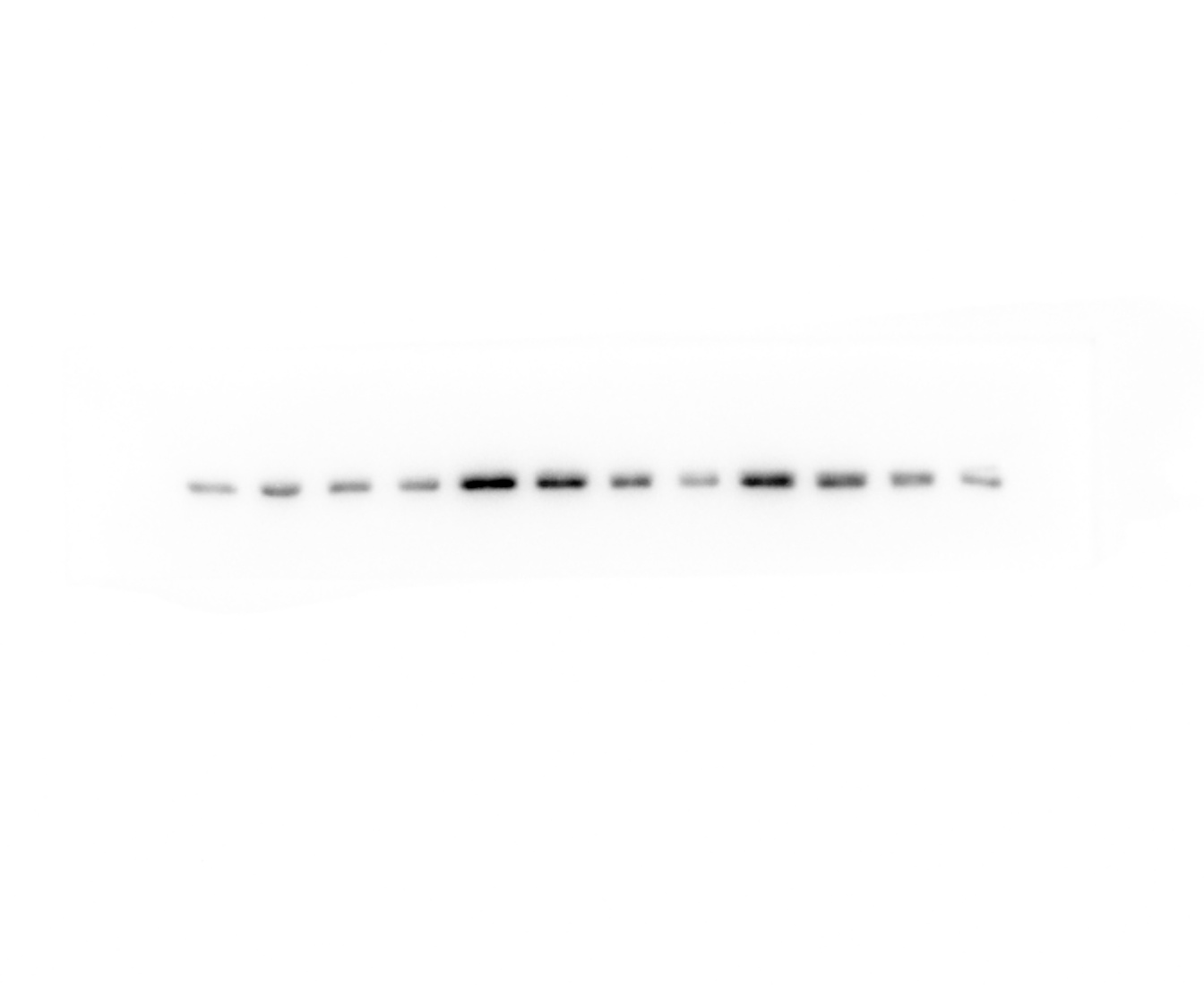

Supplement: Supplementary file 1 [file Data_Sheet_1.ZIP › data/WB p-p38/B p-p38.jpg]

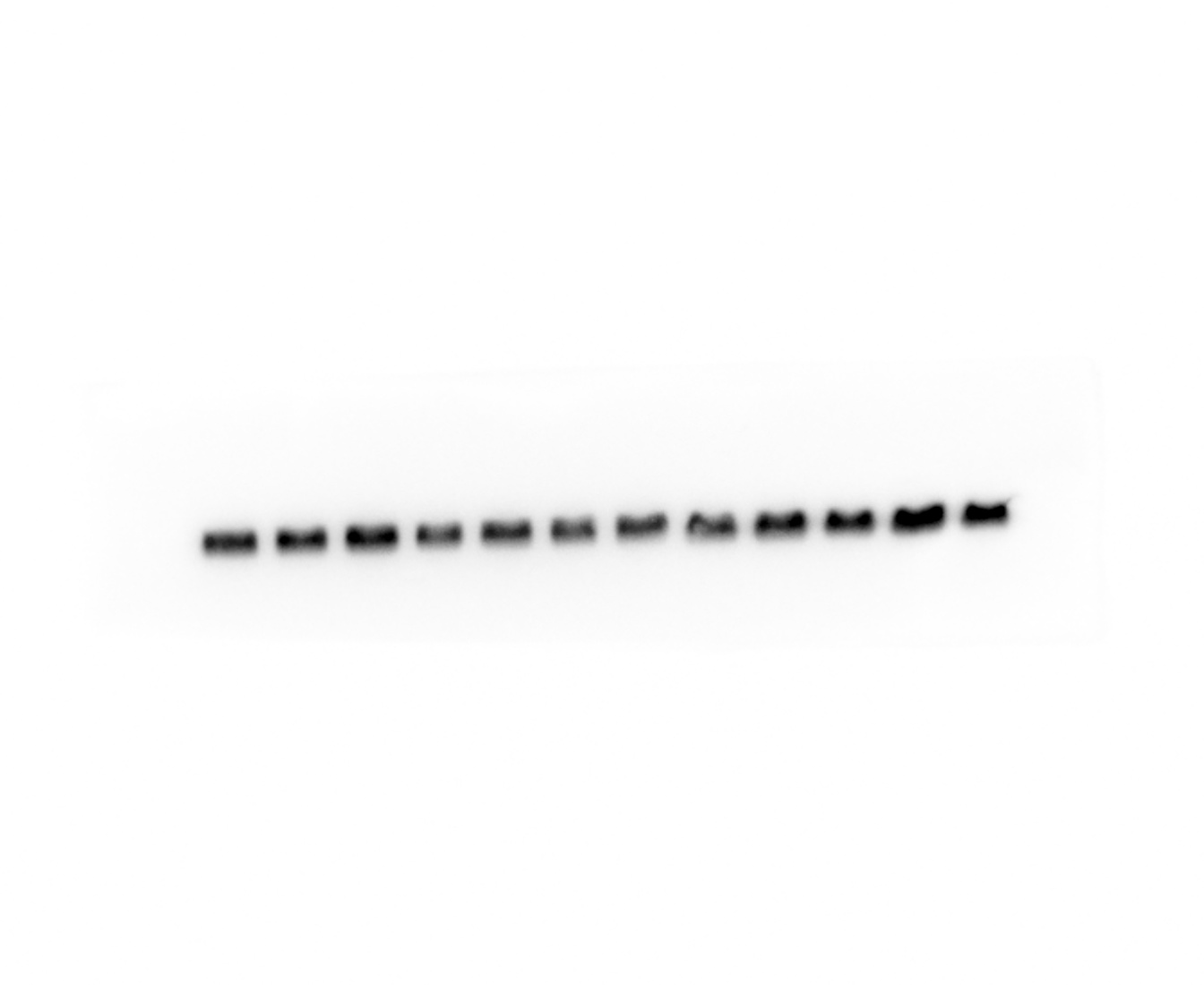

Supplement: Supplementary file 1 [file Data_Sheet_1.ZIP › data/WB p-p38/B p38.jpg]

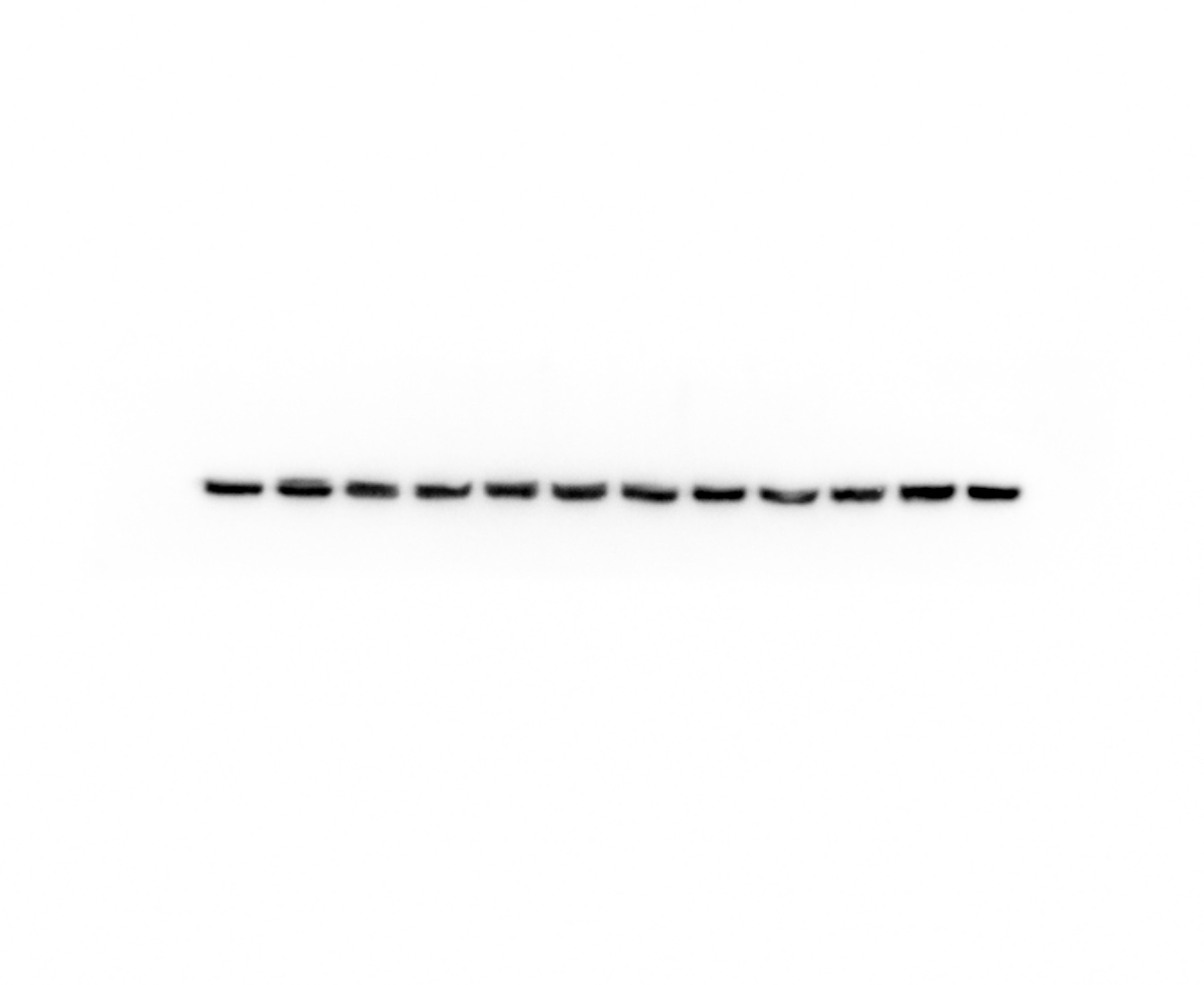

Supplement: Supplementary file 1 [file Data_Sheet_1.ZIP › data/WB p-p38/C Actin.jpg]

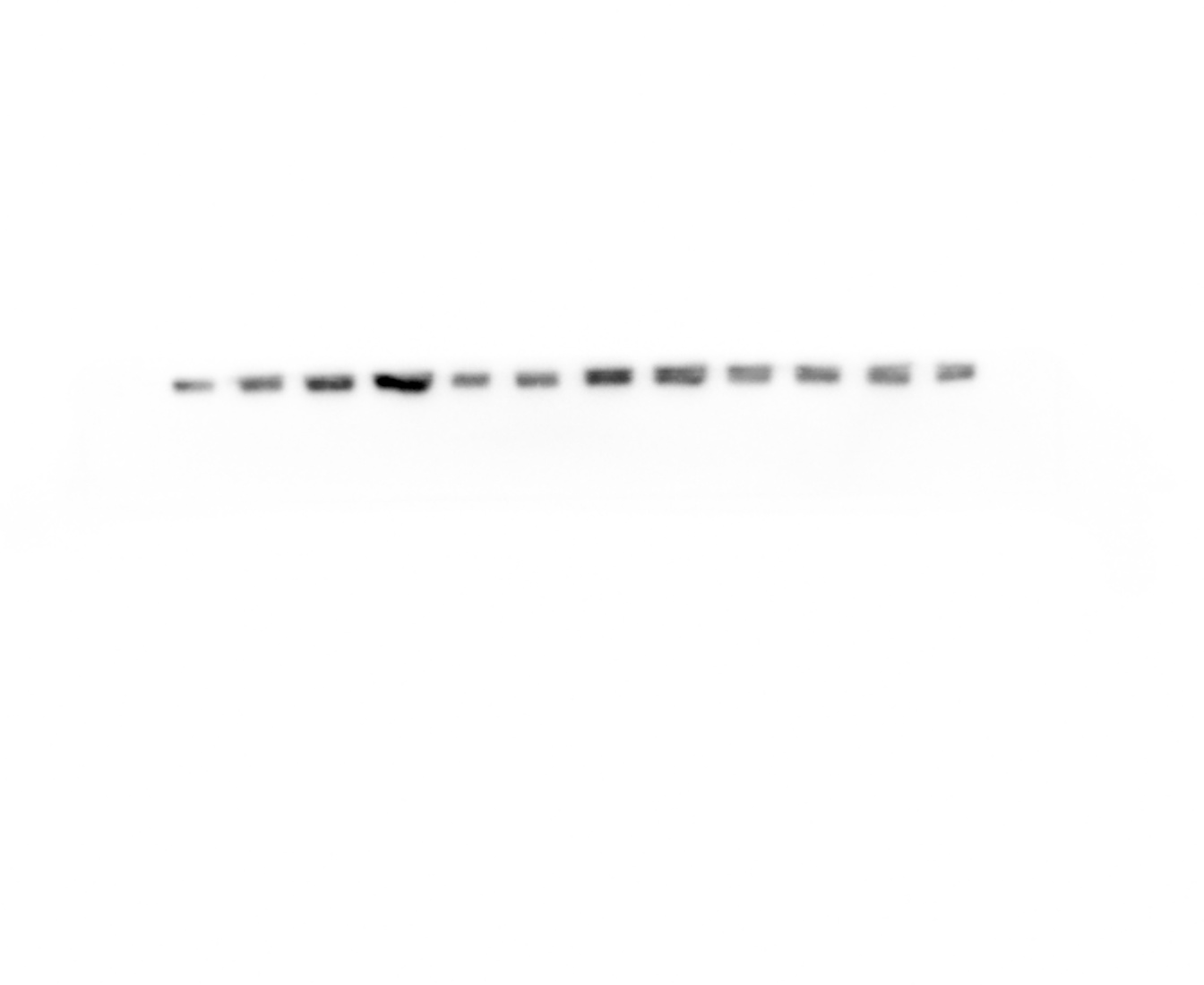

Supplement: Supplementary file 1 [file Data_Sheet_1.ZIP › data/WB p-p38/C p-p38.jpg]

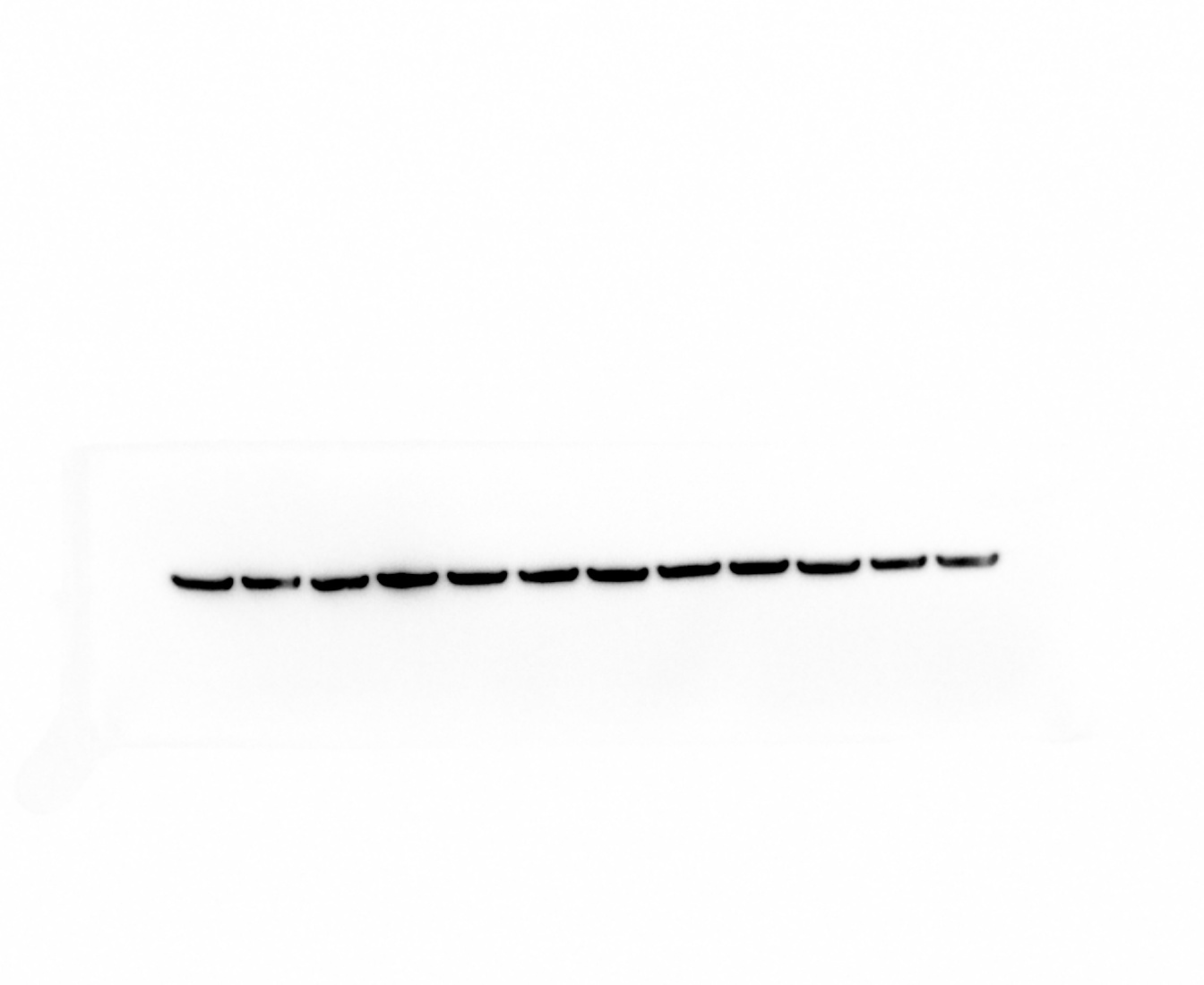

Supplement: Supplementary file 1 [file Data_Sheet_1.ZIP › data/WB p-p38/C p38.jpg]

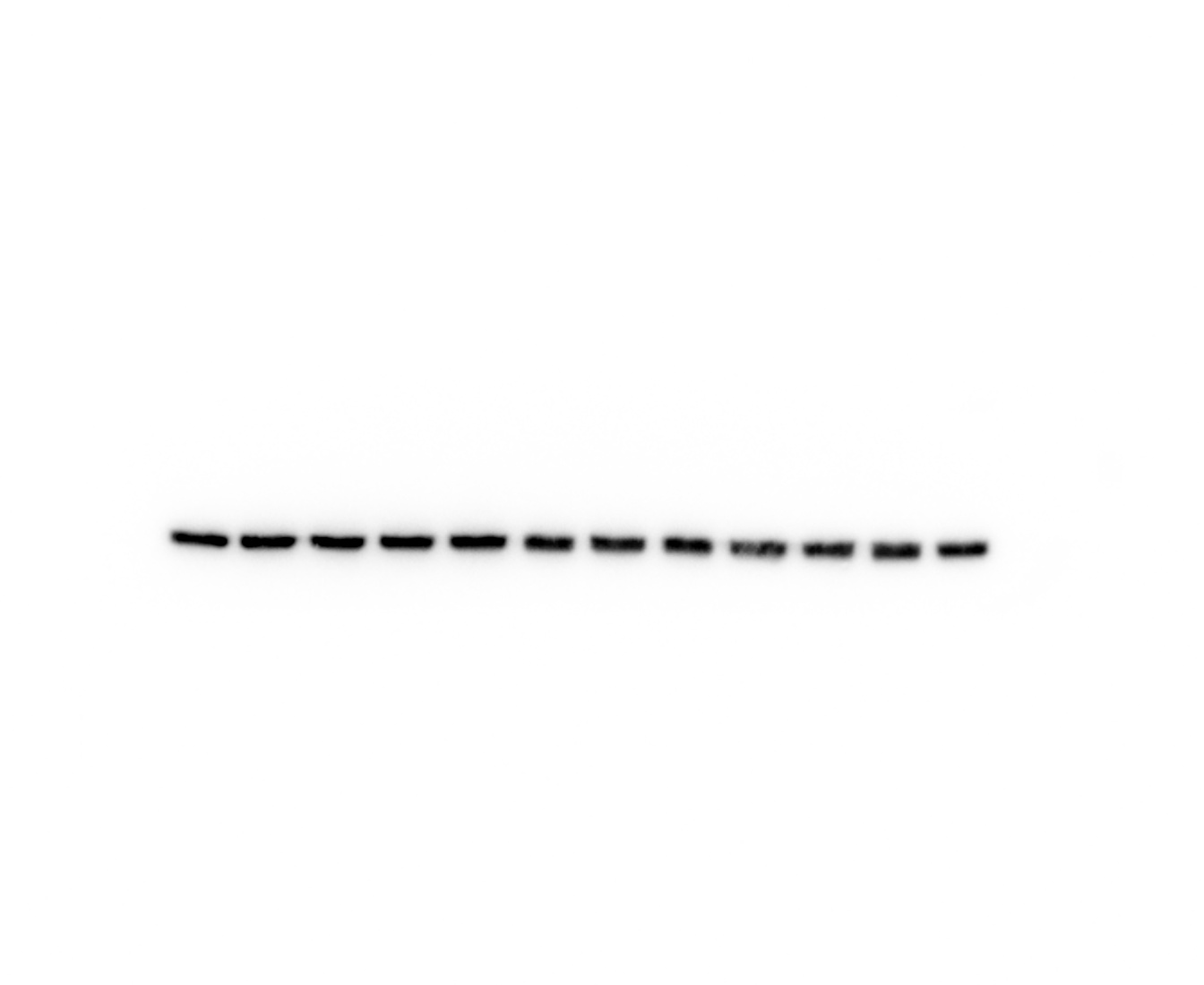

Supplement: Supplementary file 1 [file Data_Sheet_1.ZIP › data/WB p65/A actin.jpg]

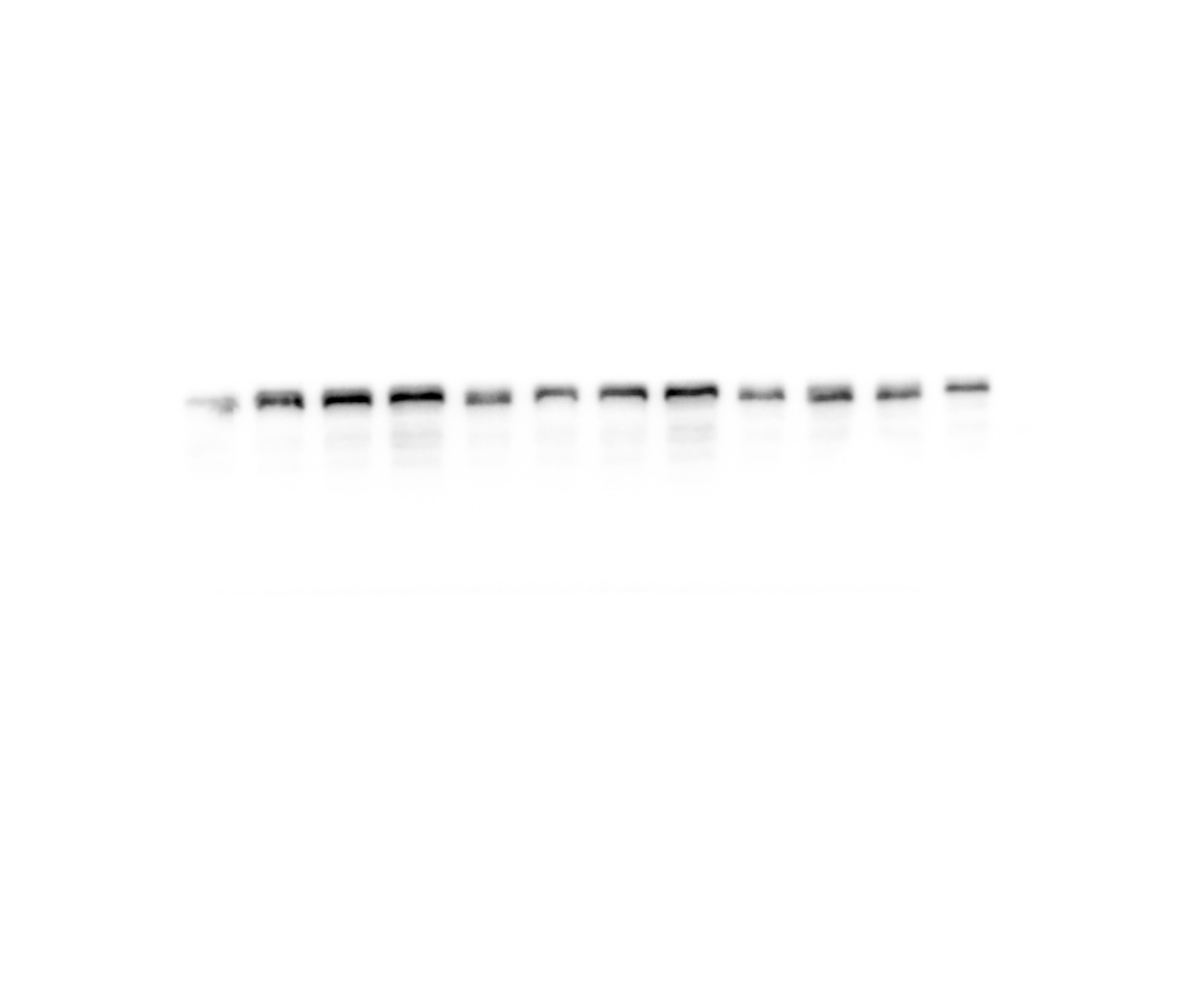

Supplement: Supplementary file 1 [file Data_Sheet_1.ZIP › data/WB p65/A p65.jpg]

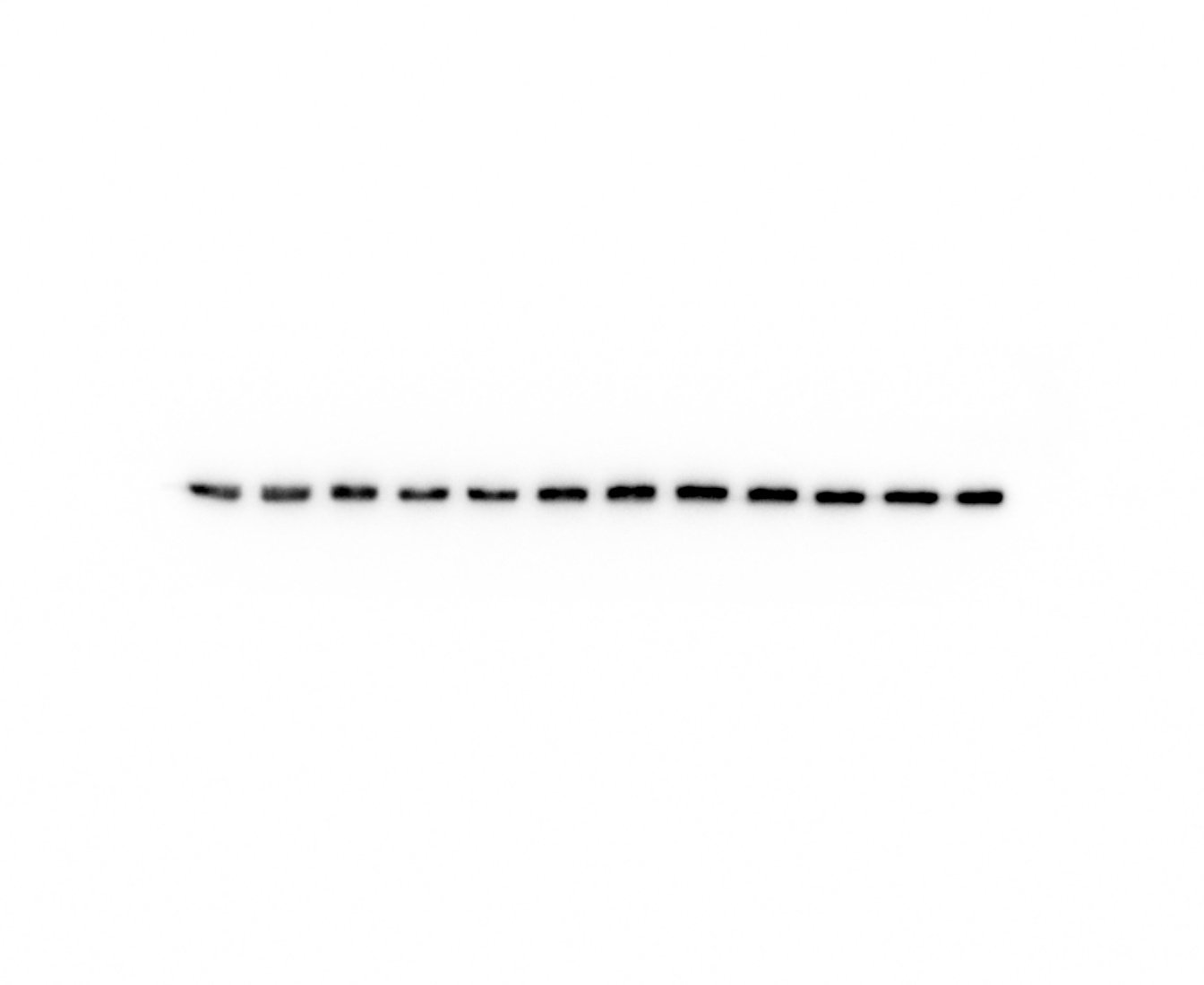

Supplement: Supplementary file 1 [file Data_Sheet_1.ZIP › data/WB p65/B actin.jpg]

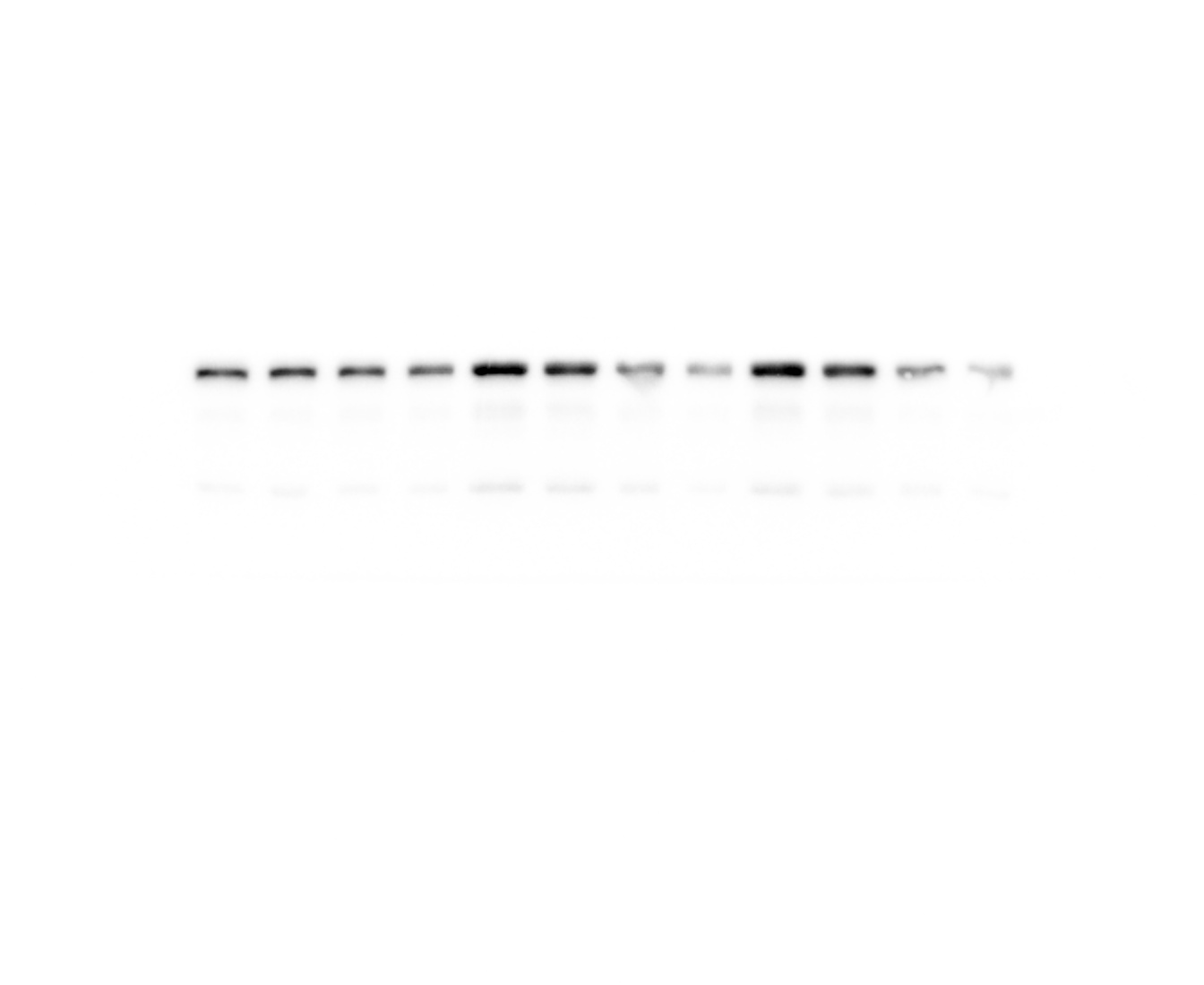

Supplement: Supplementary file 1 [file Data_Sheet_1.ZIP › data/WB p65/B p65.jpg]

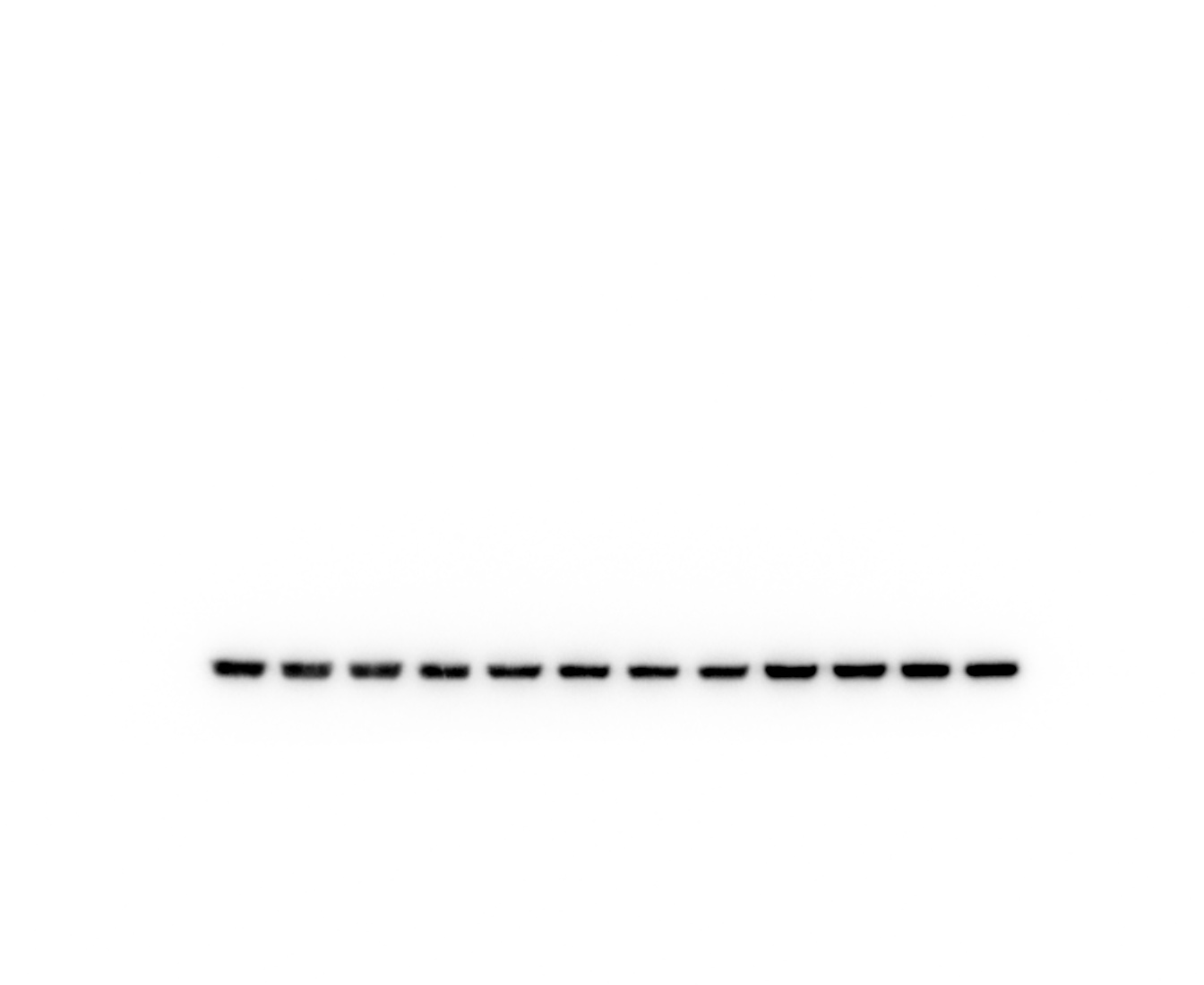

Supplement: Supplementary file 1 [file Data_Sheet_1.ZIP › data/WB p65/C actin.jpg]

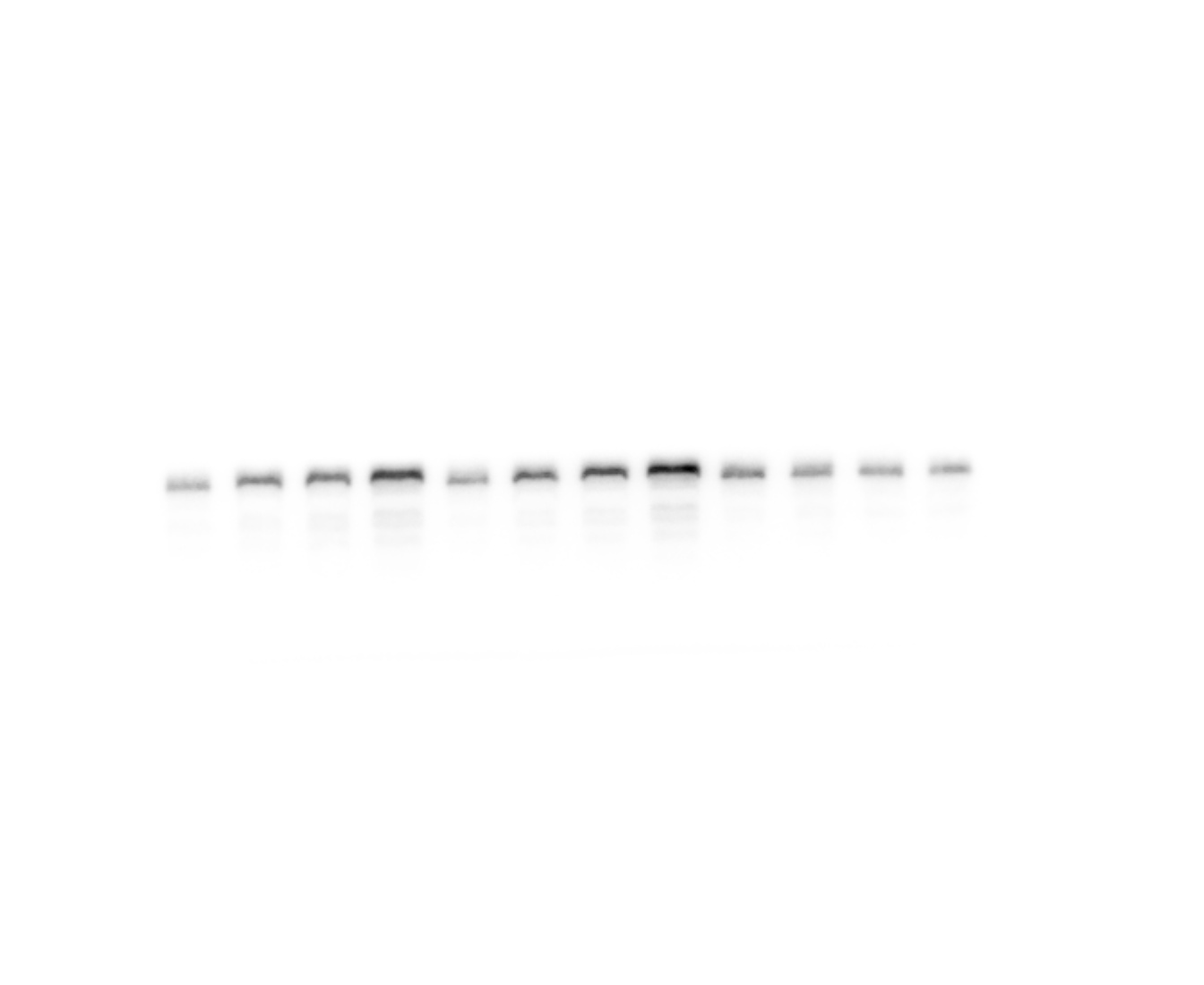

Supplement: Supplementary file 1 [file Data_Sheet_1.ZIP › data/WB p65/C p65.jpg]

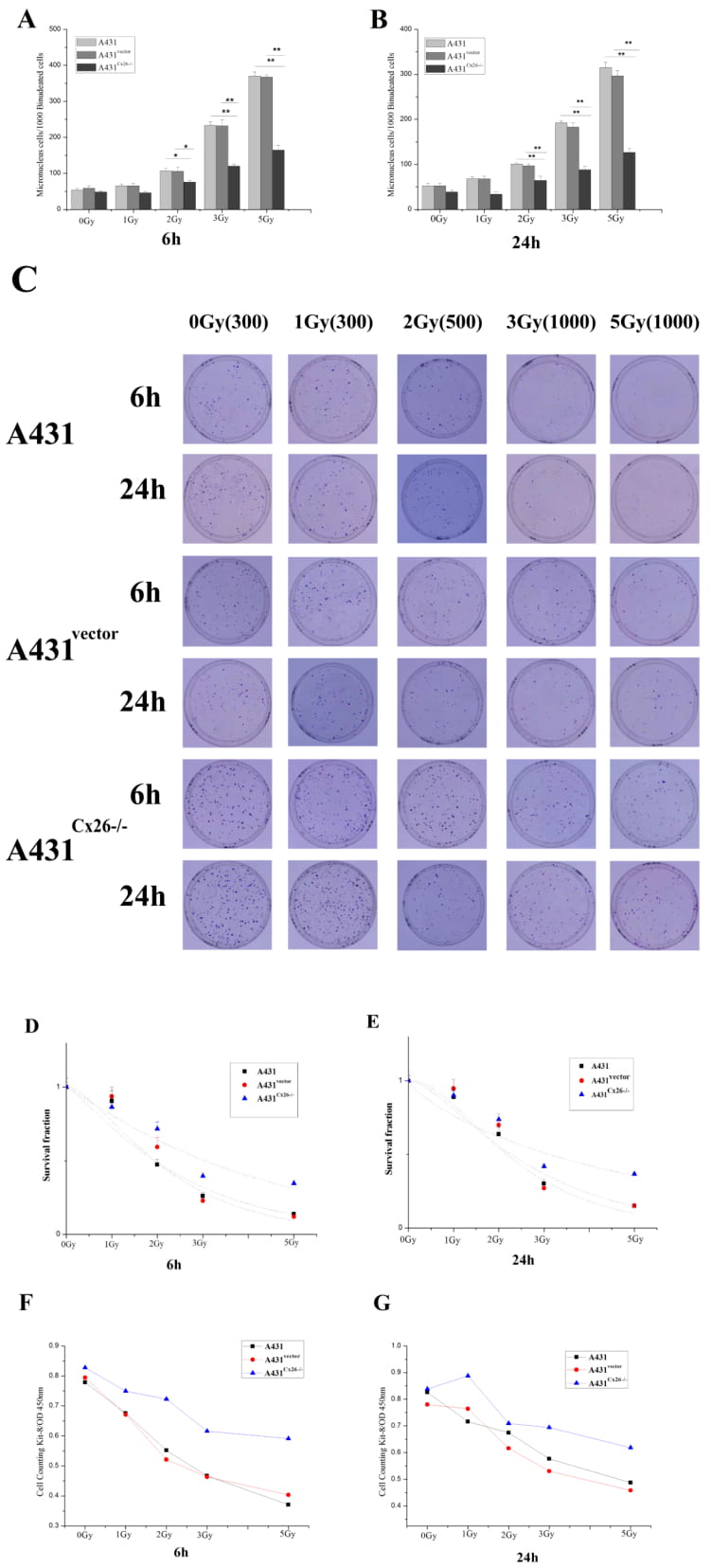

Supplement: Supplementary file 2 [file Data_Sheet_2.ZIP › 672571 fig4/Figure 4-1.jpg]

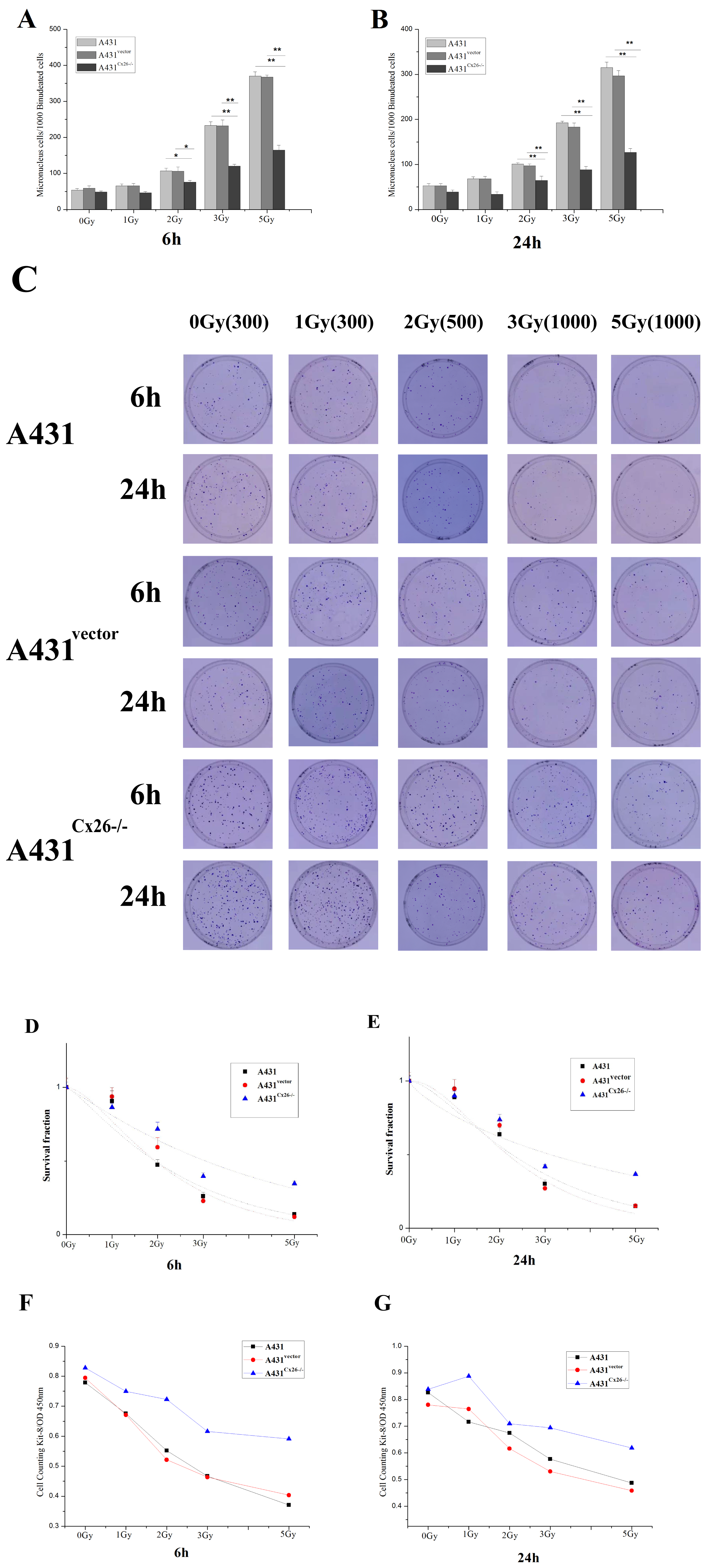

Supplement: Supplementary file 2 [file Data_Sheet_2.ZIP › 672571 fig4/Figure 4.tif]

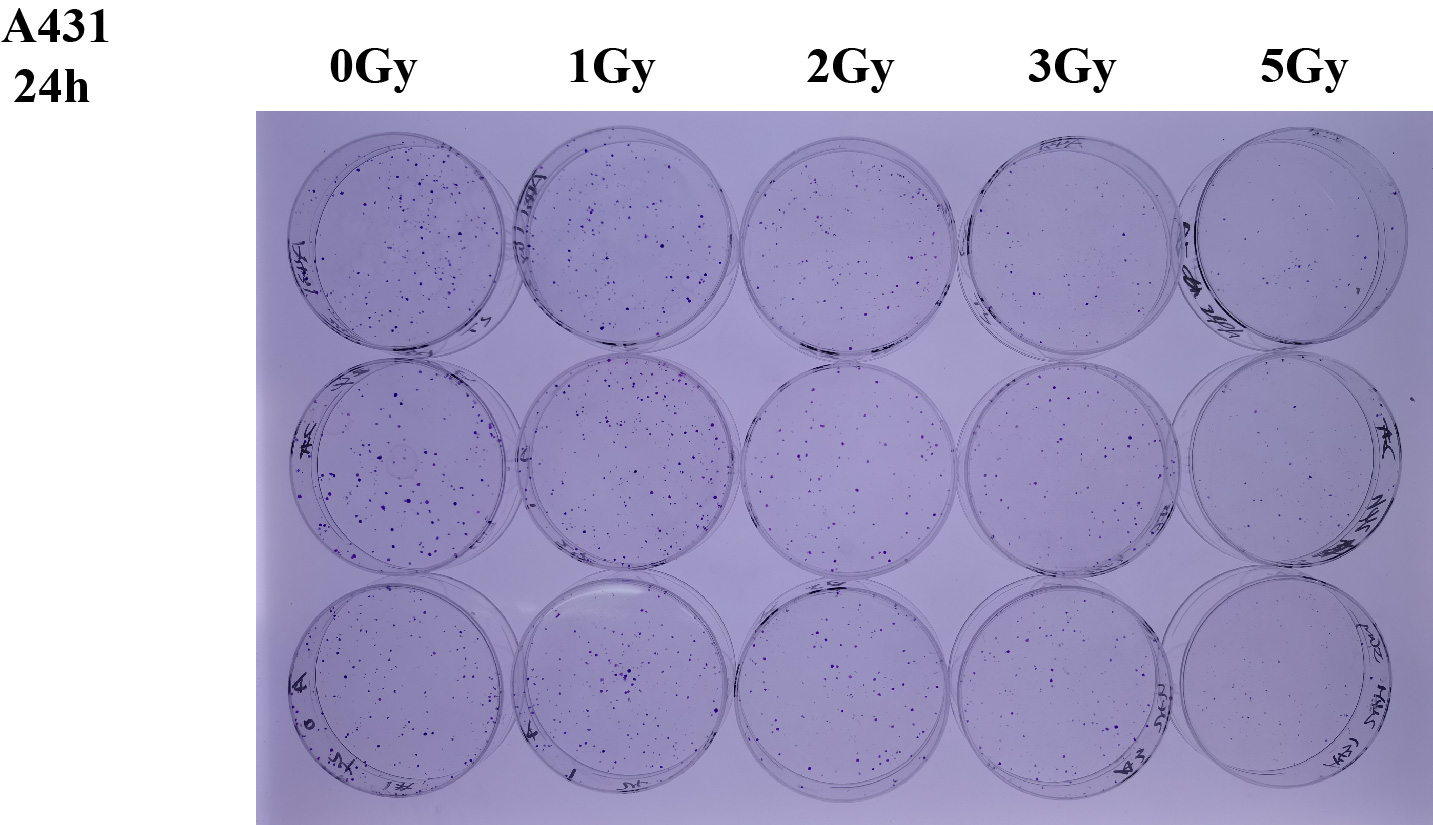

Supplement: Supplementary file 2 [file Data_Sheet_2.ZIP › 672571 fig4/pictures of all dishes/experiment#1/A431 24h.jpg]

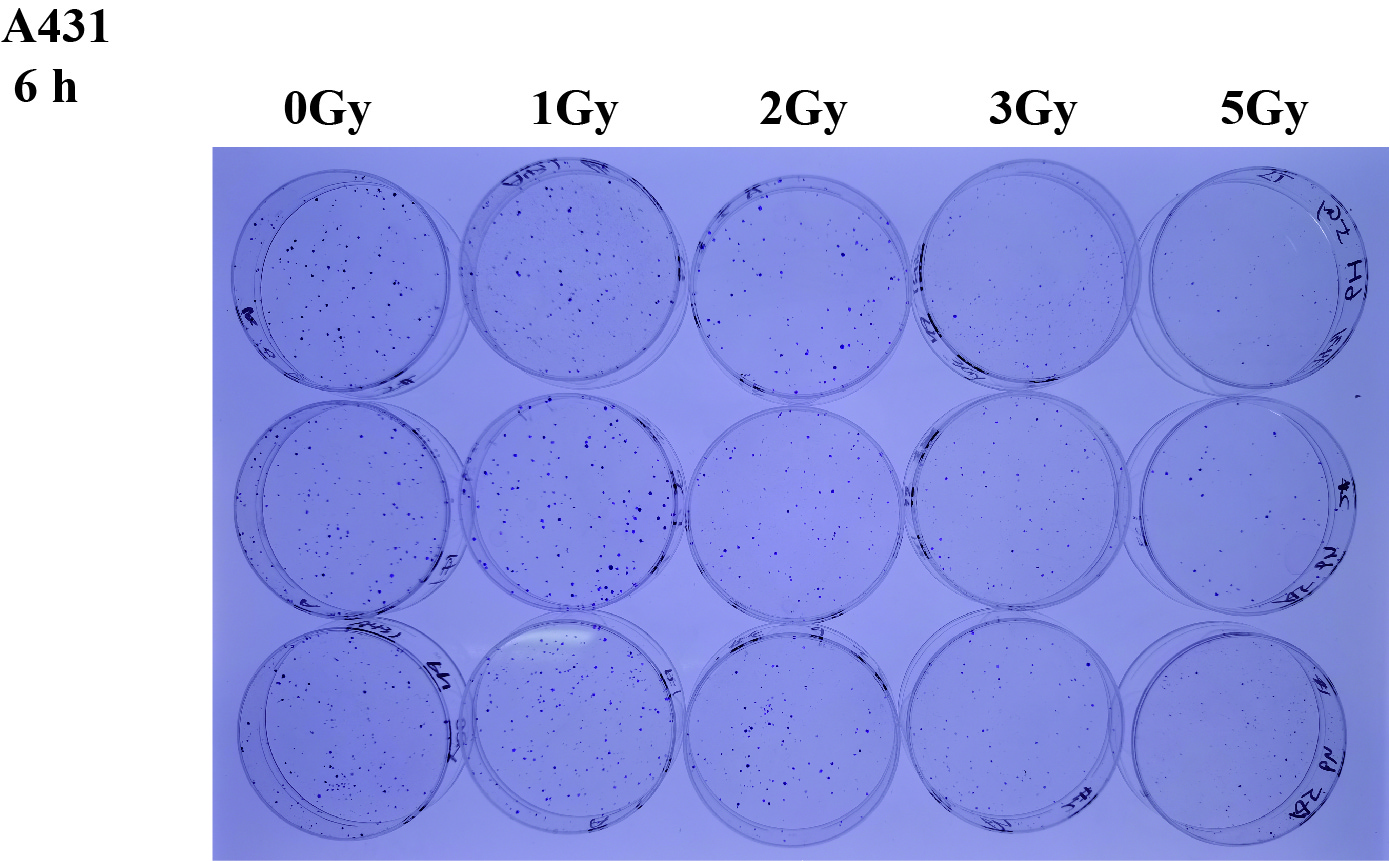

Supplement: Supplementary file 2 [file Data_Sheet_2.ZIP › 672571 fig4/pictures of all dishes/experiment#1/A431 6h.jpg]

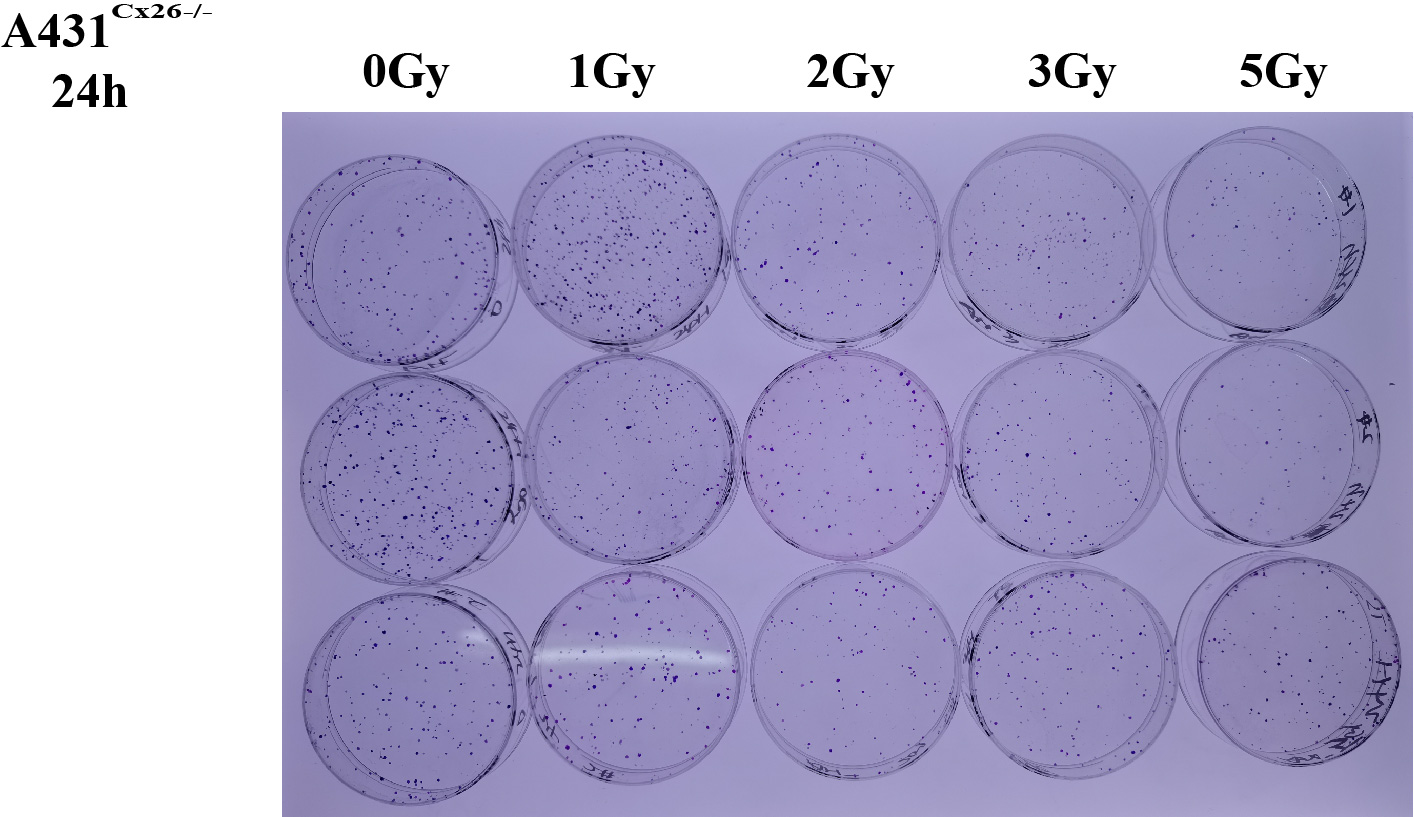

Supplement: Supplementary file 2 [file Data_Sheet_2.ZIP › 672571 fig4/pictures of all dishes/experiment#1/A431Cx26 24h.jpg]

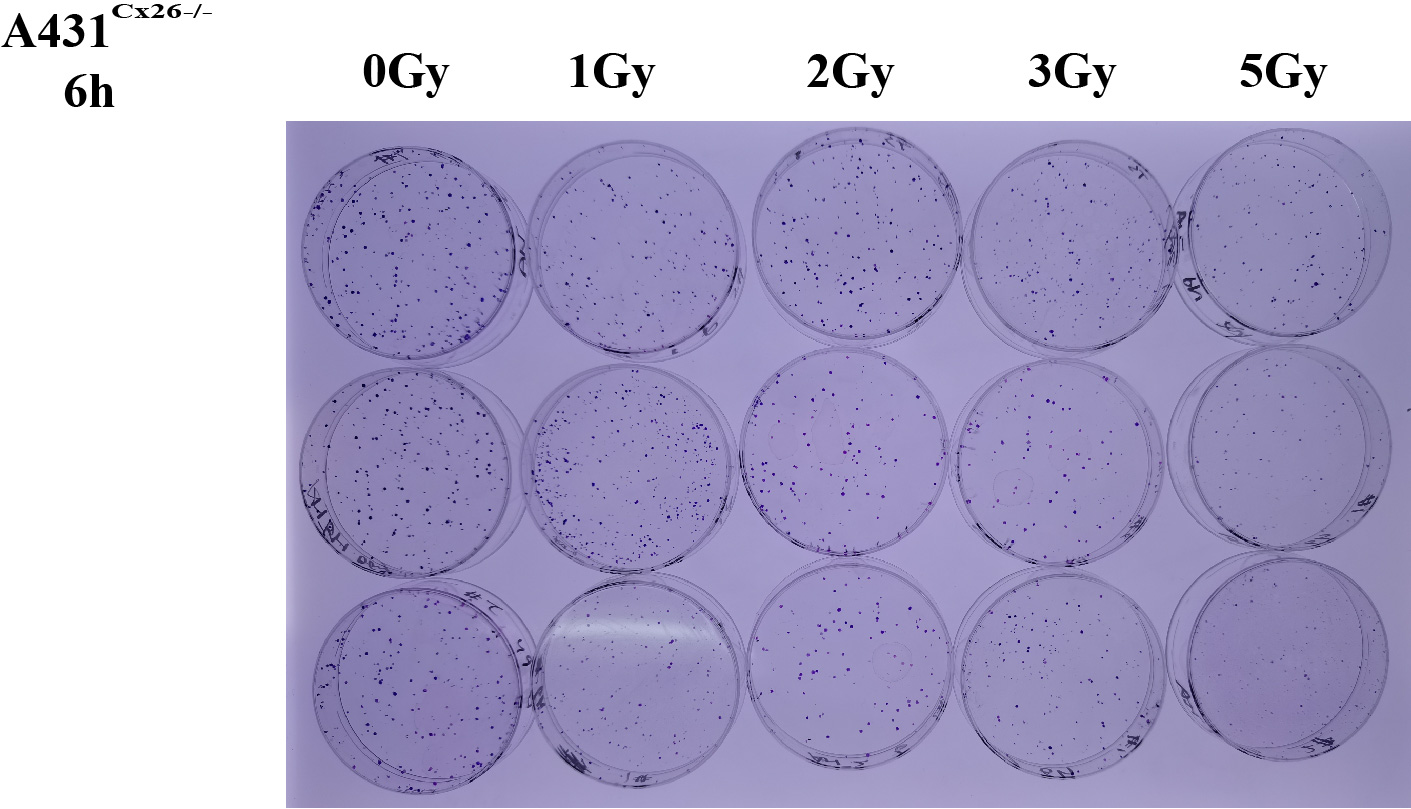

Supplement: Supplementary file 2 [file Data_Sheet_2.ZIP › 672571 fig4/pictures of all dishes/experiment#1/A431Cx26 6h.jpg]

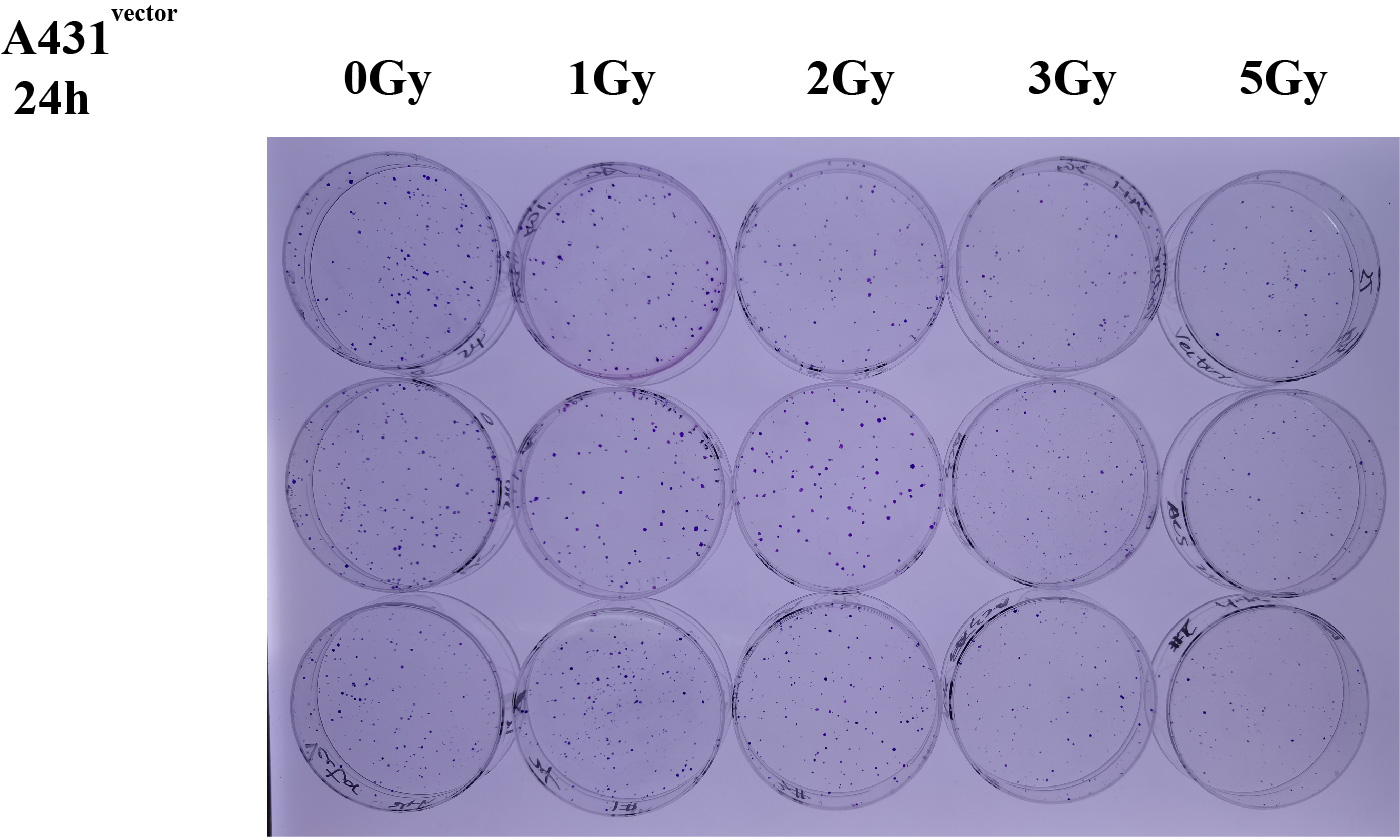

Supplement: Supplementary file 2 [file Data_Sheet_2.ZIP › 672571 fig4/pictures of all dishes/experiment#1/A431vector 24h.jpg]

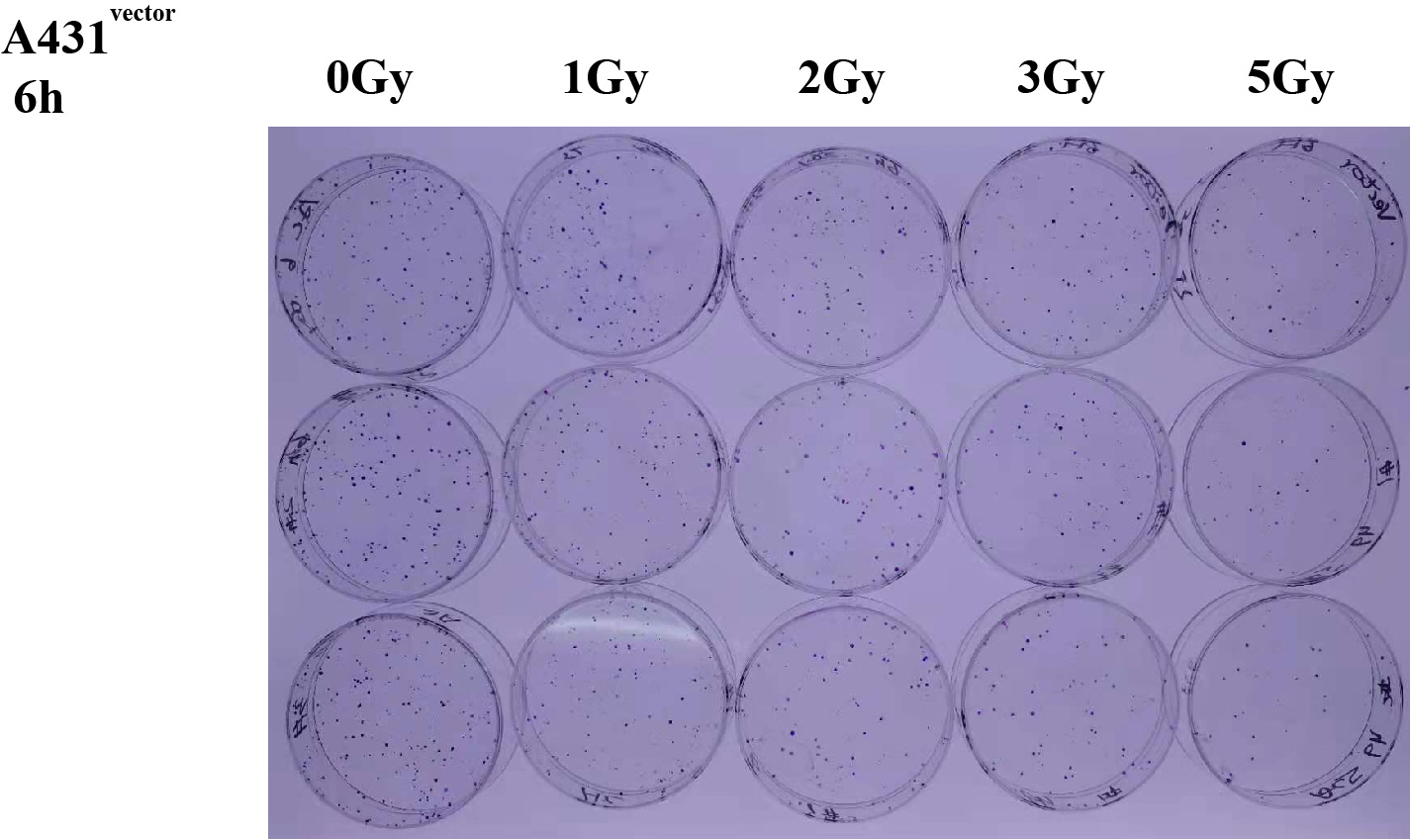

Supplement: Supplementary file 2 [file Data_Sheet_2.ZIP › 672571 fig4/pictures of all dishes/experiment#1/A431vector 6h.jpg]

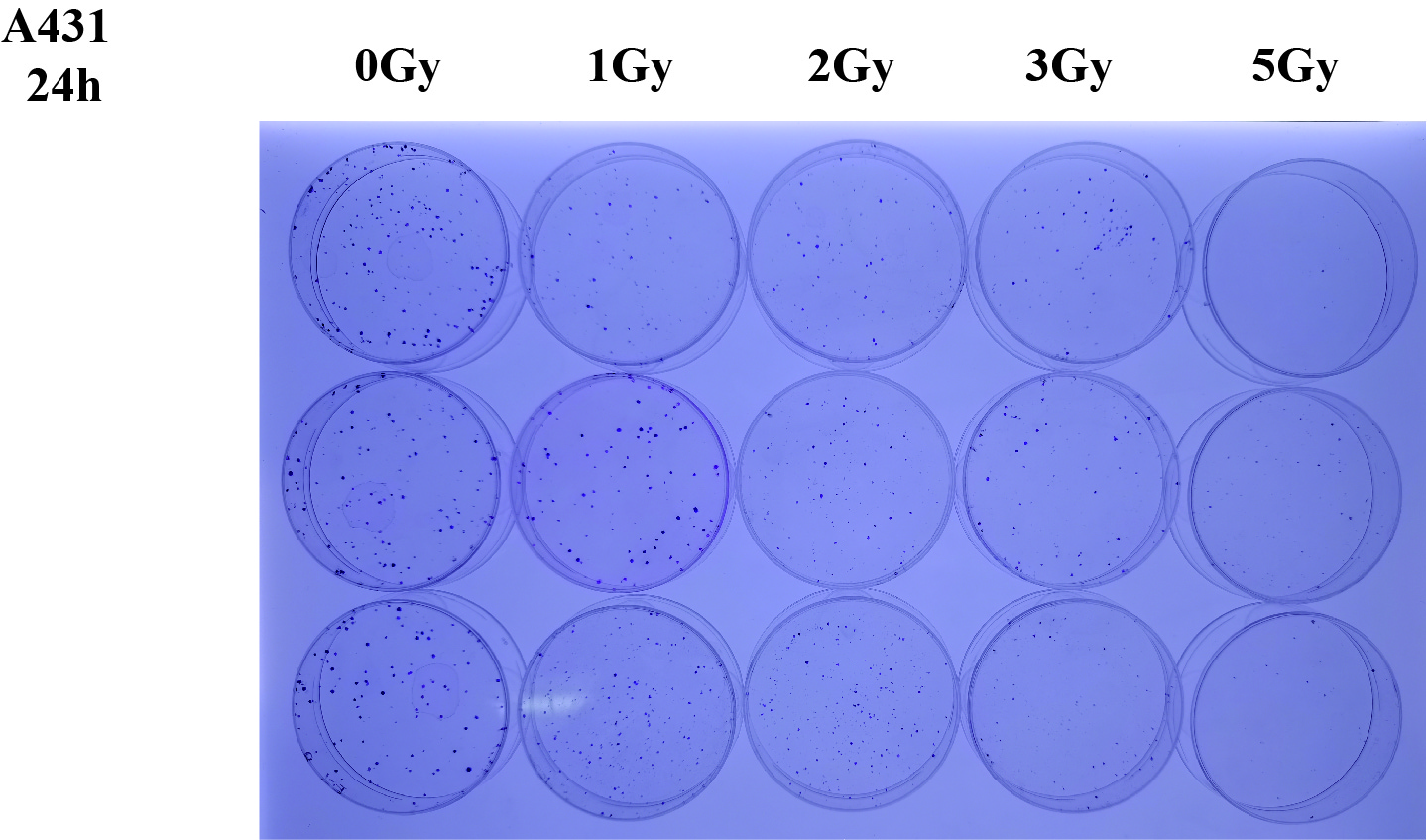

Supplement: Supplementary file 2 [file Data_Sheet_2.ZIP › 672571 fig4/pictures of all dishes/experiment#2/A431 24h.jpg]

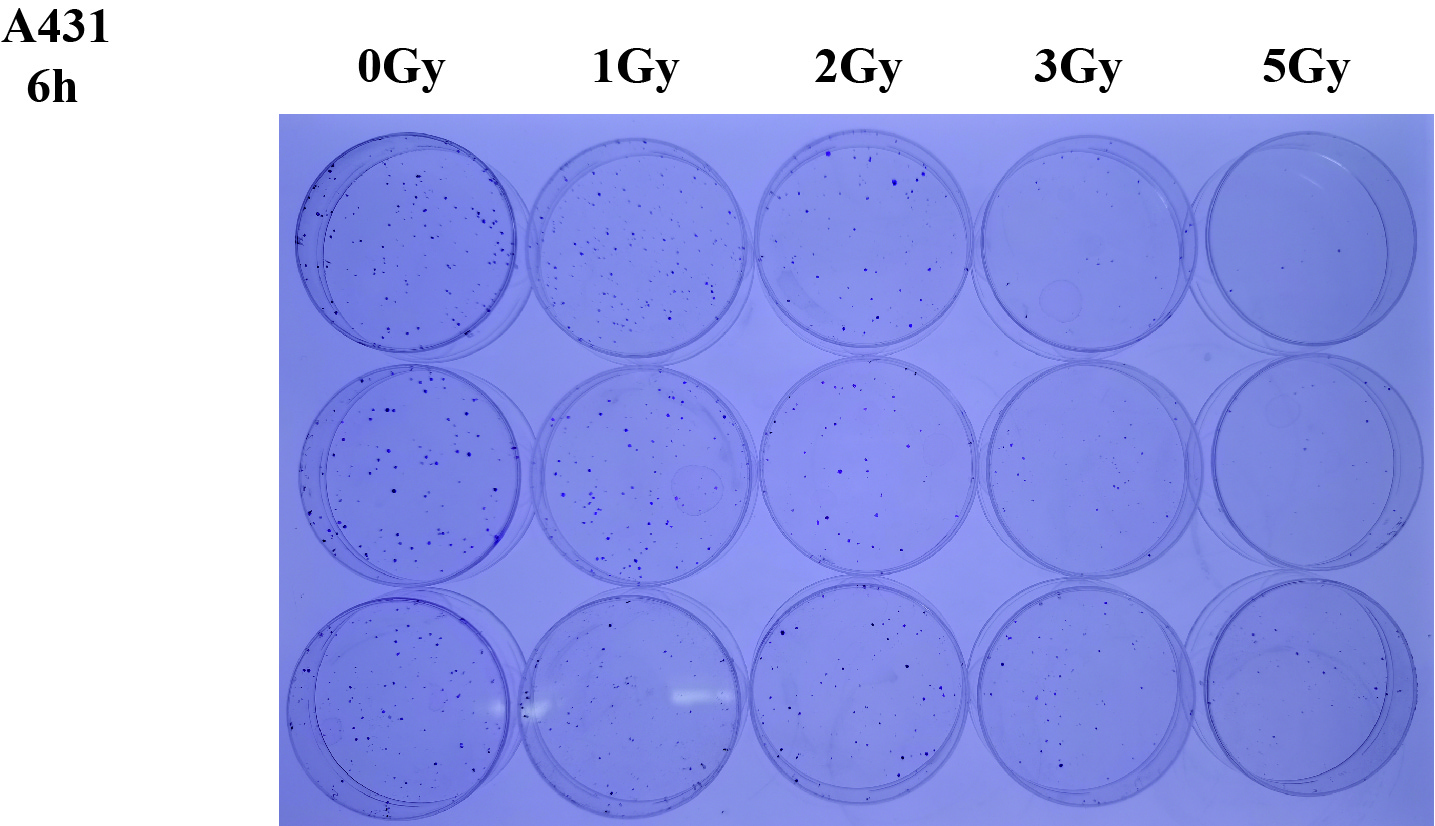

Supplement: Supplementary file 2 [file Data_Sheet_2.ZIP › 672571 fig4/pictures of all dishes/experiment#2/A431 6h.jpg]

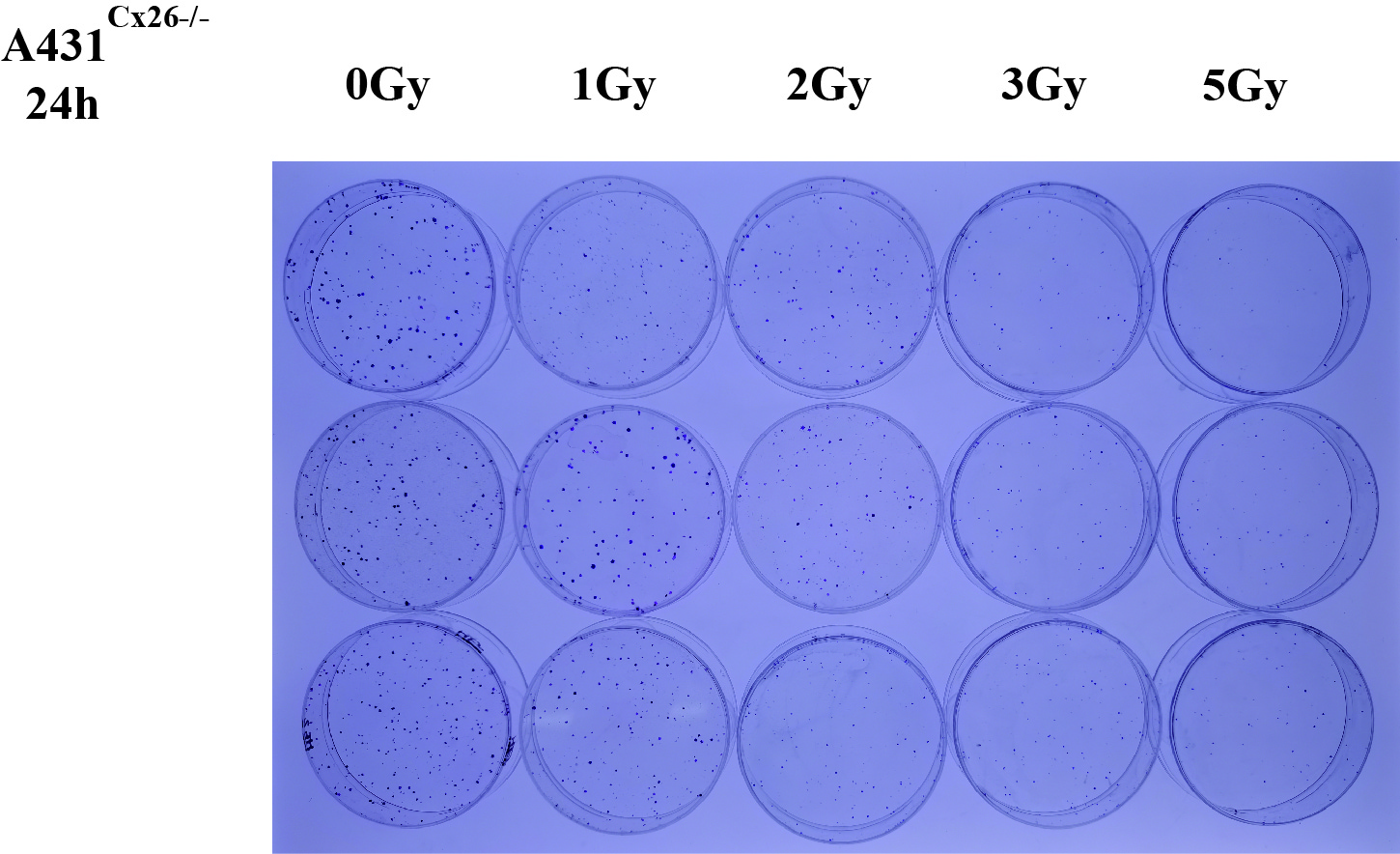

Supplement: Supplementary file 2 [file Data_Sheet_2.ZIP › 672571 fig4/pictures of all dishes/experiment#2/A431Cx26- 24h.jpg]

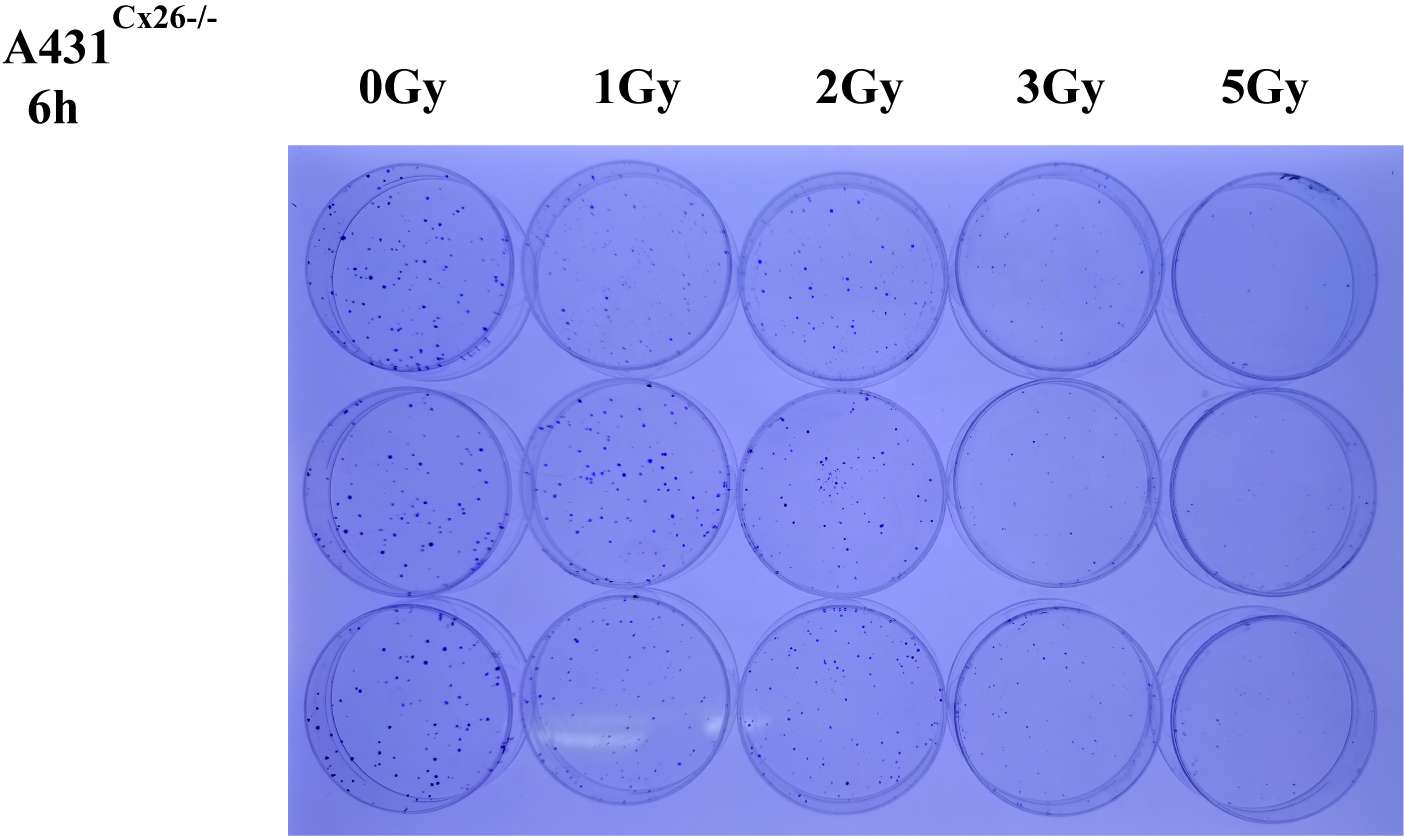

Supplement: Supplementary file 2 [file Data_Sheet_2.ZIP › 672571 fig4/pictures of all dishes/experiment#2/A431Cx26- 6h.jpg]

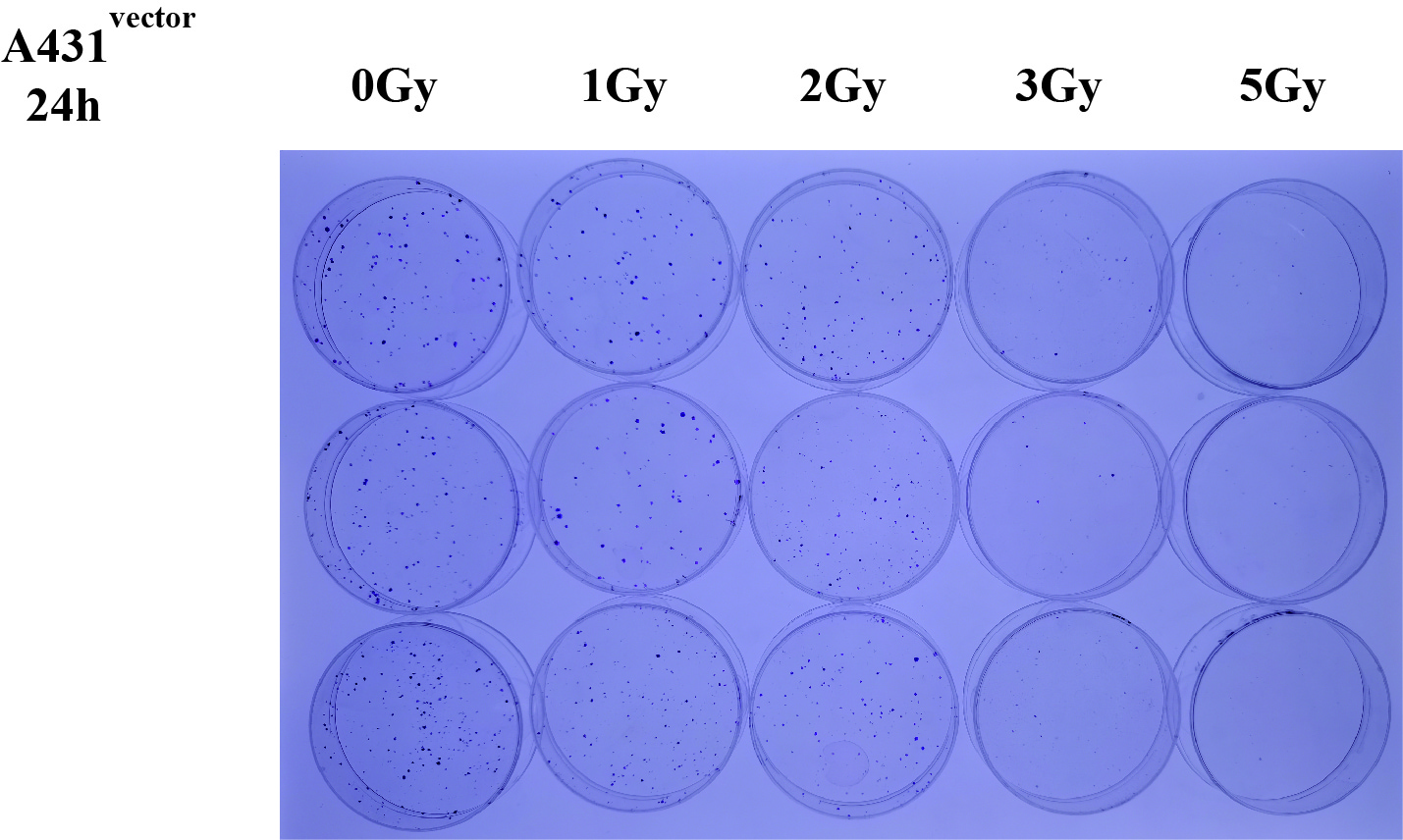

Supplement: Supplementary file 2 [file Data_Sheet_2.ZIP › 672571 fig4/pictures of all dishes/experiment#2/A431vector 24h.jpg]

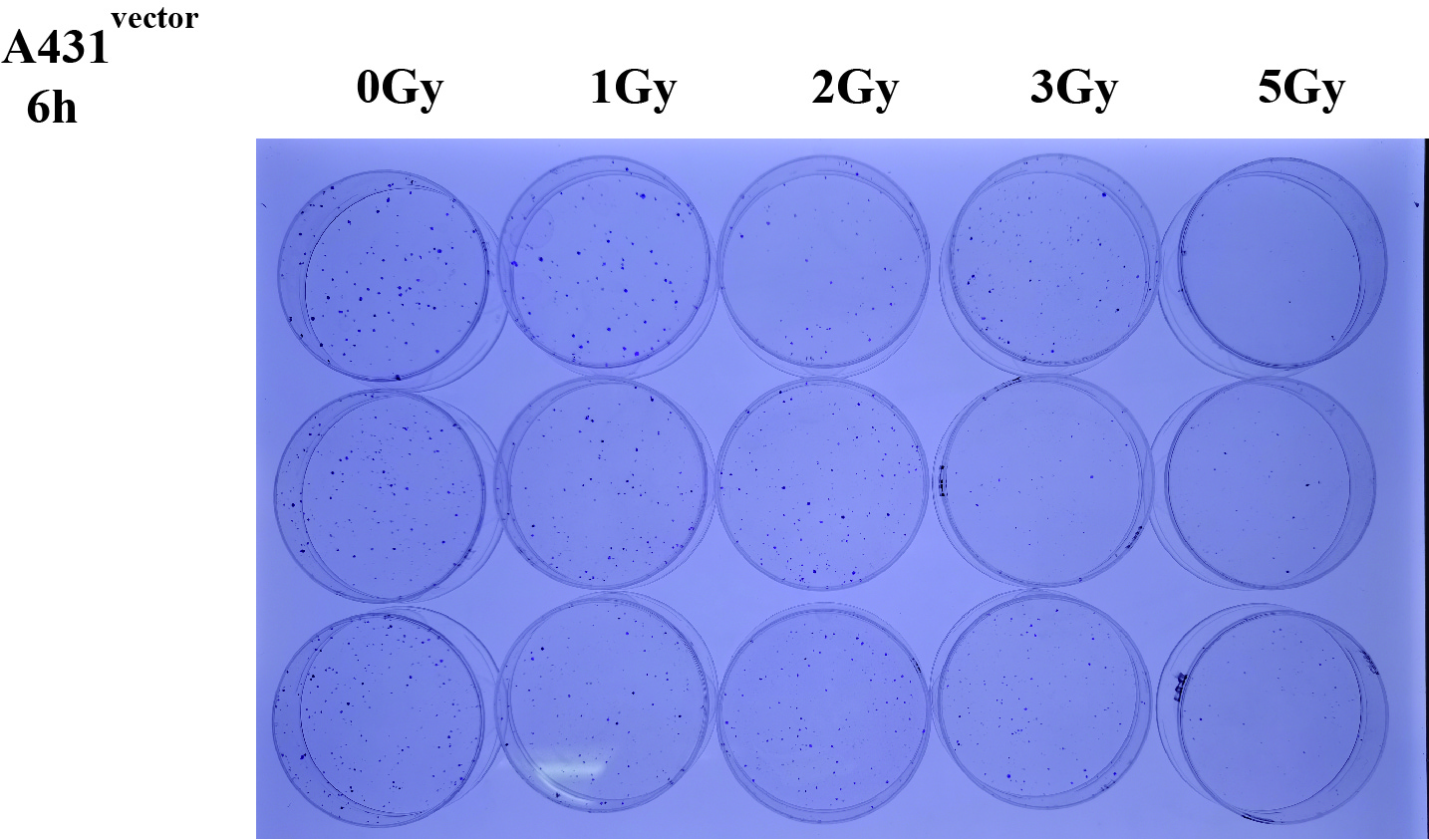

Supplement: Supplementary file 2 [file Data_Sheet_2.ZIP › 672571 fig4/pictures of all dishes/experiment#2/A431vector 6h.jpg]

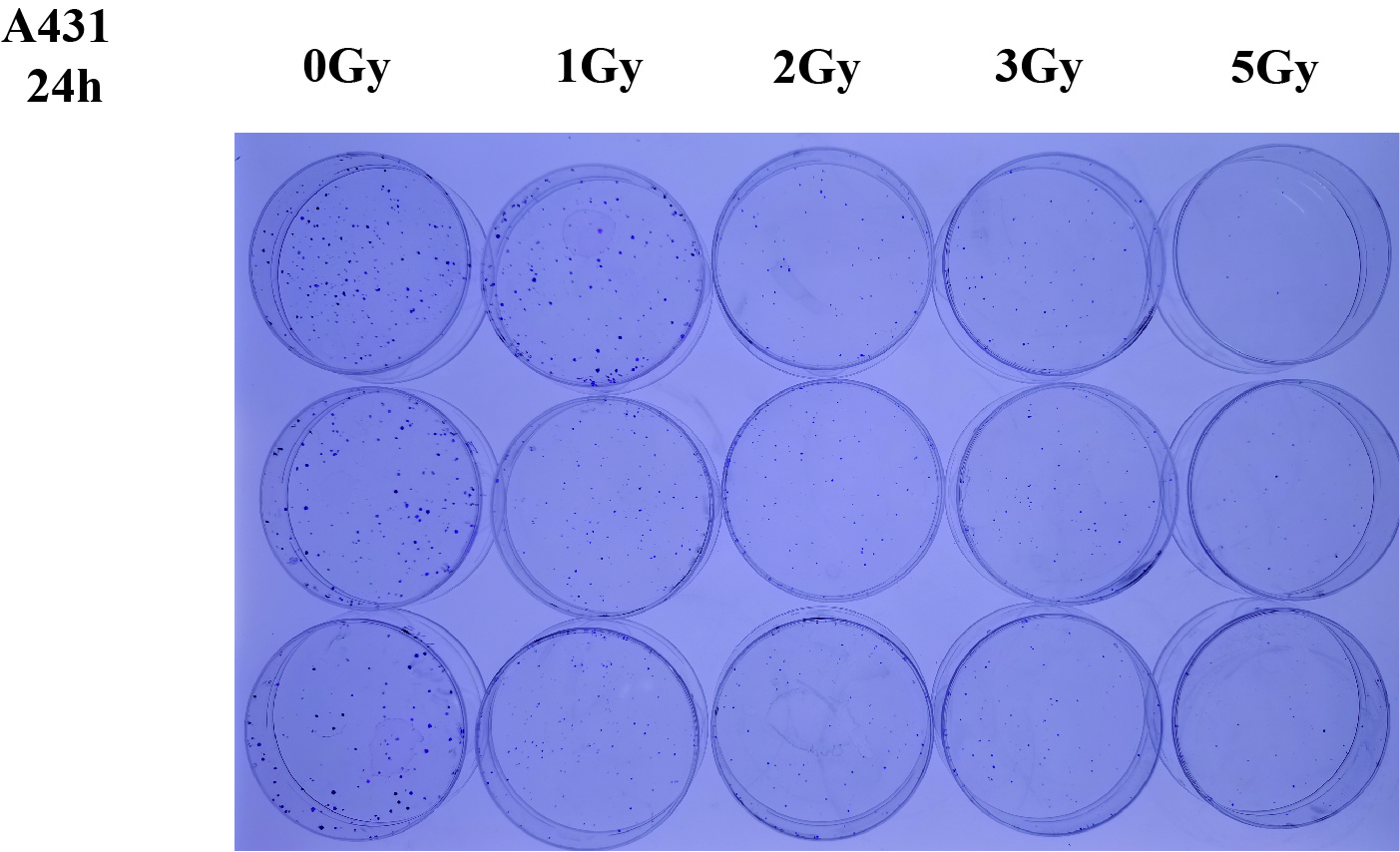

Supplement: Supplementary file 2 [file Data_Sheet_2.ZIP › 672571 fig4/pictures of all dishes/experiment#3/A431 24h.jpg]

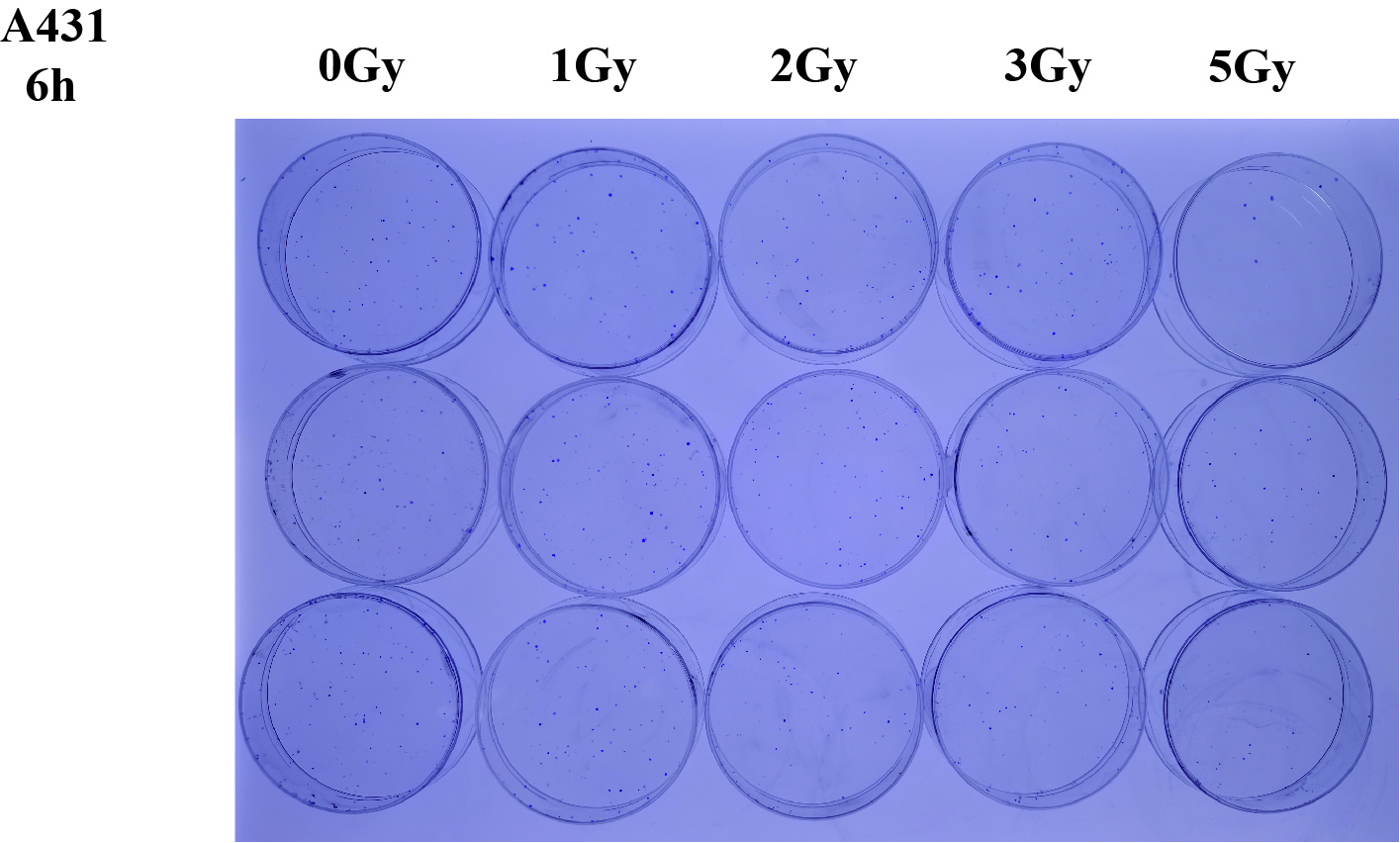

Supplement: Supplementary file 2 [file Data_Sheet_2.ZIP › 672571 fig4/pictures of all dishes/experiment#3/A431 6h.jpg]

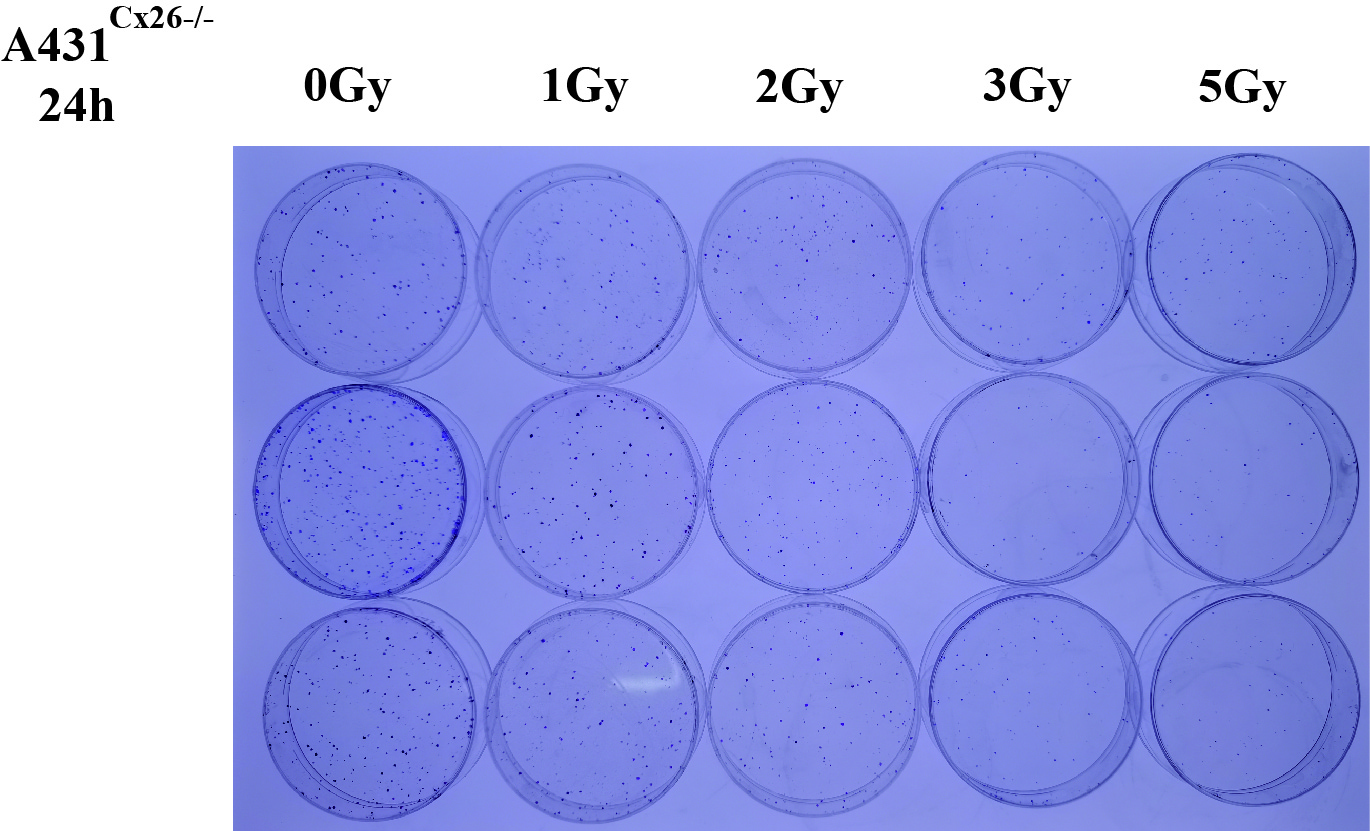

Supplement: Supplementary file 2 [file Data_Sheet_2.ZIP › 672571 fig4/pictures of all dishes/experiment#3/A431 Cx26- 24h.jpg]

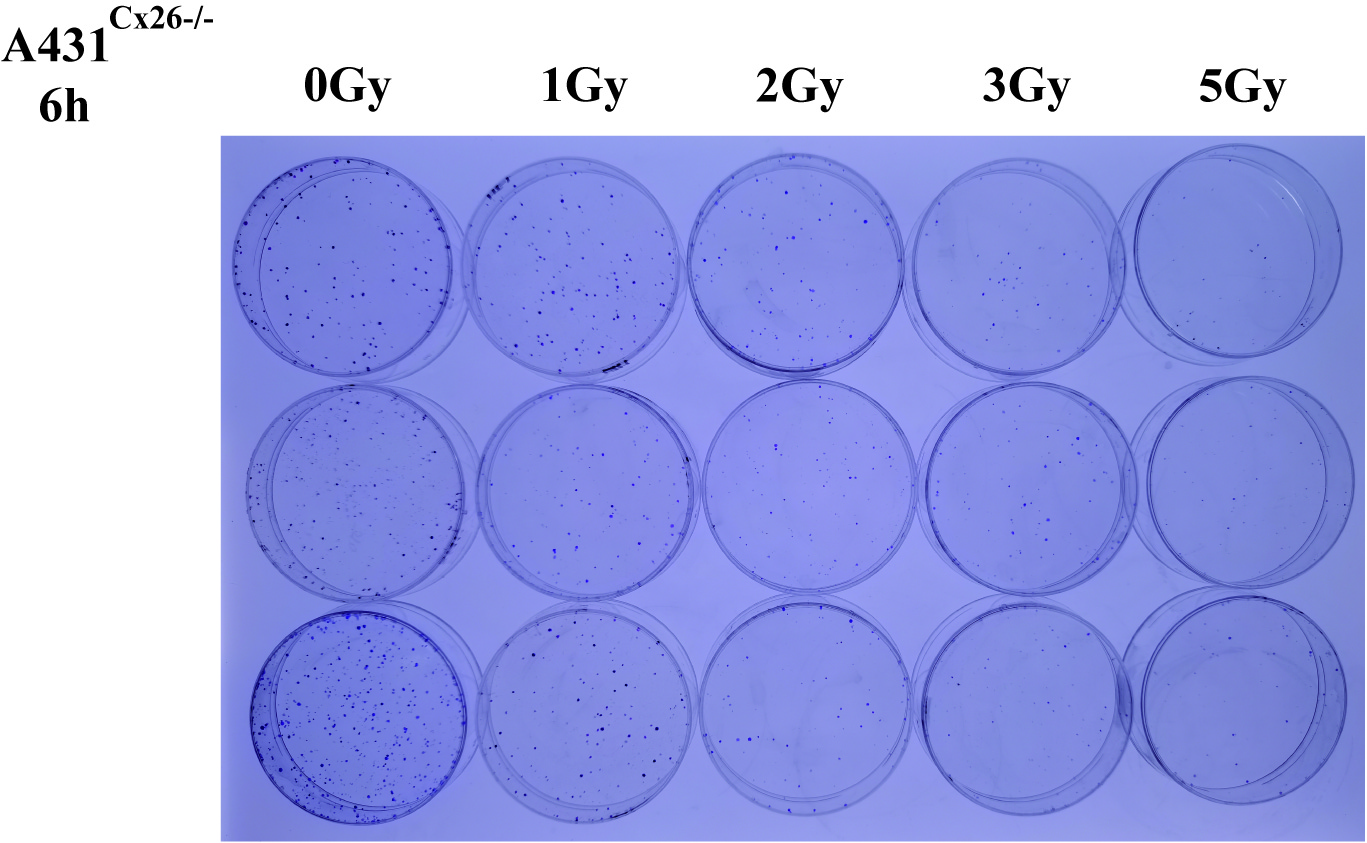

Supplement: Supplementary file 2 [file Data_Sheet_2.ZIP › 672571 fig4/pictures of all dishes/experiment#3/A431 Cx26- 6h.jpg]

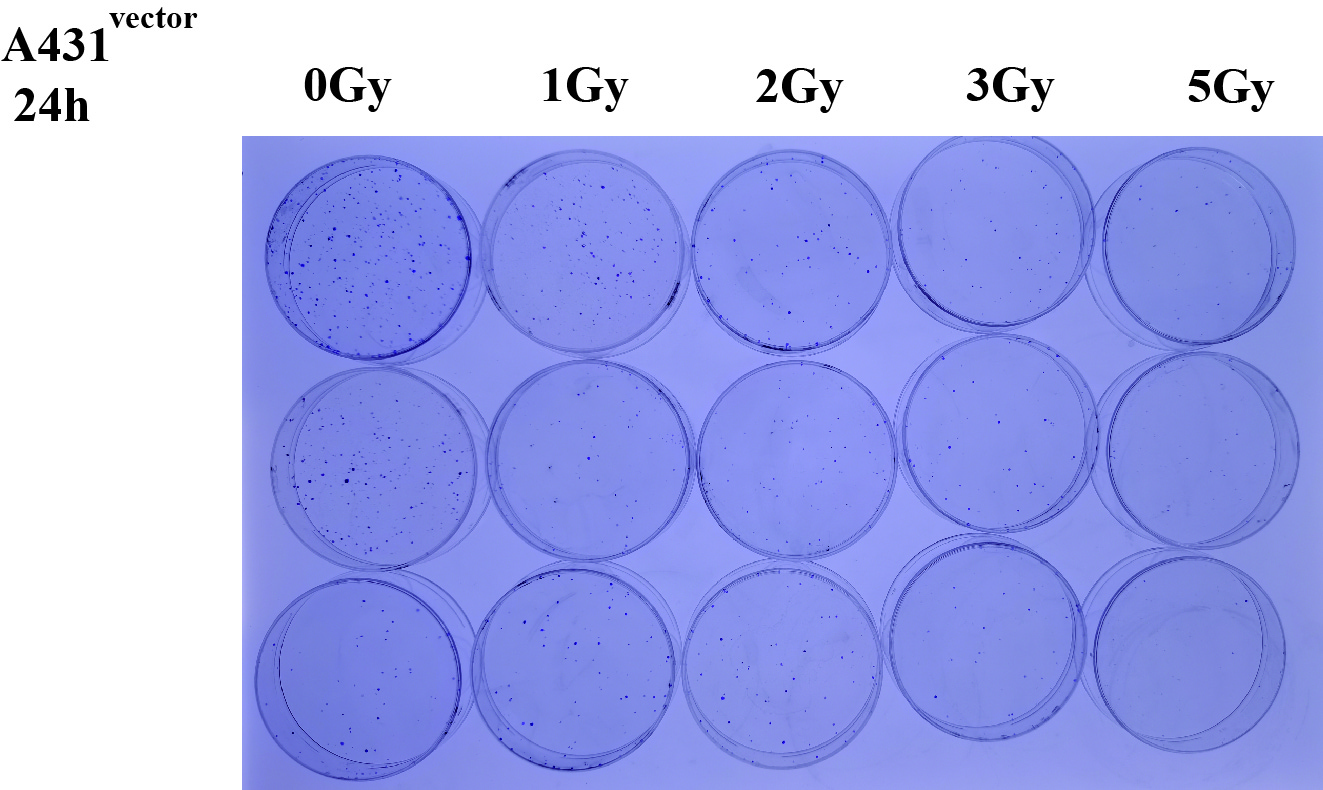

Supplement: Supplementary file 2 [file Data_Sheet_2.ZIP › 672571 fig4/pictures of all dishes/experiment#3/A431 vector 24h.jpg]

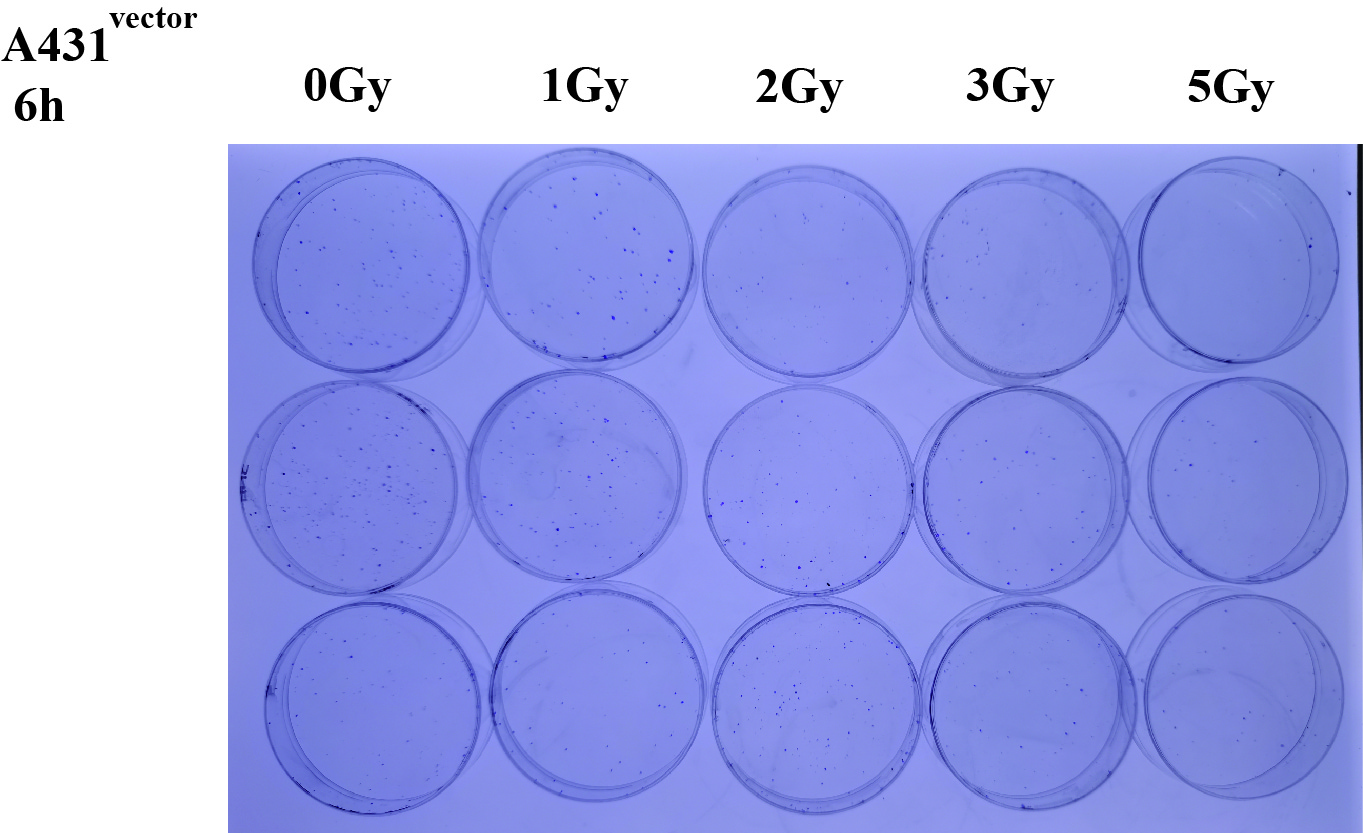

Supplement: Supplementary file 2 [file Data_Sheet_2.ZIP › 672571 fig4/pictures of all dishes/experiment#3/A431 vector 6h.jpg]

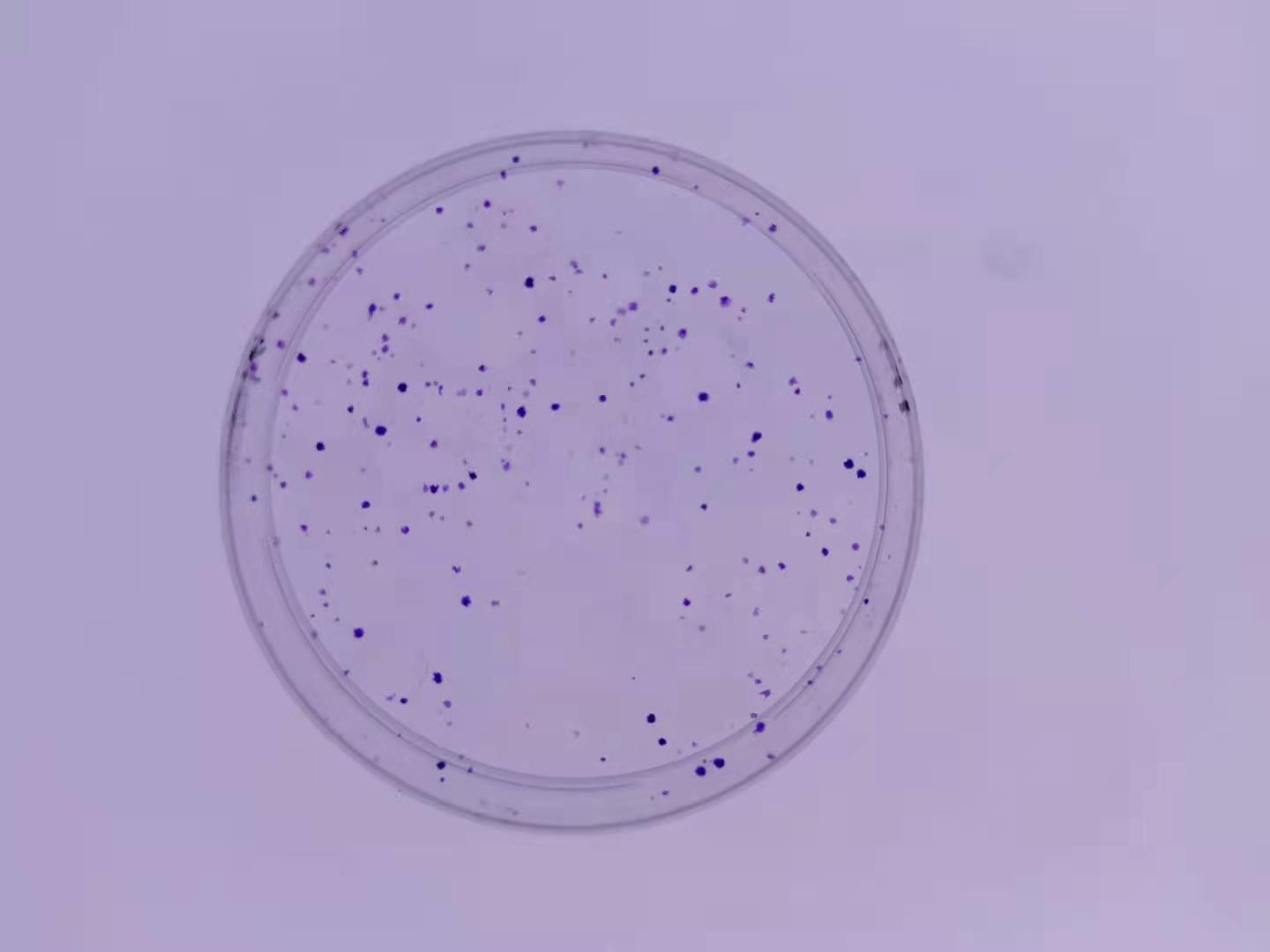

Supplement: Supplementary file 2 [file Data_Sheet_2.ZIP › 672571 fig4/pitures for fig4c/A431/24h/0 Gy.jpg]

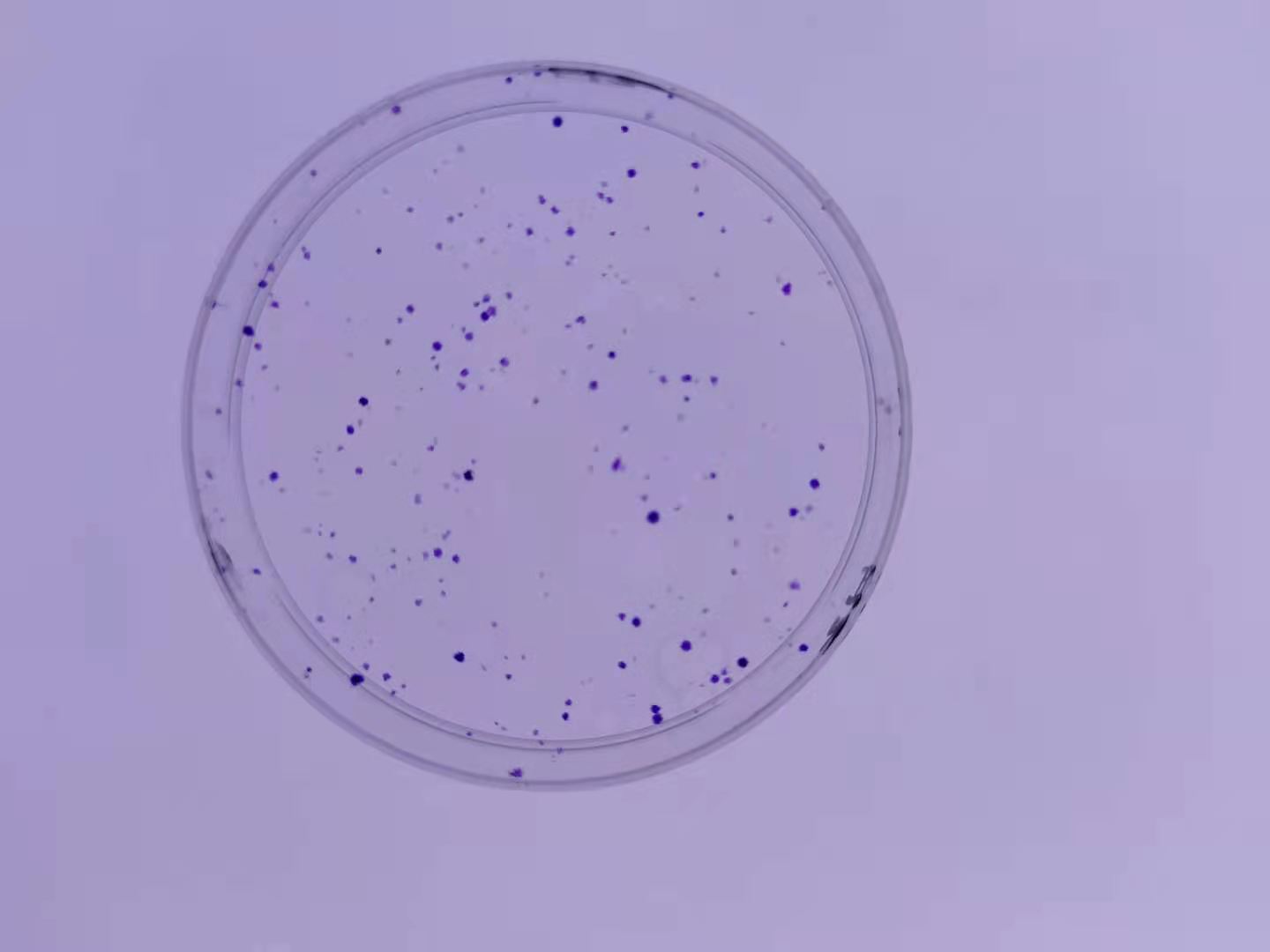

Supplement: Supplementary file 2 [file Data_Sheet_2.ZIP › 672571 fig4/pitures for fig4c/A431/24h/1 Gy.jpg]

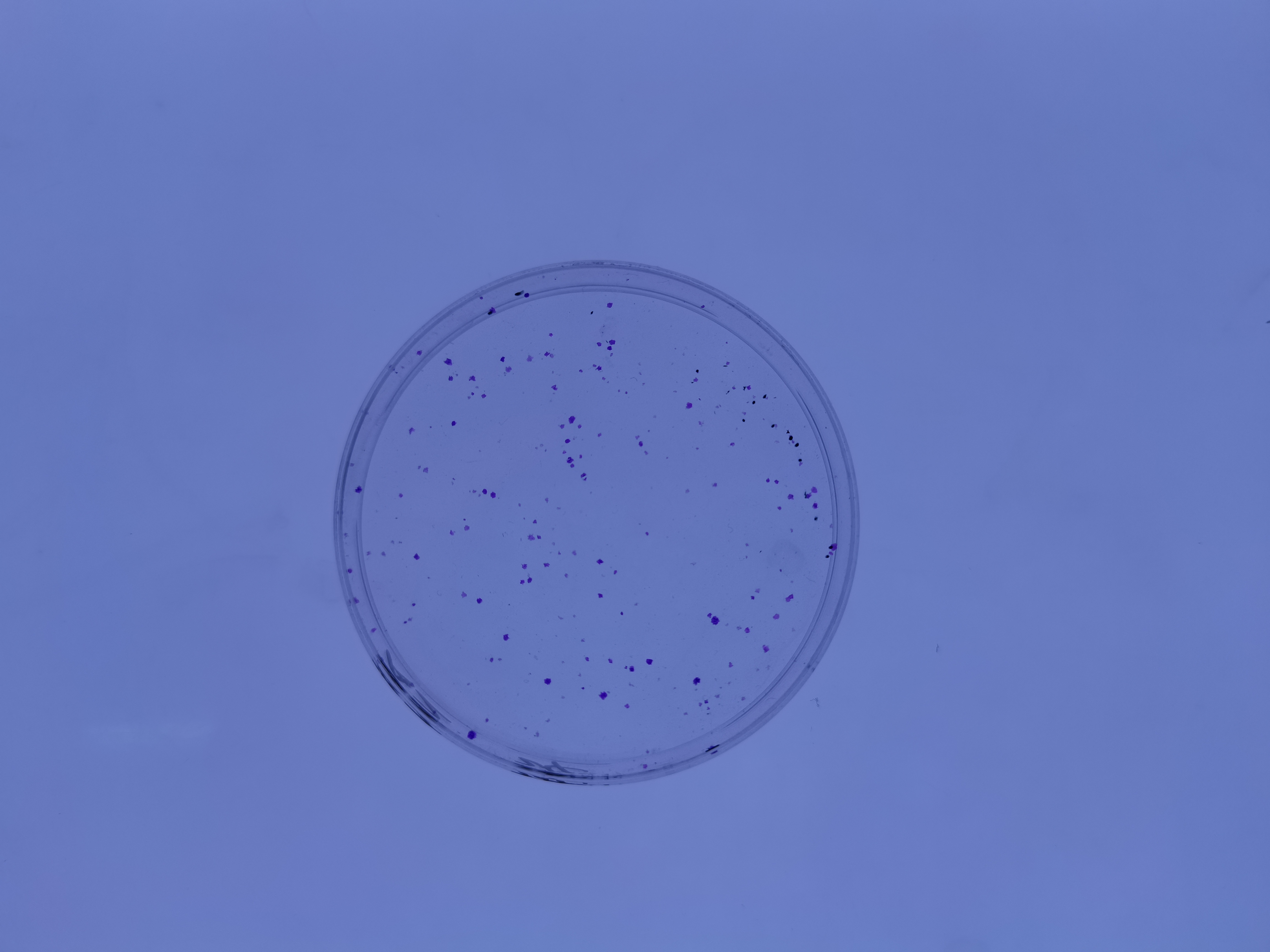

Supplement: Supplementary file 2 [file Data_Sheet_2.ZIP › 672571 fig4/pitures for fig4c/A431/24h/2 Gy.jpg]

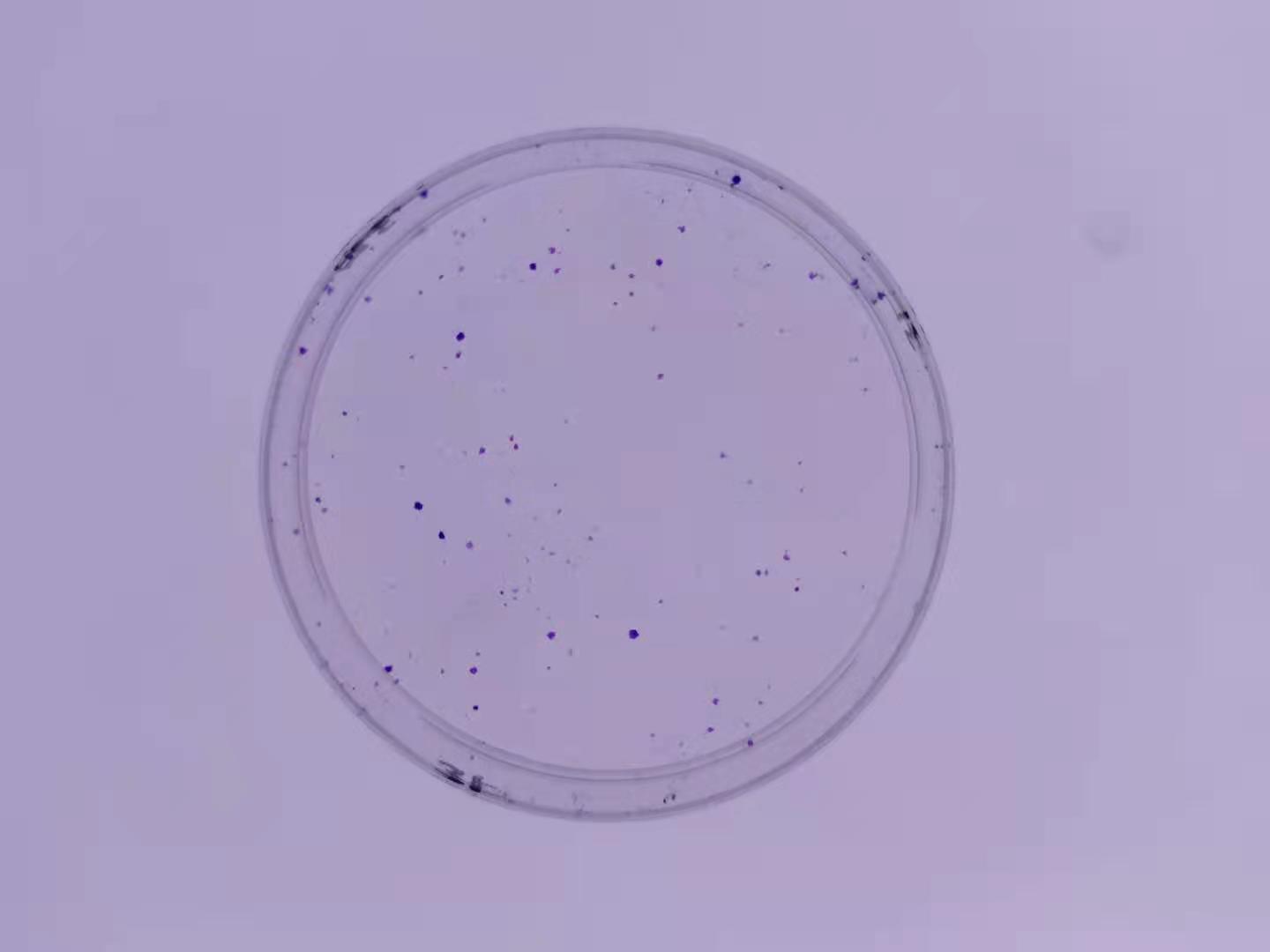

Supplement: Supplementary file 2 [file Data_Sheet_2.ZIP › 672571 fig4/pitures for fig4c/A431/24h/3 Gy.jpg]

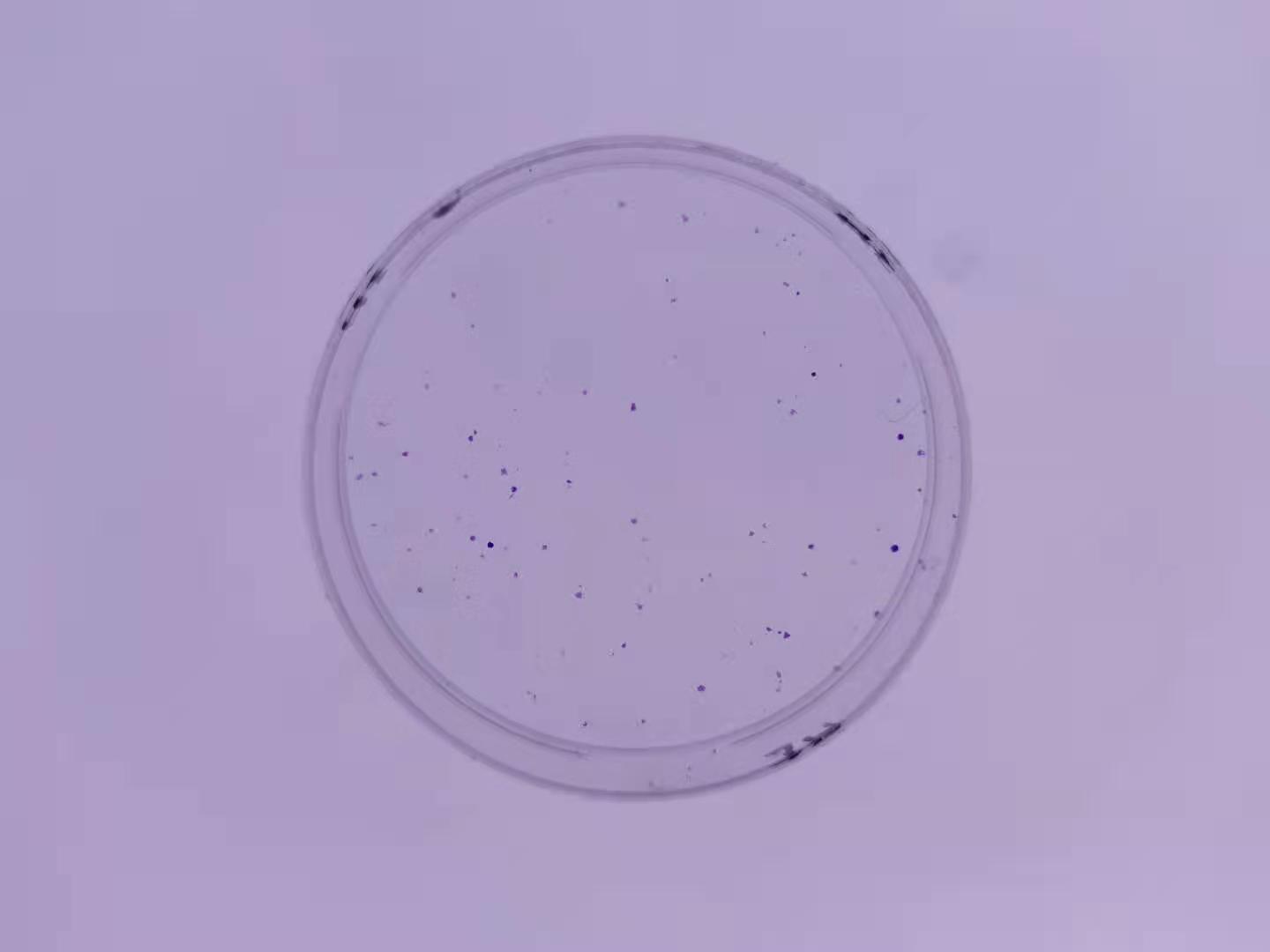

Supplement: Supplementary file 2 [file Data_Sheet_2.ZIP › 672571 fig4/pitures for fig4c/A431/24h/5 Gy.jpg]

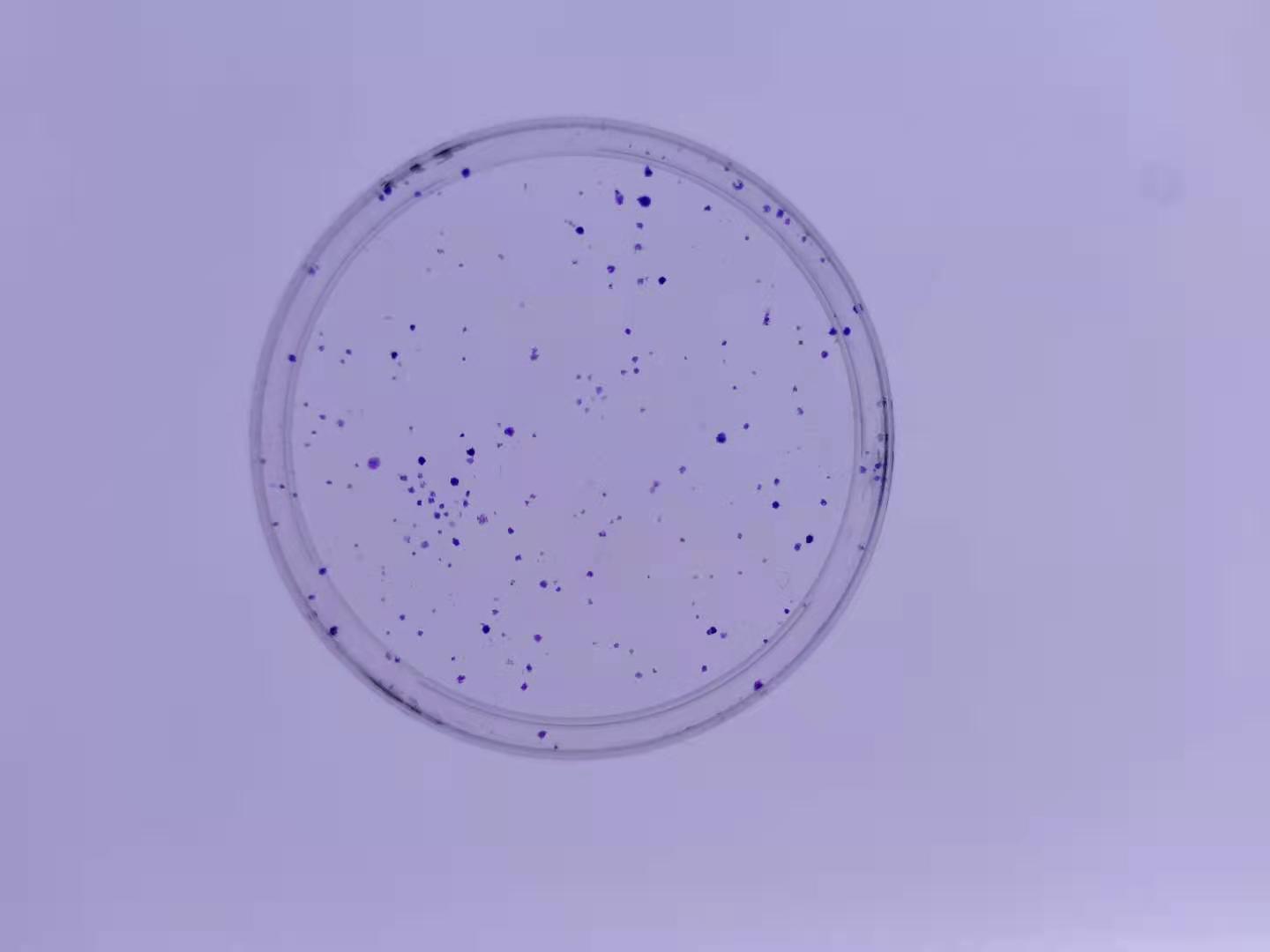

Supplement: Supplementary file 2 [file Data_Sheet_2.ZIP › 672571 fig4/pitures for fig4c/A431/6h/0 Gy.jpg]

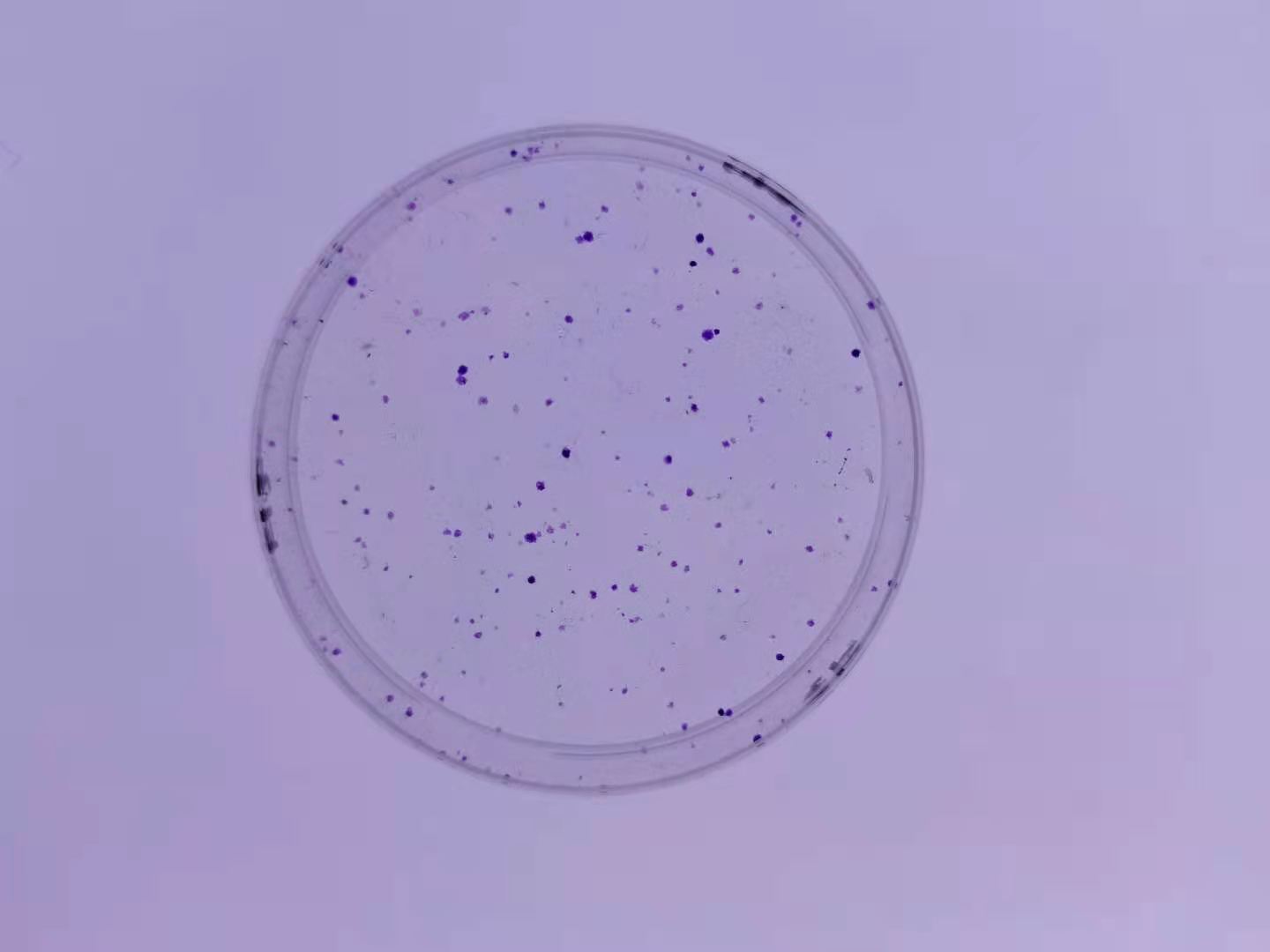

Supplement: Supplementary file 2 [file Data_Sheet_2.ZIP › 672571 fig4/pitures for fig4c/A431/6h/1 Gy.jpg]

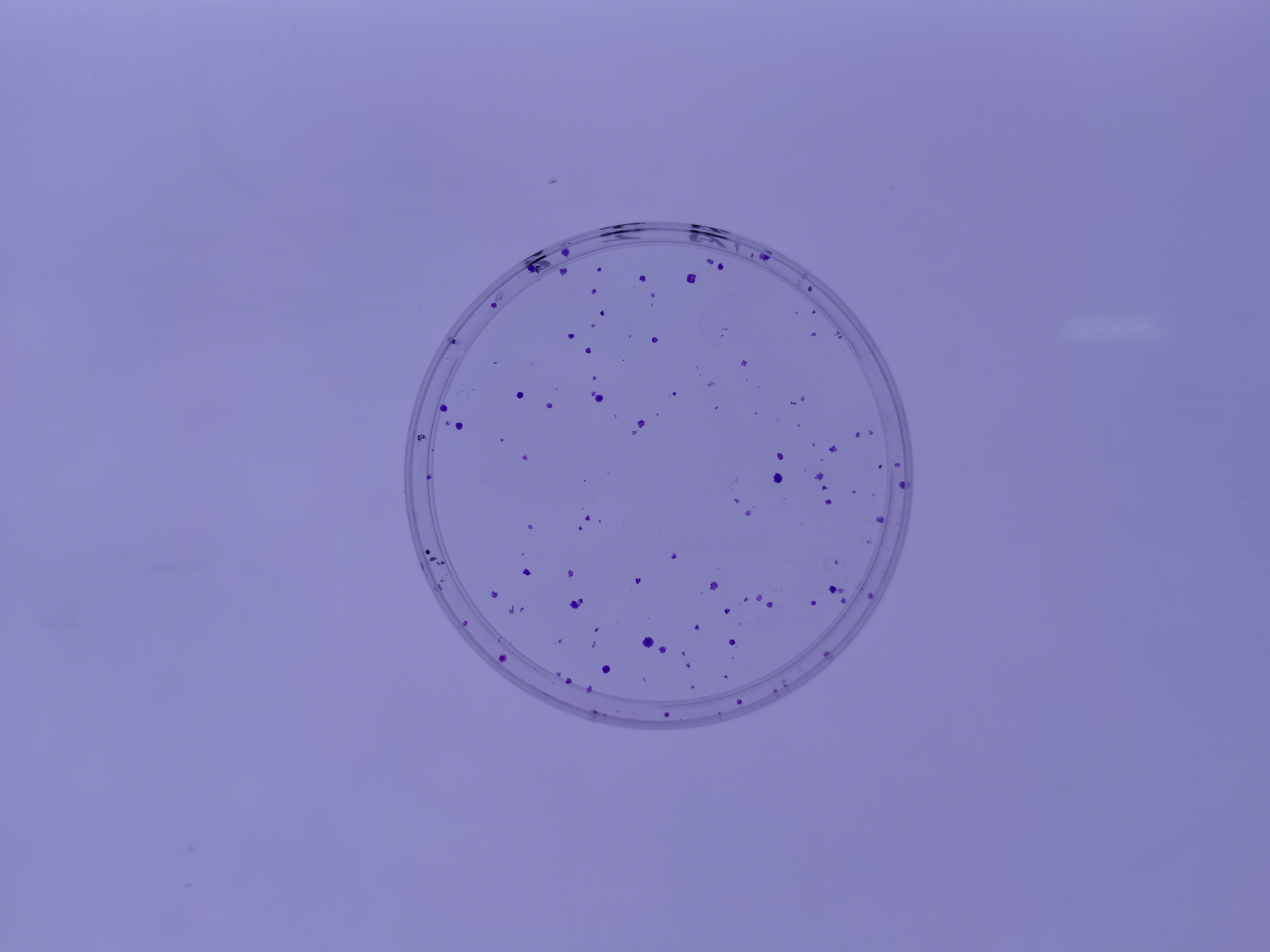

Supplement: Supplementary file 2 [file Data_Sheet_2.ZIP › 672571 fig4/pitures for fig4c/A431/6h/2 Gy.jpg]

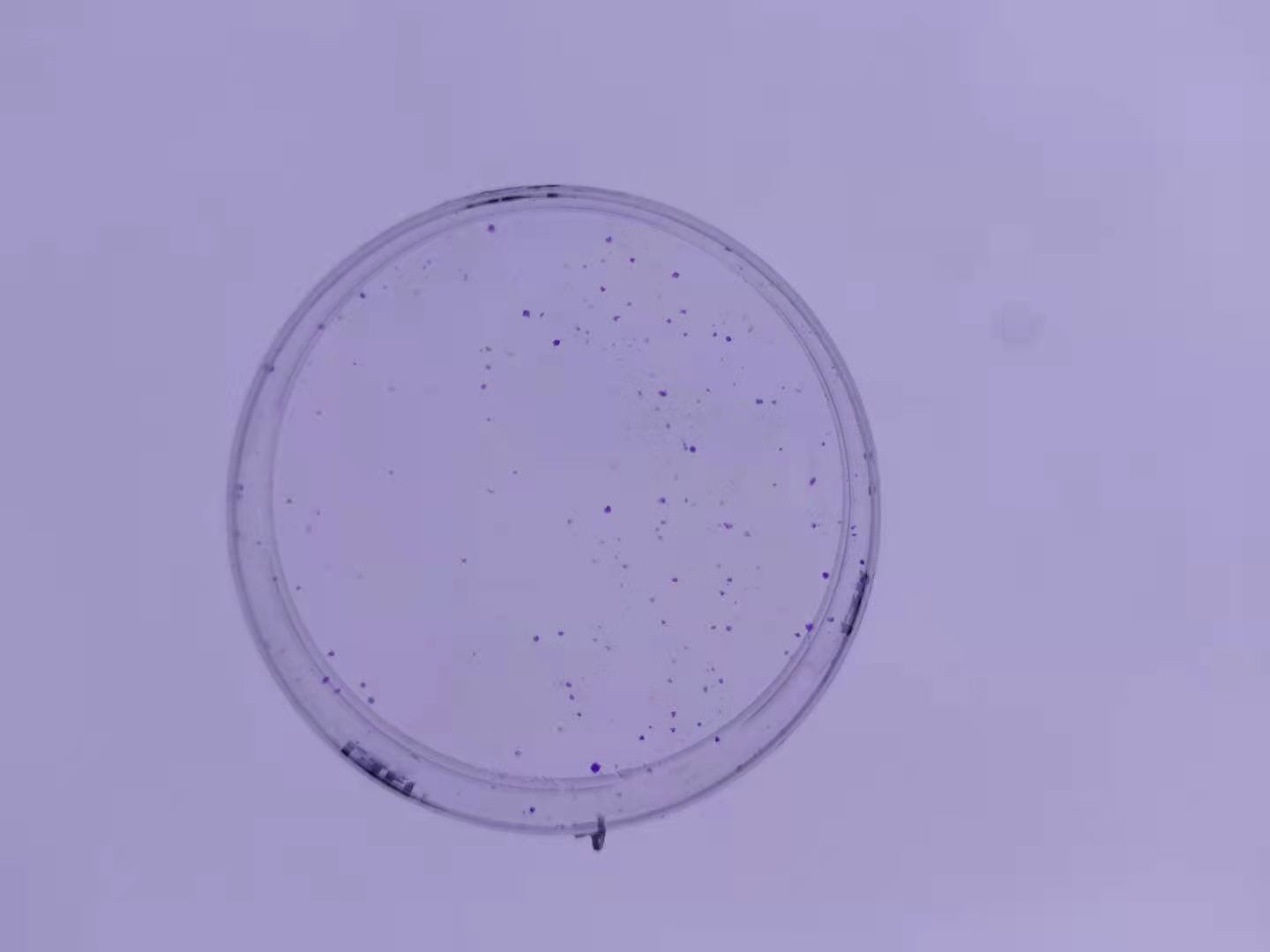

Supplement: Supplementary file 2 [file Data_Sheet_2.ZIP › 672571 fig4/pitures for fig4c/A431/6h/3 Gy.jpg]

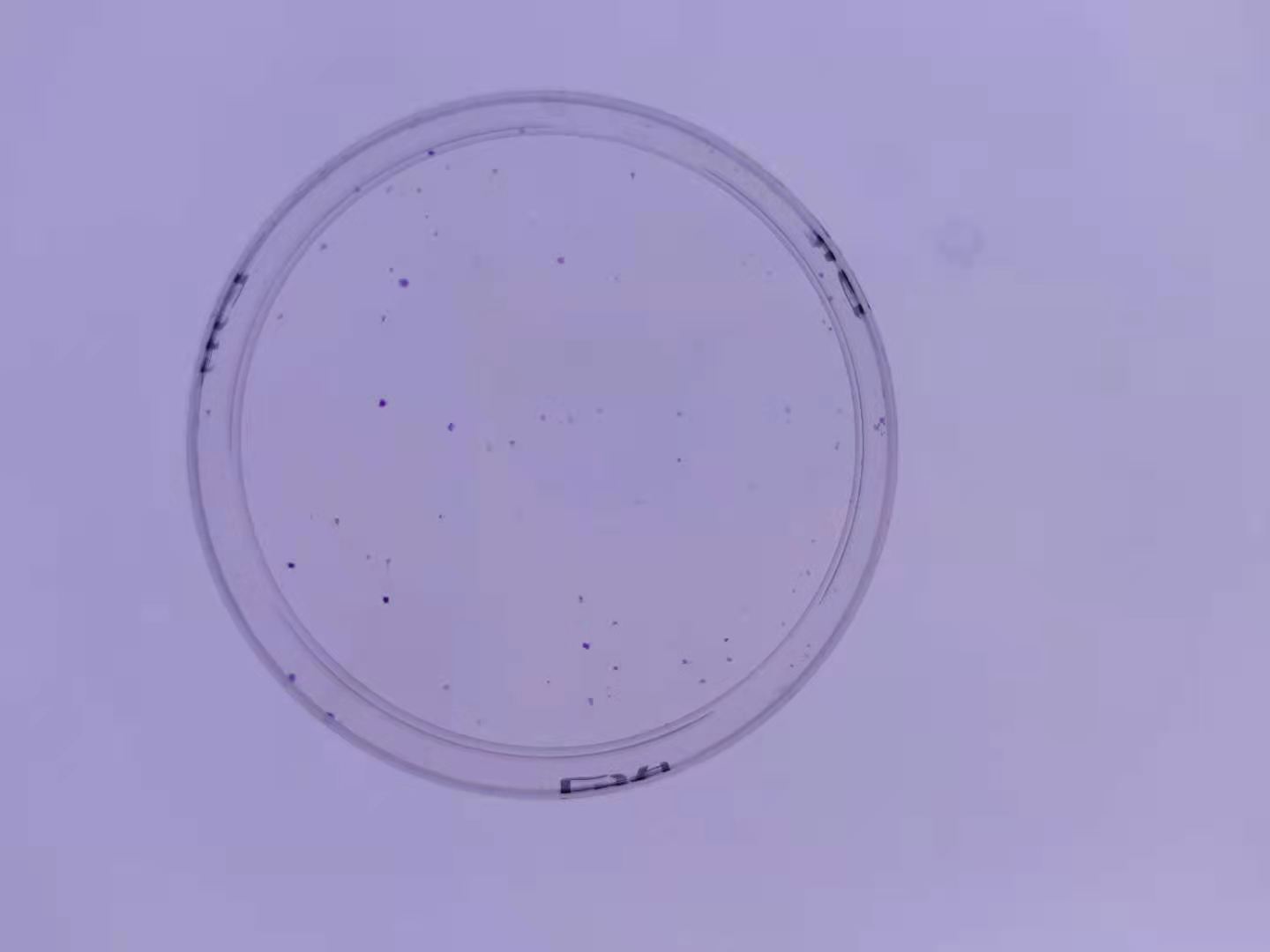

Supplement: Supplementary file 2 [file Data_Sheet_2.ZIP › 672571 fig4/pitures for fig4c/A431/6h/5 Gy.jpg]

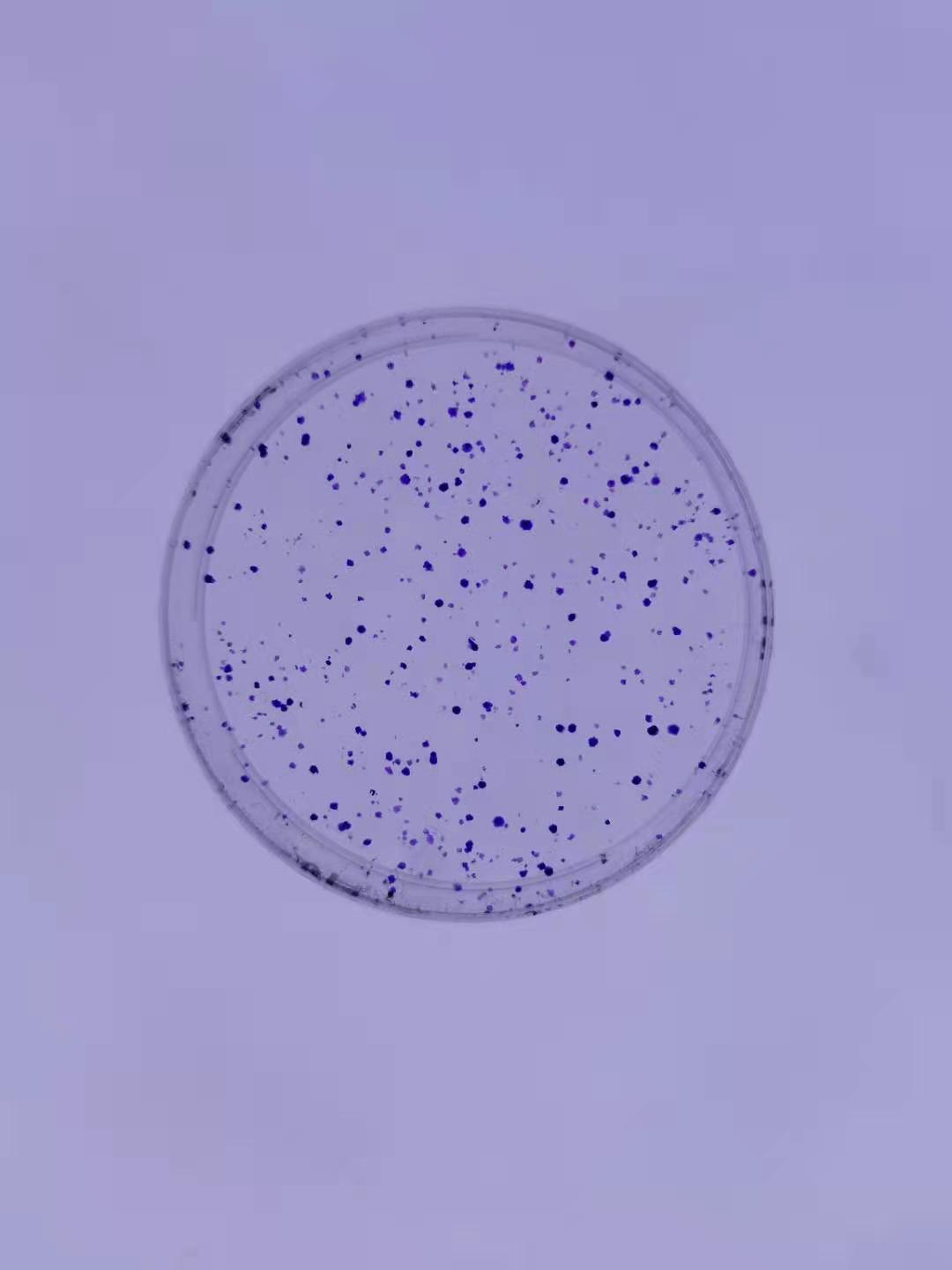

Supplement: Supplementary file 2 [file Data_Sheet_2.ZIP › 672571 fig4/pitures for fig4c/Cx26-/24 h/0 Gy.jpg]

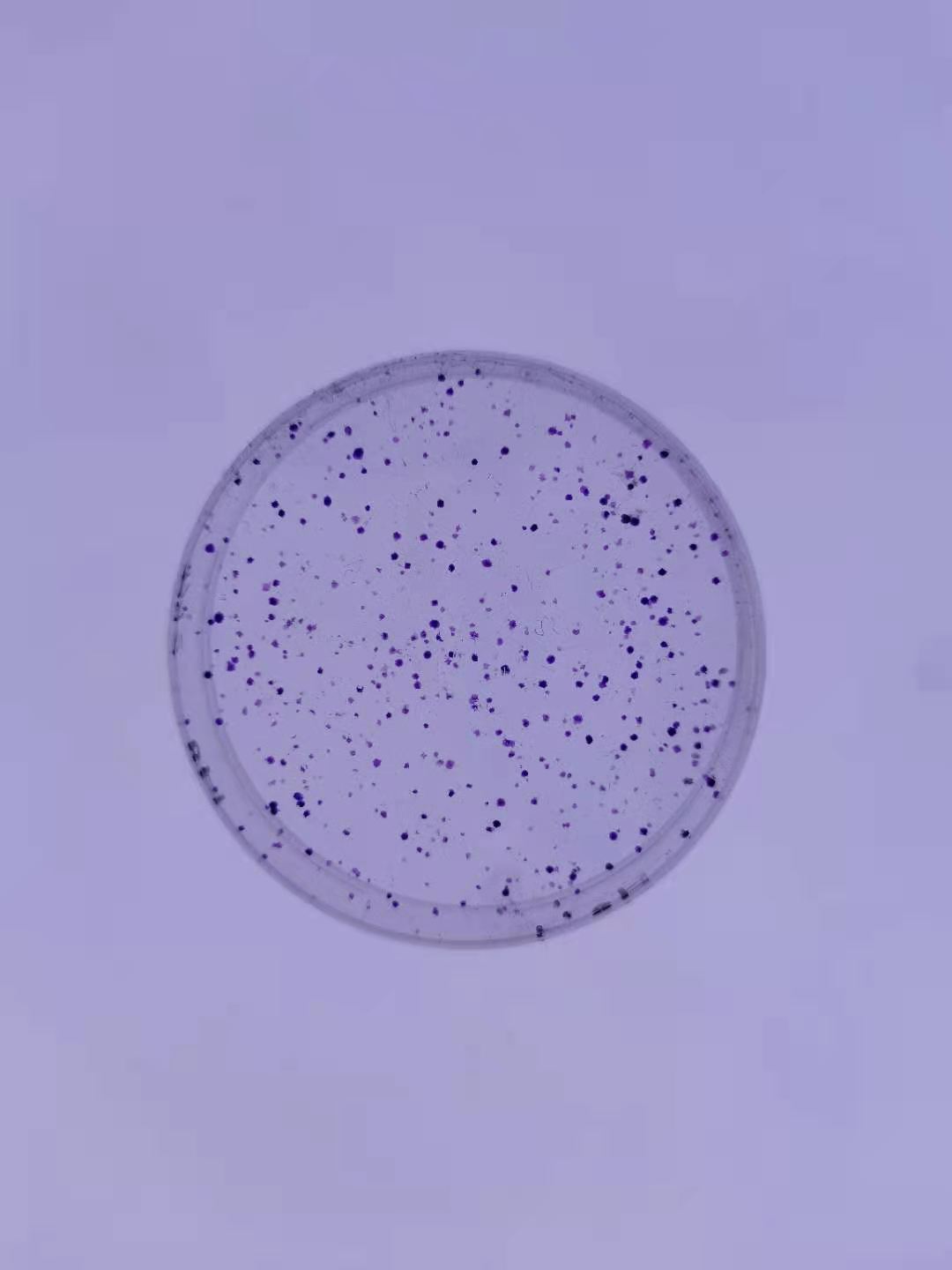

Supplement: Supplementary file 2 [file Data_Sheet_2.ZIP › 672571 fig4/pitures for fig4c/Cx26-/24 h/1 Gy.jpg]

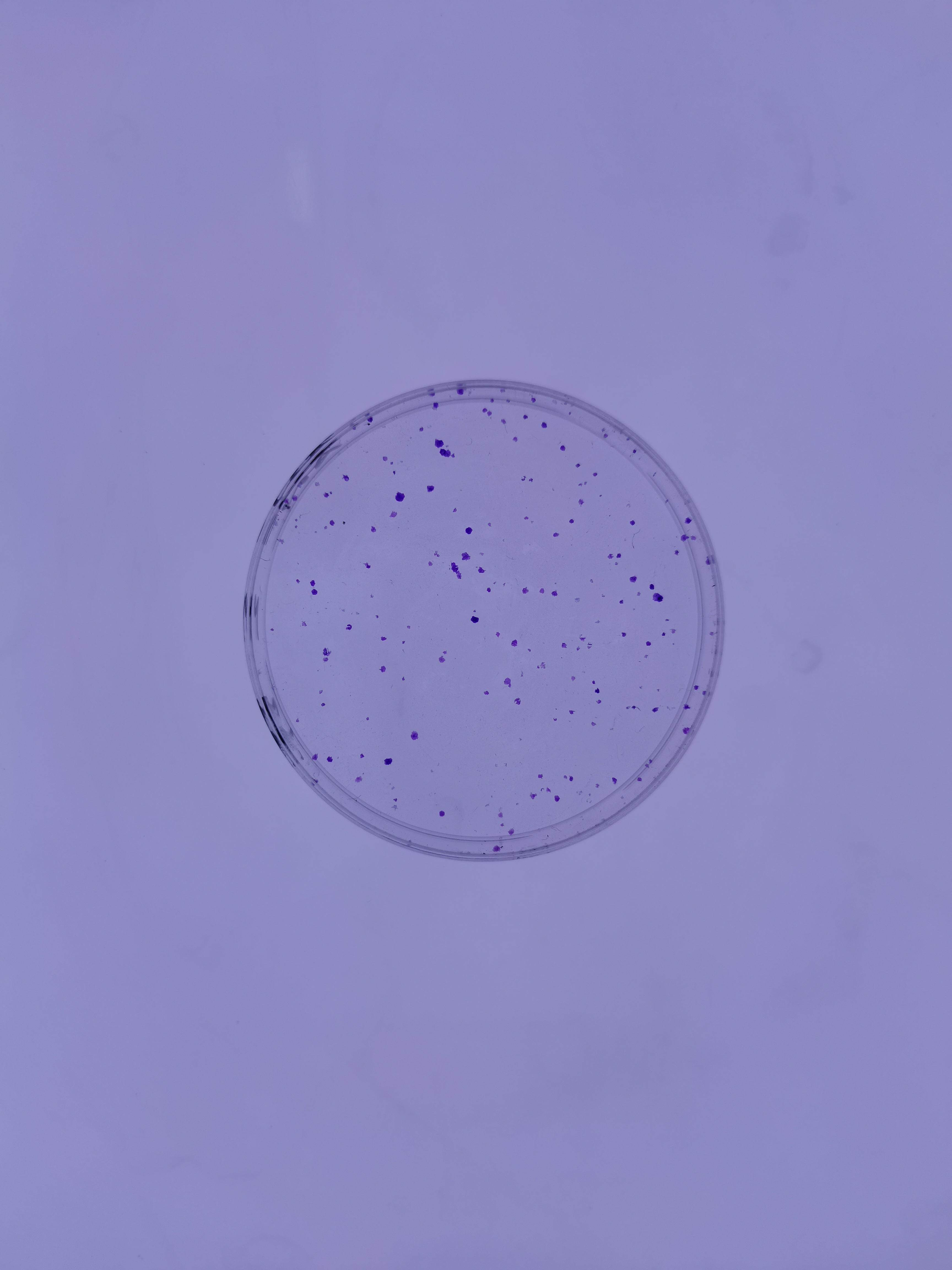

Supplement: Supplementary file 2 [file Data_Sheet_2.ZIP › 672571 fig4/pitures for fig4c/Cx26-/24 h/2 Gy.jpg]

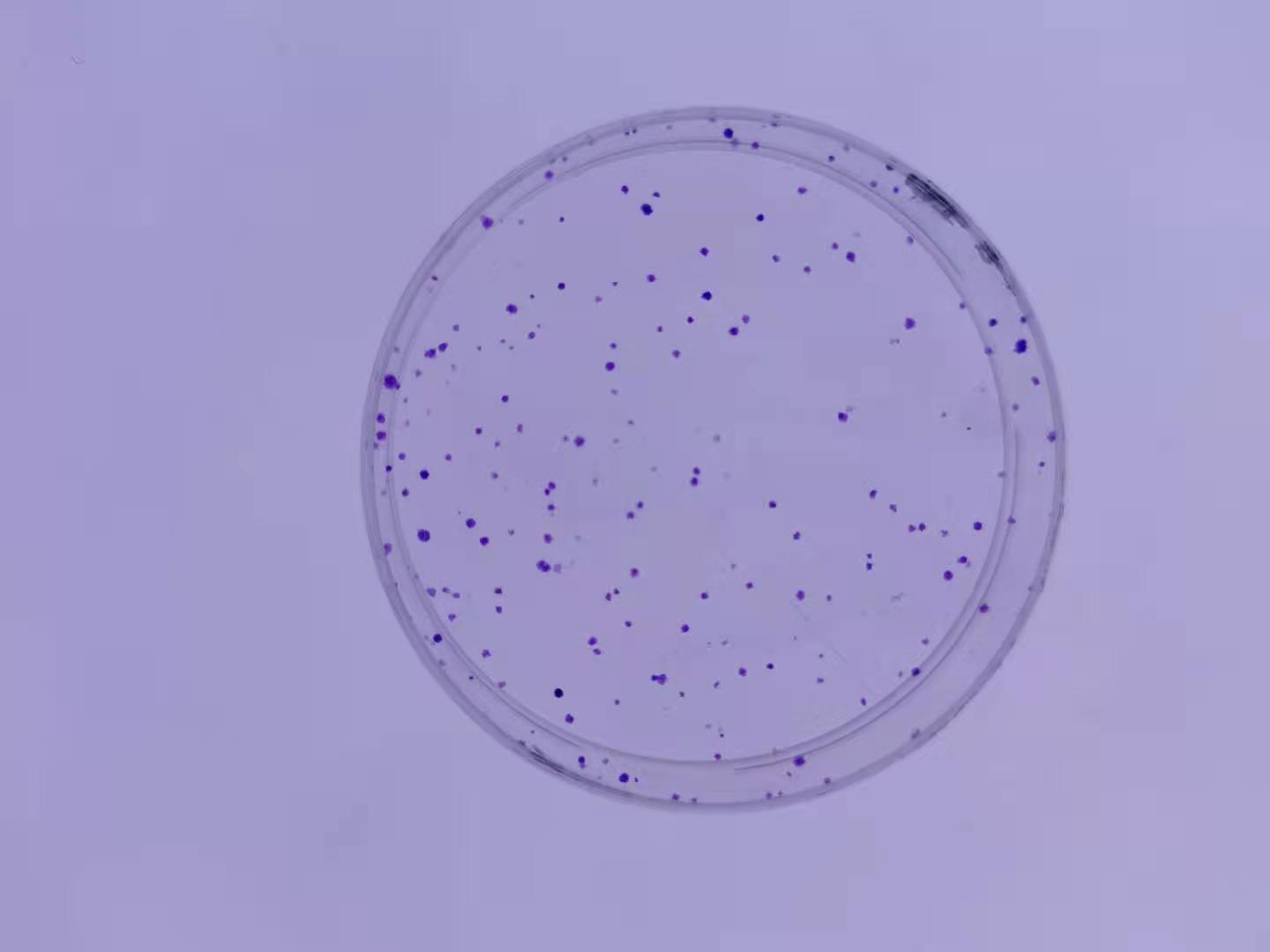

Supplement: Supplementary file 2 [file Data_Sheet_2.ZIP › 672571 fig4/pitures for fig4c/Cx26-/24 h/3 Gy.jpg]

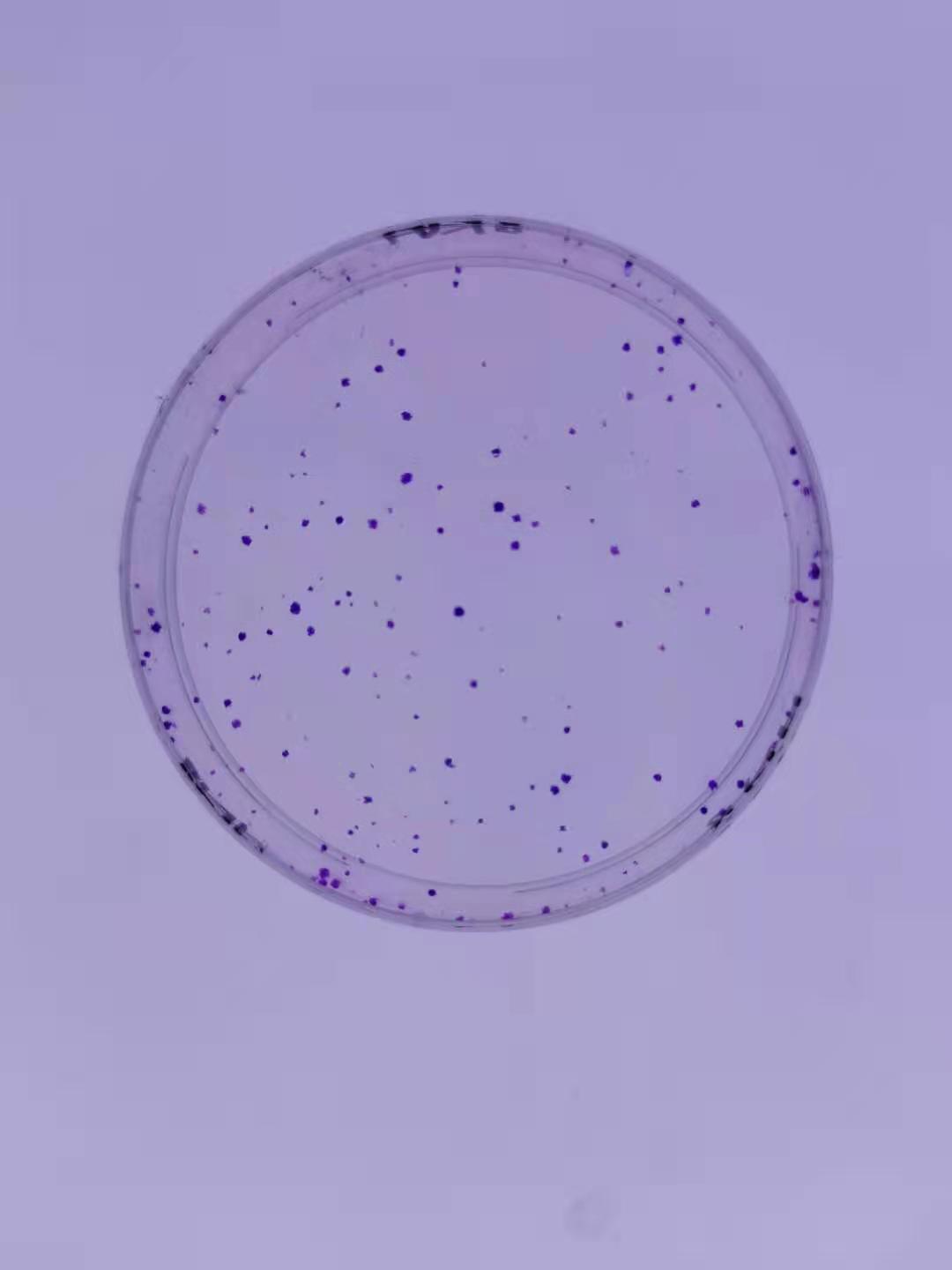

Supplement: Supplementary file 2 [file Data_Sheet_2.ZIP › 672571 fig4/pitures for fig4c/Cx26-/24 h/5 Gy.jpg]

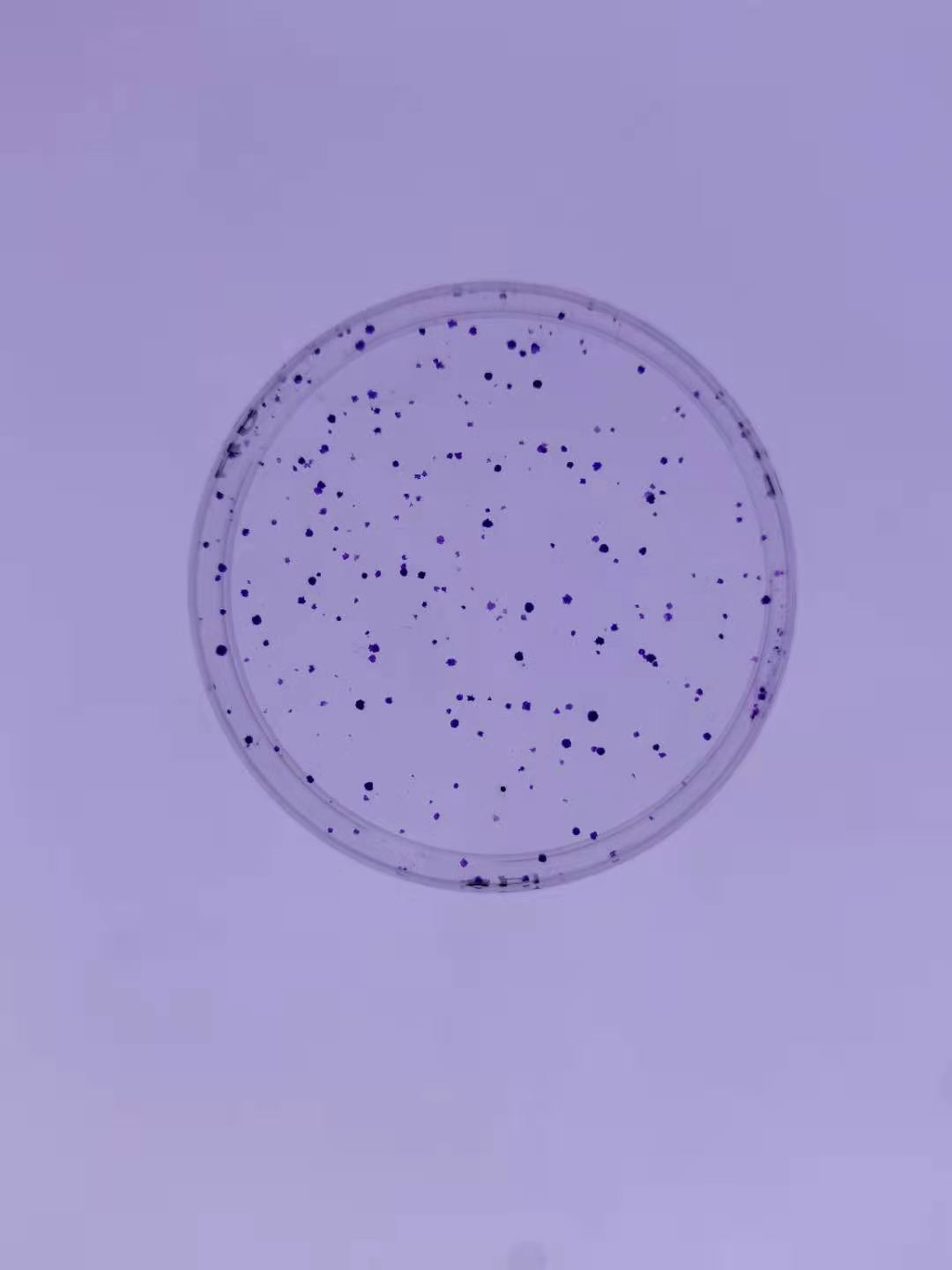

Supplement: Supplementary file 2 [file Data_Sheet_2.ZIP › 672571 fig4/pitures for fig4c/Cx26-/6 h/0 Gy.jpg]

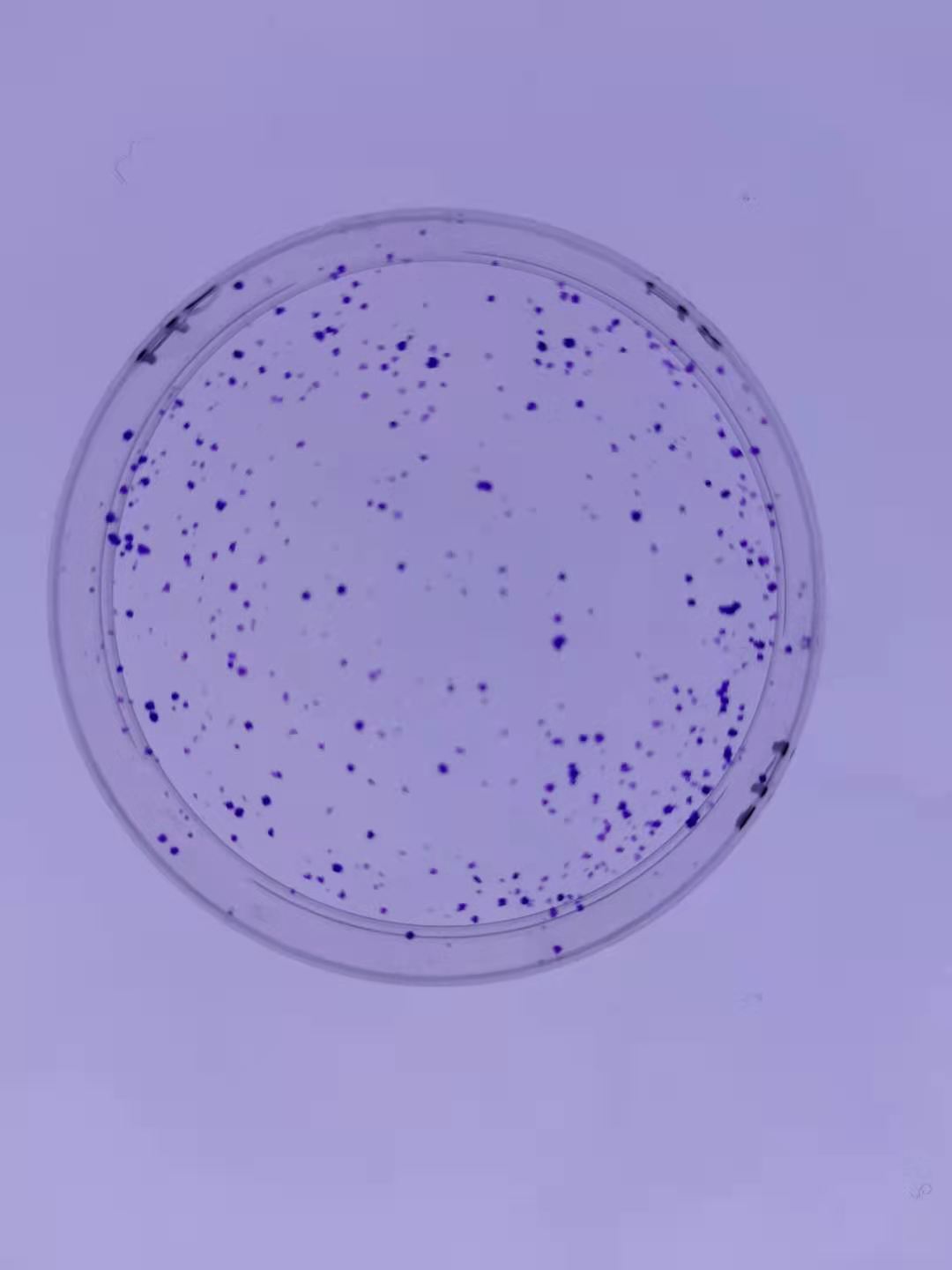

Supplement: Supplementary file 2 [file Data_Sheet_2.ZIP › 672571 fig4/pitures for fig4c/Cx26-/6 h/1 Gy.jpg]

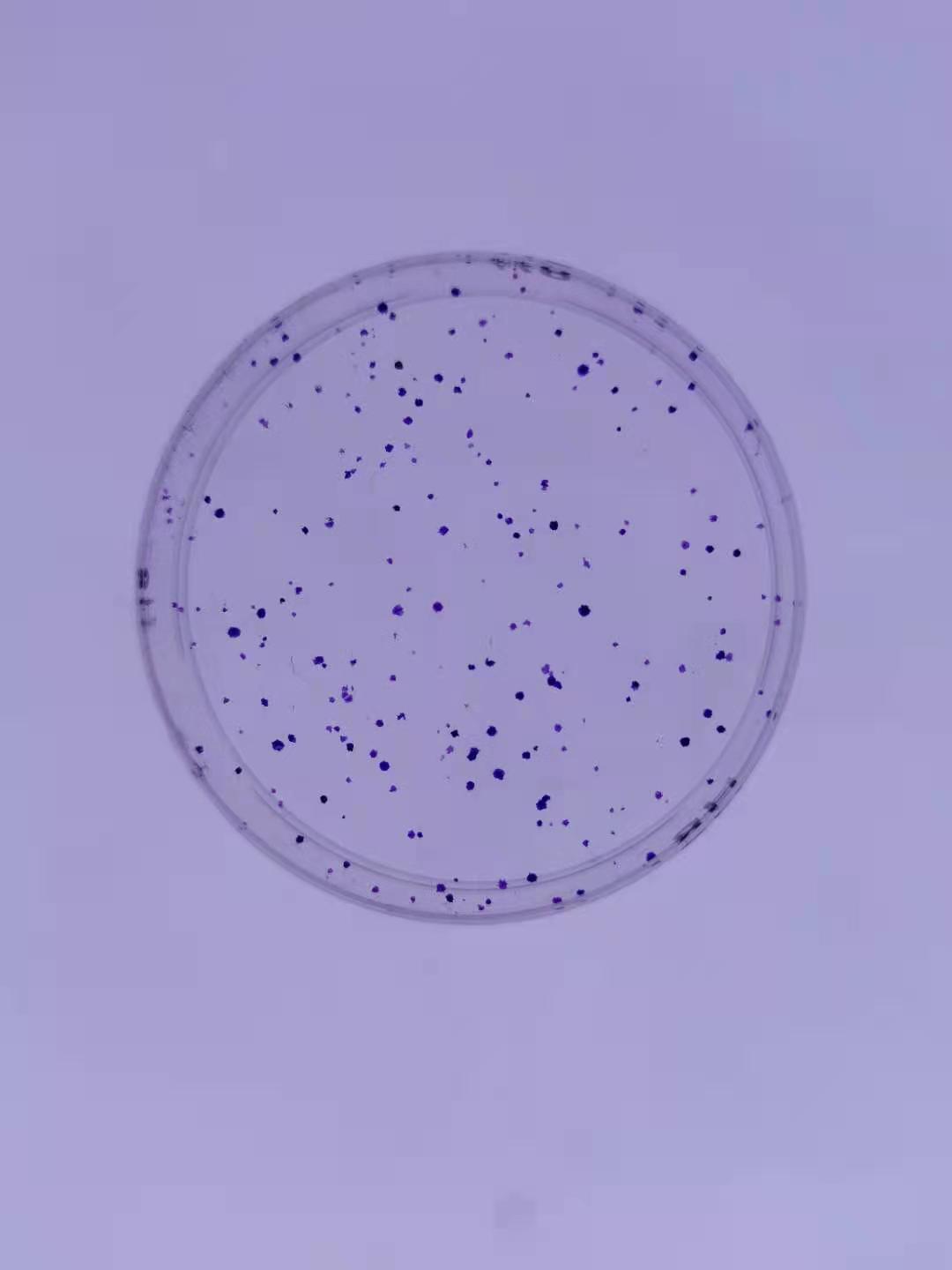

Supplement: Supplementary file 2 [file Data_Sheet_2.ZIP › 672571 fig4/pitures for fig4c/Cx26-/6 h/2 Gy.jpg]

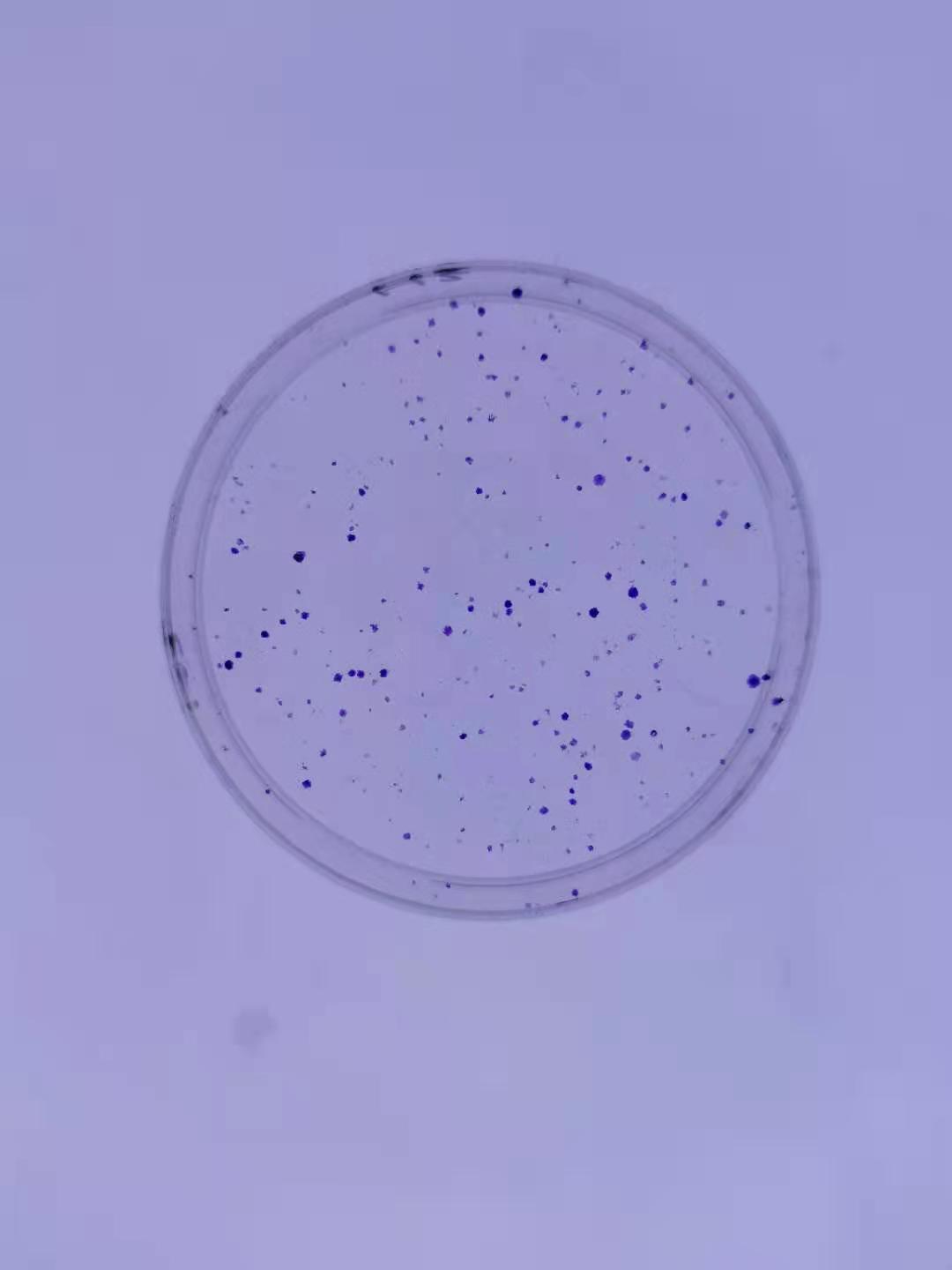

Supplement: Supplementary file 2 [file Data_Sheet_2.ZIP › 672571 fig4/pitures for fig4c/Cx26-/6 h/3 Gy.jpg]

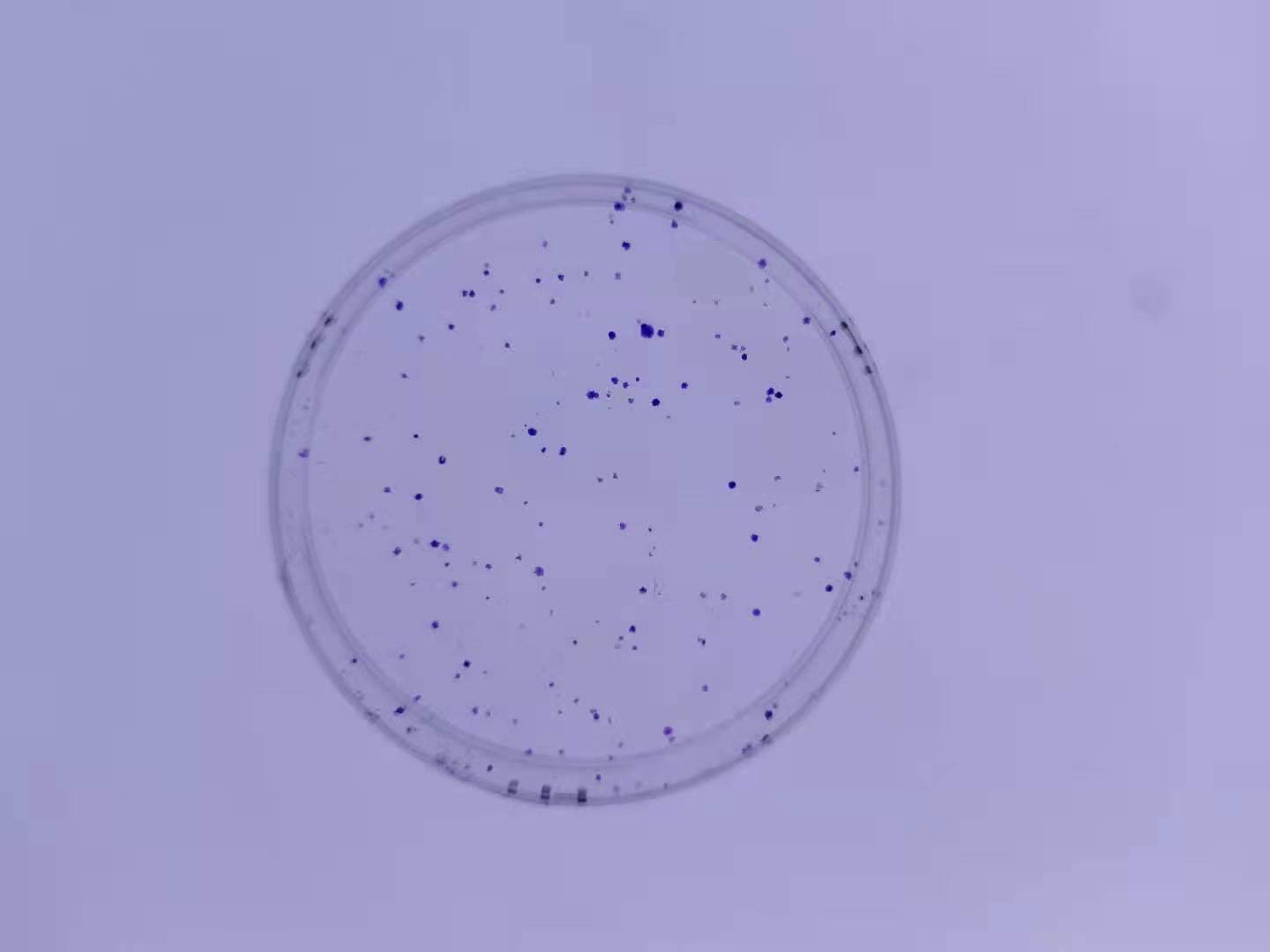

Supplement: Supplementary file 2 [file Data_Sheet_2.ZIP › 672571 fig4/pitures for fig4c/Cx26-/6 h/5 Gy.jpg]

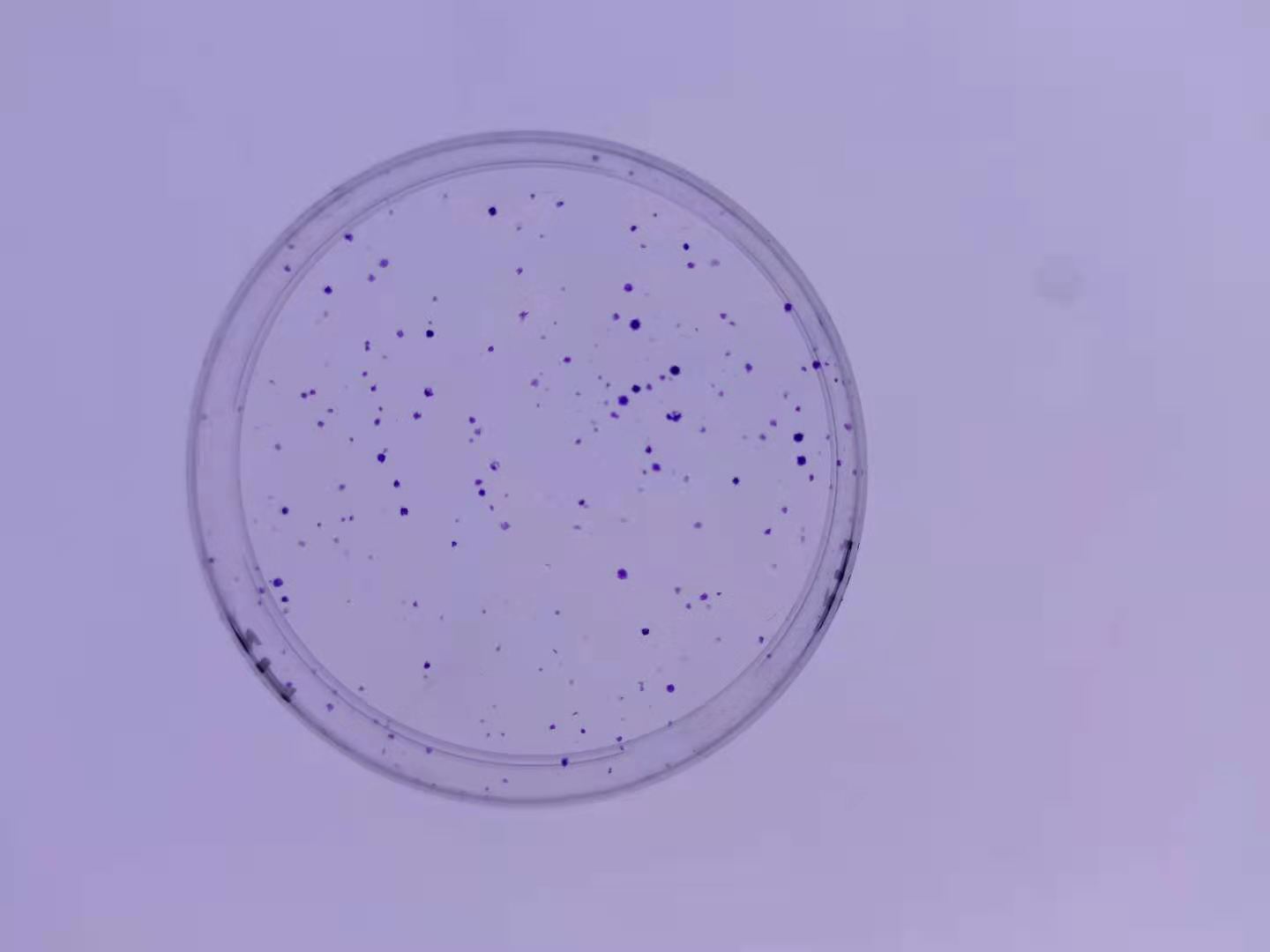

Supplement: Supplementary file 2 [file Data_Sheet_2.ZIP › 672571 fig4/pitures for fig4c/vector/24 h/0 Gy.jpg]

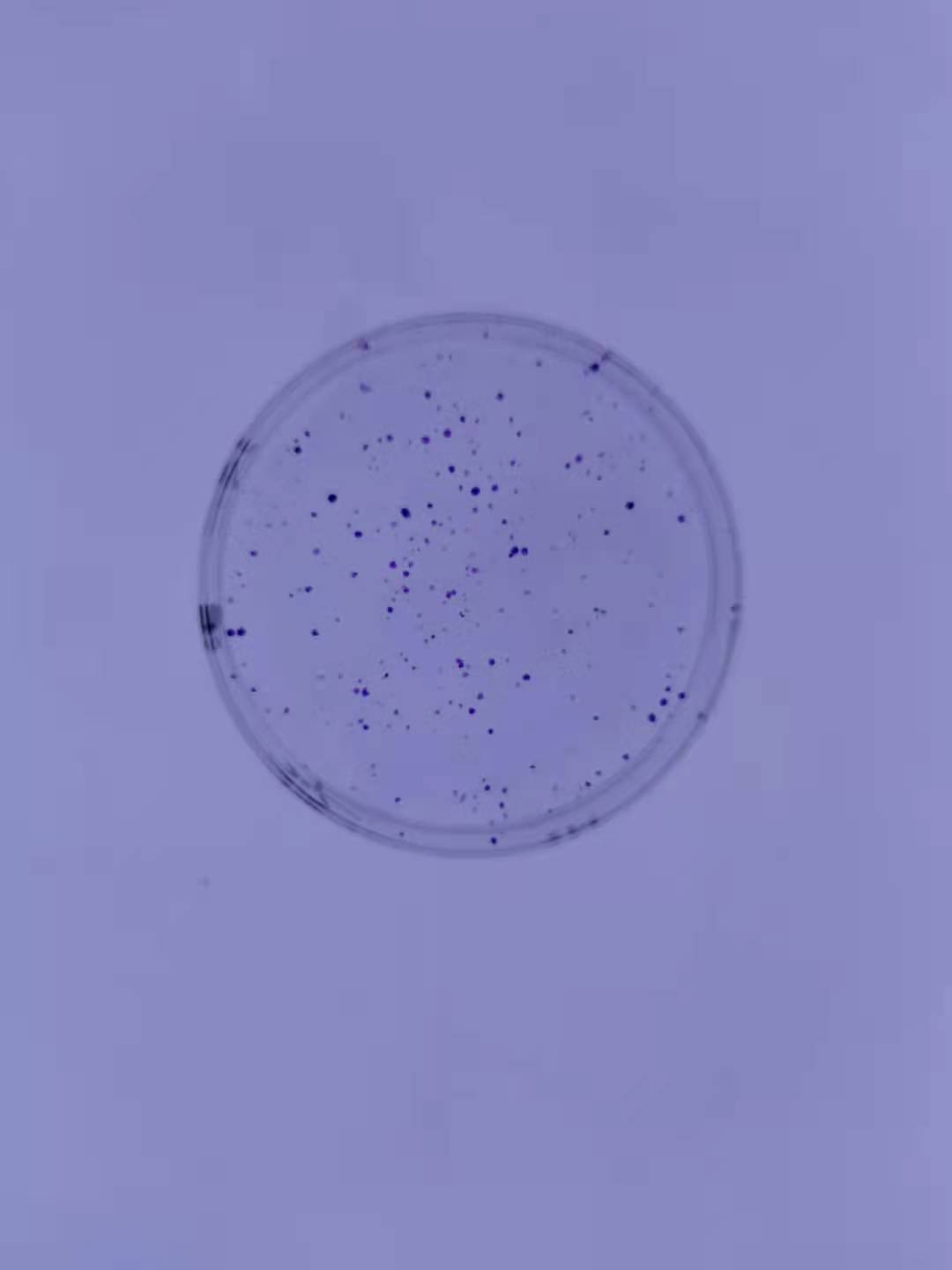

Supplement: Supplementary file 2 [file Data_Sheet_2.ZIP › 672571 fig4/pitures for fig4c/vector/24 h/1 Gy.jpg]

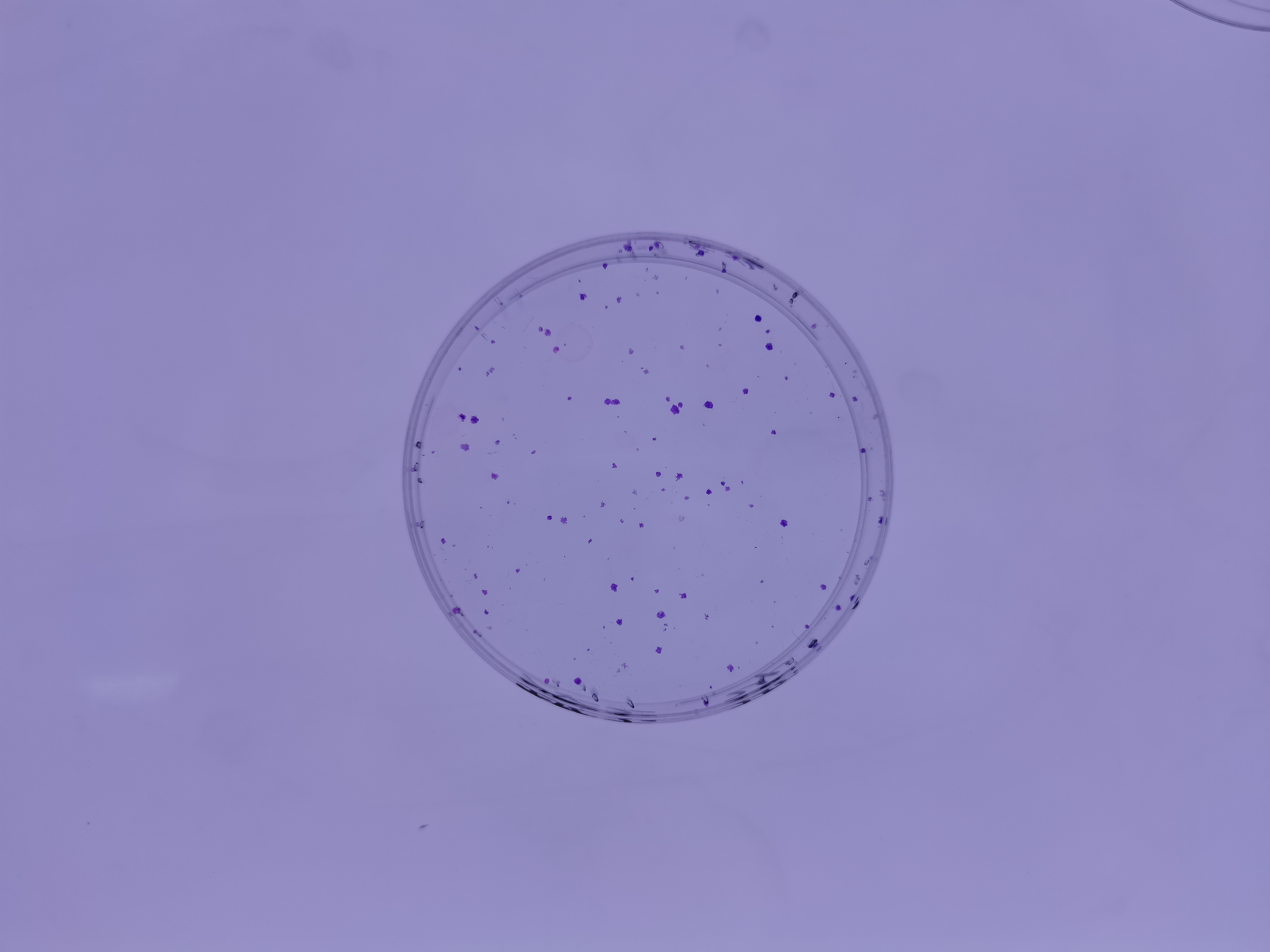

Supplement: Supplementary file 2 [file Data_Sheet_2.ZIP › 672571 fig4/pitures for fig4c/vector/24 h/2 Gy.jpg]

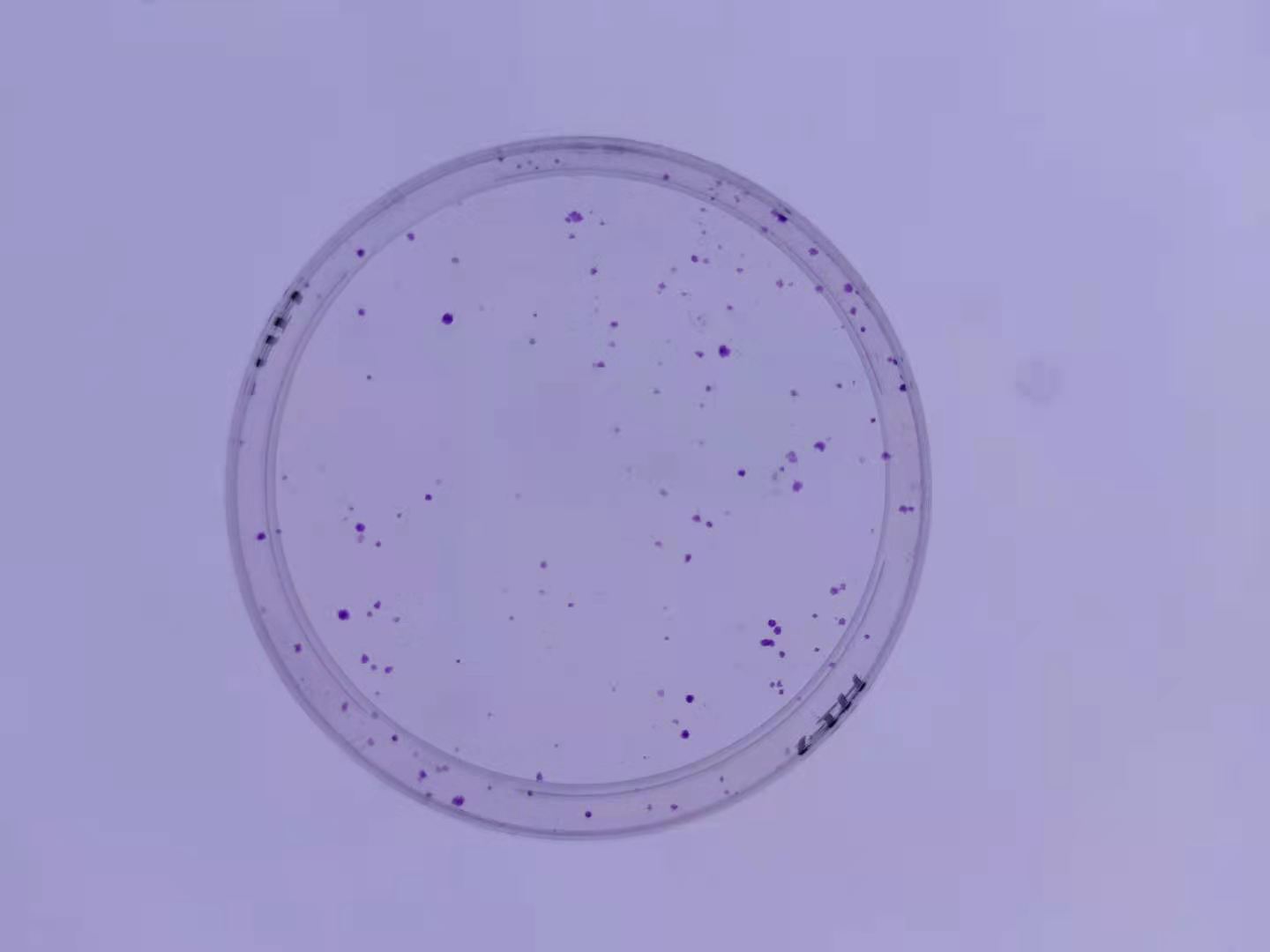

Supplement: Supplementary file 2 [file Data_Sheet_2.ZIP › 672571 fig4/pitures for fig4c/vector/24 h/3 Gy.jpg]

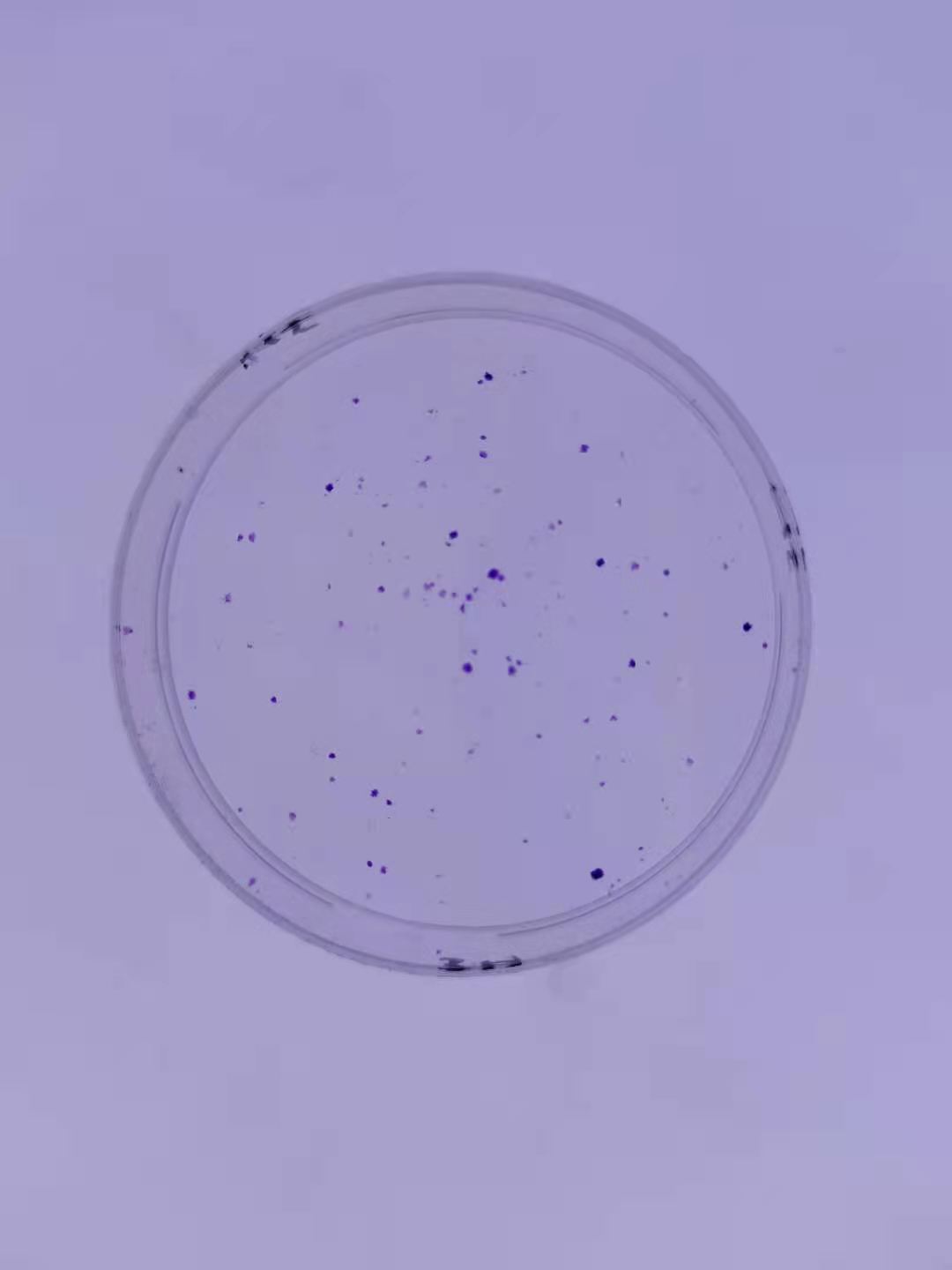

Supplement: Supplementary file 2 [file Data_Sheet_2.ZIP › 672571 fig4/pitures for fig4c/vector/24 h/5 Gy.jpg]

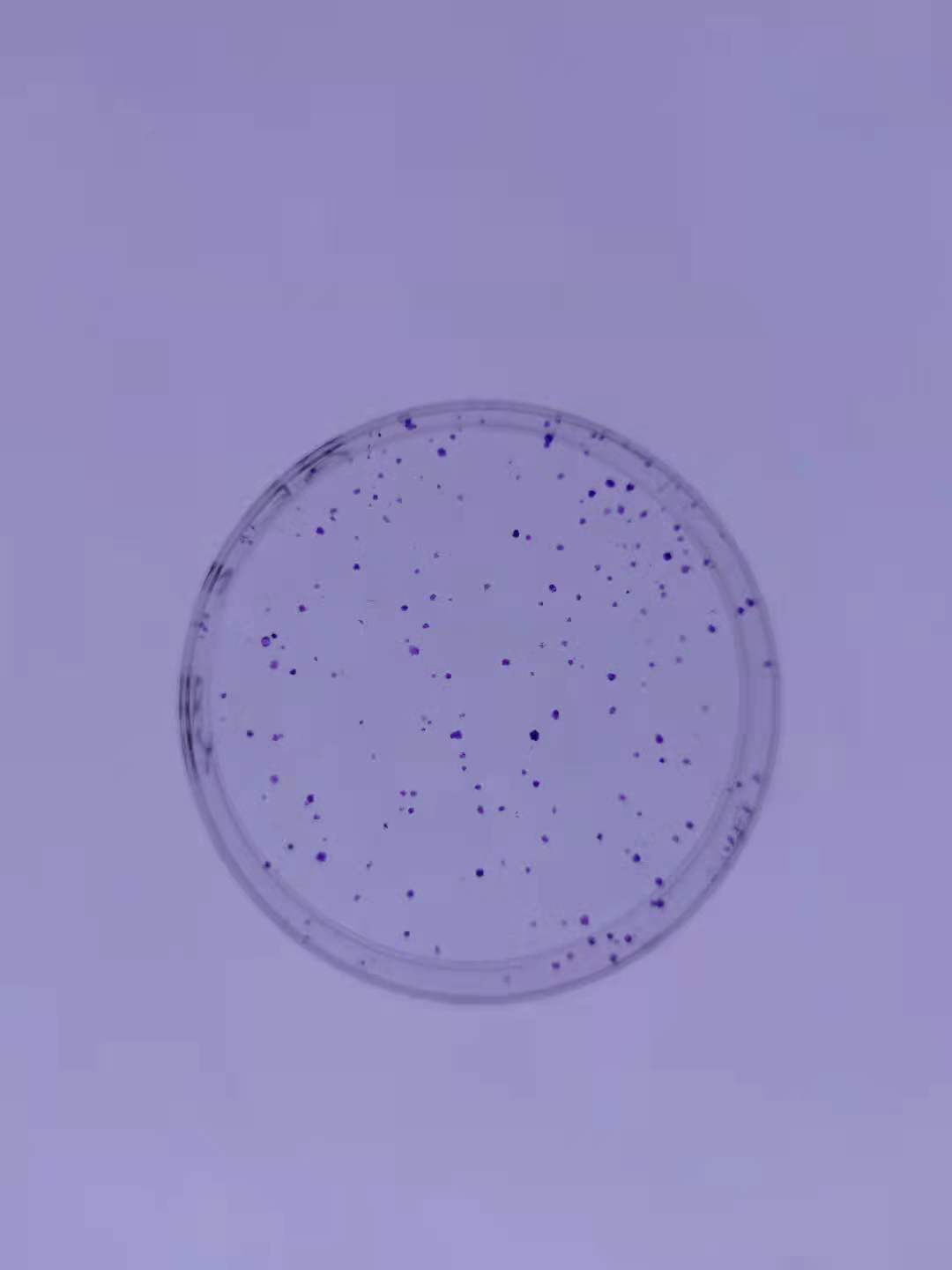

Supplement: Supplementary file 2 [file Data_Sheet_2.ZIP › 672571 fig4/pitures for fig4c/vector/6 h/0 Gy.jpg]

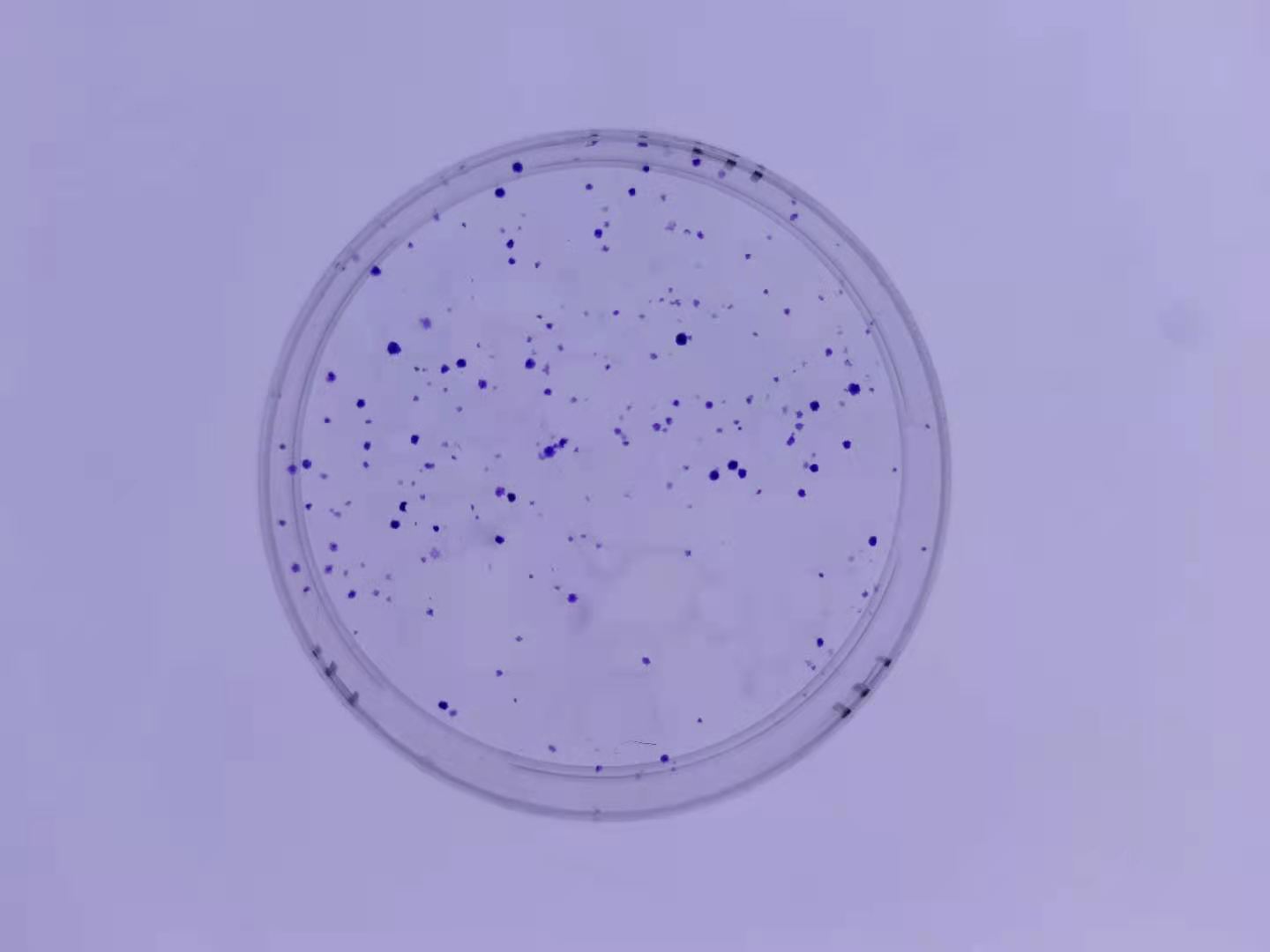

Supplement: Supplementary file 2 [file Data_Sheet_2.ZIP › 672571 fig4/pitures for fig4c/vector/6 h/1 Gy.jpg]

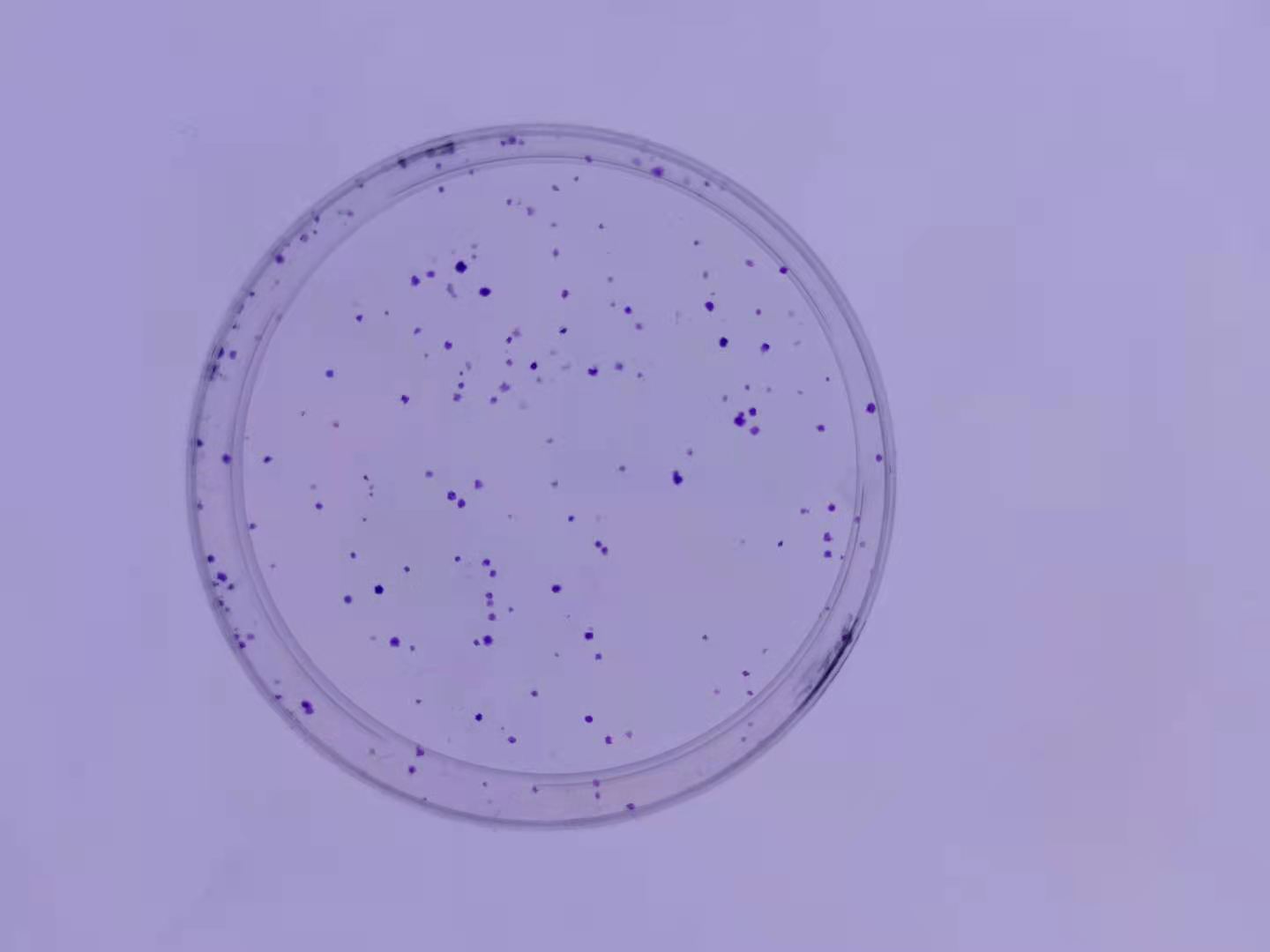

Supplement: Supplementary file 2 [file Data_Sheet_2.ZIP › 672571 fig4/pitures for fig4c/vector/6 h/2 Gy.jpg]

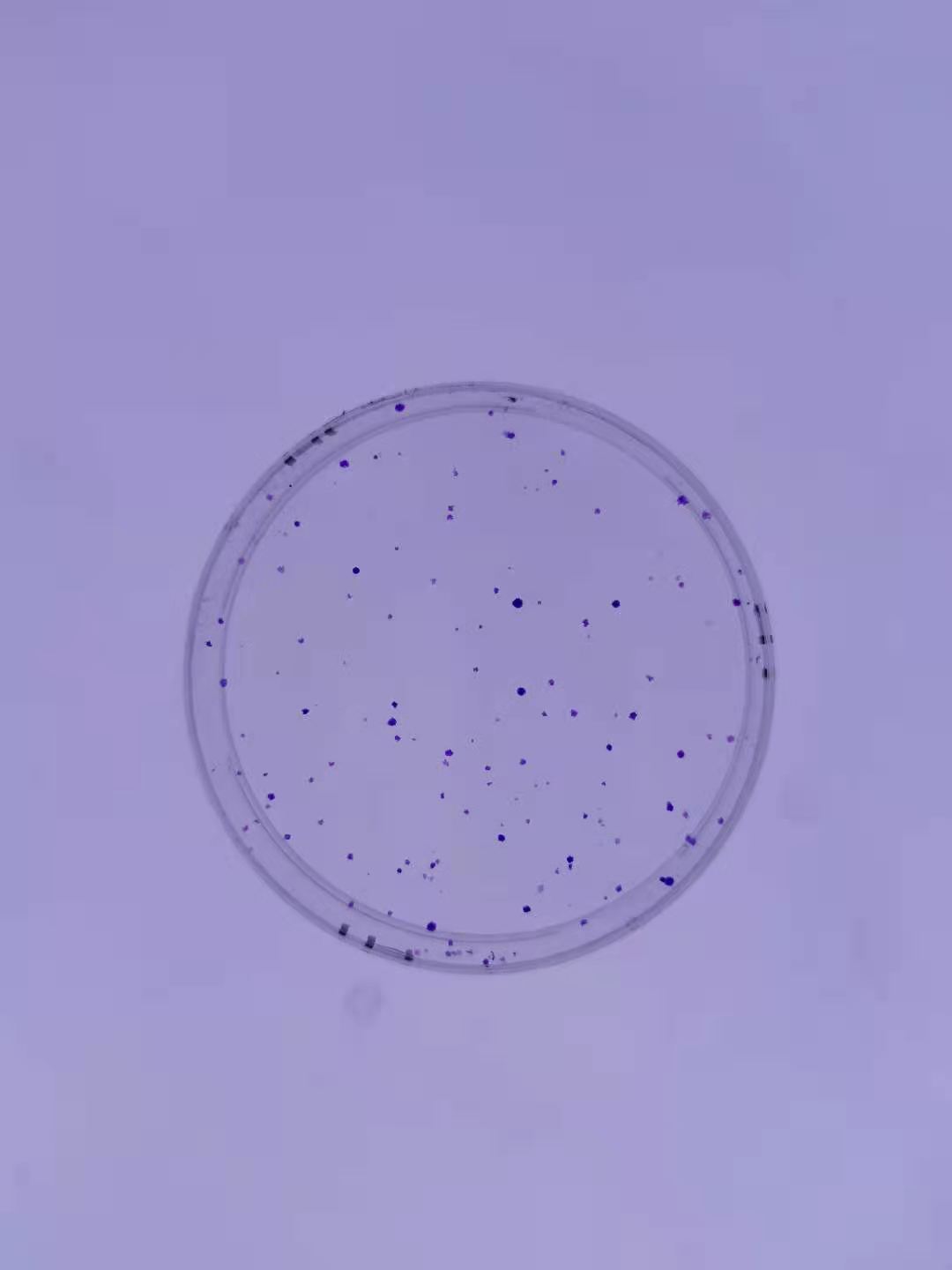

Supplement: Supplementary file 2 [file Data_Sheet_2.ZIP › 672571 fig4/pitures for fig4c/vector/6 h/3 Gy.jpg]

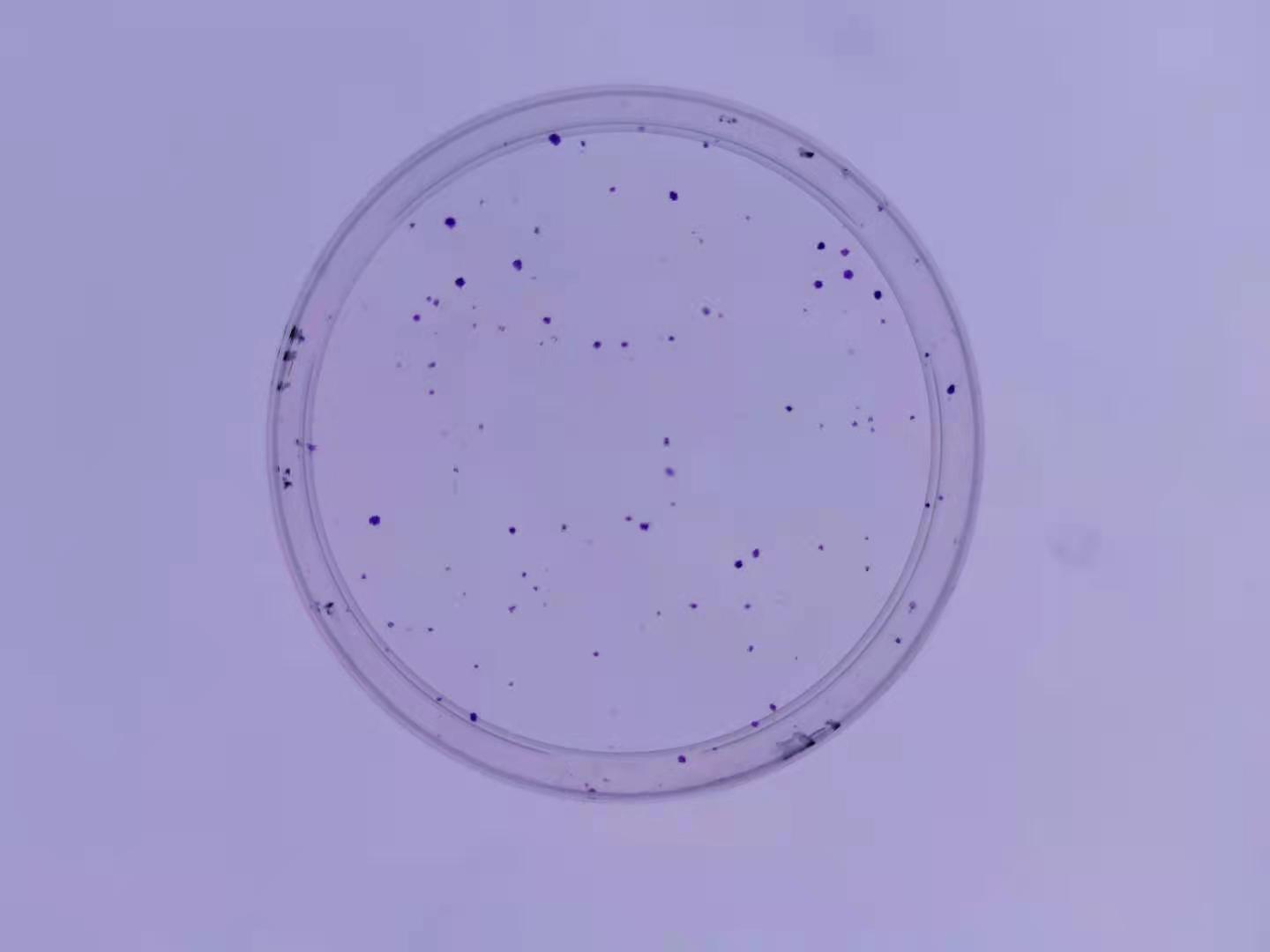

Supplement: Supplementary file 2 [file Data_Sheet_2.ZIP › 672571 fig4/pitures for fig4c/vector/6 h/5 Gy.jpg]

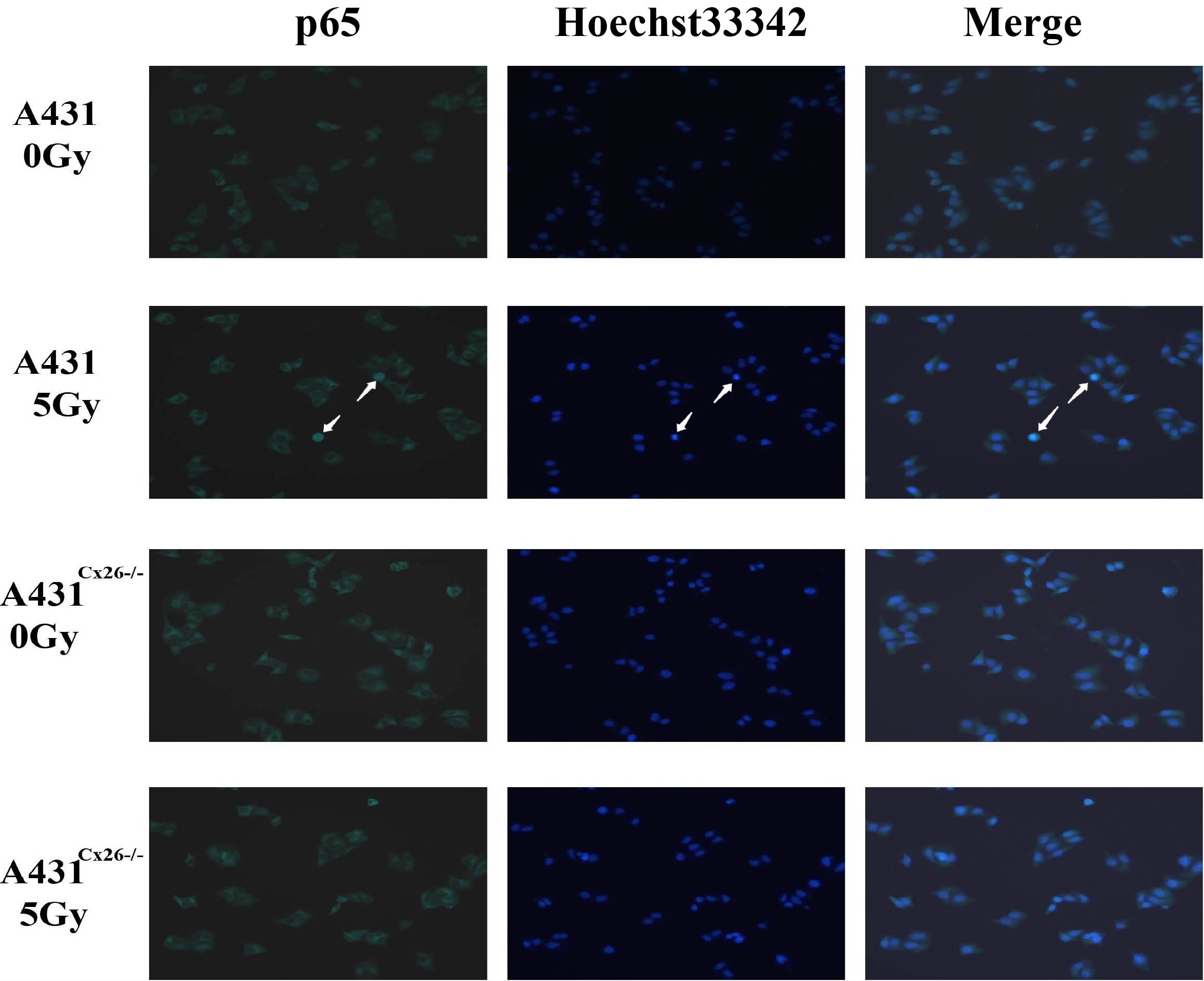

Supplement: Supplementary file 3 [file Image_1.JPEG]

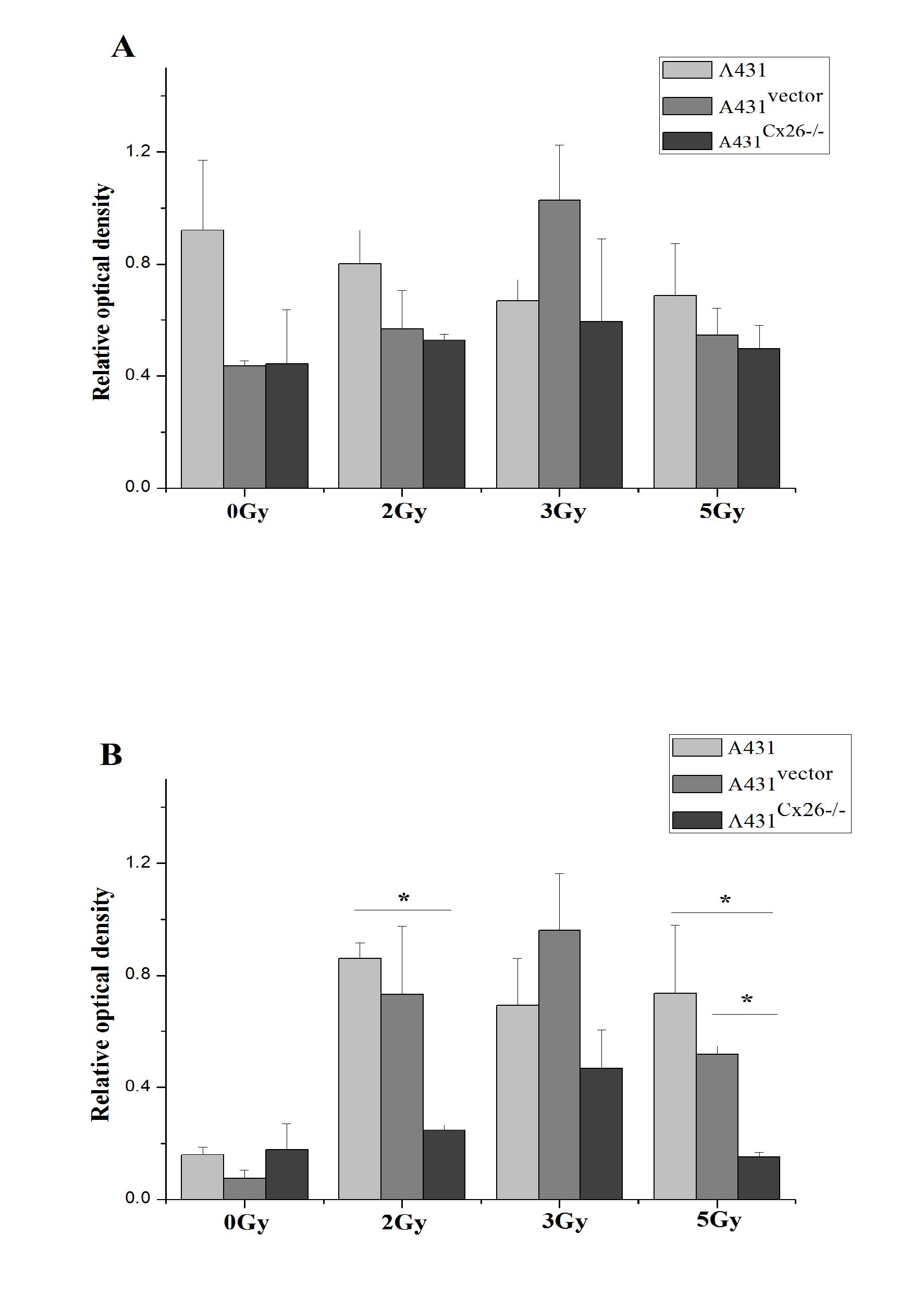

Supplement: Supplementary file 4 [file Image_2.JPEG]
