# Supplementary material for: Connexin26 Modulates the Radiosensitivity of Cutaneous Squamous Cell Carcinoma by Regulating the Activation of the MAPK/NF-κB Signaling Pathway
Source: Front Cell Dev Biol. 2021 Jul 5;9:672571. doi: 10.3389/fcell.2021.672571 (PMC8287175; doi:10.3389/fcell.2021.672571)
Supplement: Supplementary file 5 [file Presentation_1.pdf]

Supplementary Legends

Supplementary Figure1. The immunofluorescence figures of nuclear accumulation of NF-κB p65 in A431 and A431$^{Cx26-/-}$ cells 6 h after 5Gy X-ray irradiation.

In the western blot experiment, the expressions of NF-κB p65 were significantly different between A431 cells and A431$^{Cx26-/-}$ cells 6 h after 5Gy X-ray irradiation. Therefore, in the immunofluorescence experiment, we selected the X-ray dose of 5Gy. The time point is still 6 h after irradiation. In the immunofluorescence experiment, we found that the nuclear accumulations of NF-κB p65 showed significantly different between sham and irradiated A431 cells. But the nuclear accumulations of NF-κB p65 were not significantly different between sham and irradiated A431 $^{Cx26-/-}$ cells. We think that these results are related to the low expression of Cx26 in A431$^{Cx26-/-}$ cells, which proves that different expression levels of Cx26 have a certain regulatory effect on NF-κB signaling pathway.

Supplementary Figure2. The relative optical density analysis results of p-ERK and ERK expression in A431, A431^{Vector} and A431$^{Cx26-/-}$ cells after irradiation. (A) The comparison of ERK protein expression levels of A431 cells, A431^{vector} cells and A431$^{Cx26-/-}$ cells. (B) The comparison of p-ERK expression levels of A431 cells, A431^{vector} cells and A431$^{Cx26-/-}$ cells.

As can be seen from the supplementary Figure 2, after irradiation, the ERK expression

level of A431$^{Cx26-/-}$ cell was generally lower than that of A431 and A431^{vector} cell. Moreover, when the irradiation dose was 2Gy and 5Gy, the expression of p-ERK in A431$^{Cx26-/-}$ cell was significantly lower than that in A431 and A431^{vector} cell. These results indicate that the knock-out of Cx26 has a certain influence on the expression and phosphorylation of ERK in A431 cells.
